# Boosting Wnt activity during colorectal cancer progression through selective hypermethylation of Wnt signaling antagonists

**DOI:** 10.1186/1471-2407-14-891

**Published:** 2014-11-29

**Authors:** Ana-Luisa Silva, Sarah N Dawson, Mark J Arends, Kiran Guttula, Nigel Hall, Ewen A Cameron, Tim H-M Huang, James D Brenton, Simon Tavaré, Mariann Bienz, Ashraf EK Ibrahim

**Affiliations:** Department of Pathology, Division of Molecular Histopathology, University of Cambridge, Addenbrooke’s Hospital, Hills Road, Cambridge, CB2 2QQ UK; Cambridge Clinical Trials Unit, Cambridge University Hospitals NHS Foundation Trust, Hills Road, Cambridge, CB2 0QQ UK; Cancer Research UK Cambridge Institute, Li Ka Shing Centre, Robinson Way, Cambridge, CB2 0RE UK; University of Edinburgh Division of Pathology, Edinburgh Cancer Research Centre, Institute of Genetics & Molecular Medicine, Western General Hospital, Crewe Road South, Edinburgh, EH4 2XR UK; Cambridge Colorectal Unit, Department of Surgery, Addenbrooke’s Hospital, Box 201, Hills Road, Cambridge, CB2 2QQ UK; Gastroenterology, Addenbrooke’s Hospital, Hills Road, Cambridge, CB2 2QQ UK; University of Texas Health Science Center, 7979 Wurzbach Road, San Antonio, Texas 78229-3900 USA; MRC, Laboratory of Molecular Biology, Hills Road, Cambridge, CB2 0QH UK

## Abstract

**Background:**

There is emerging evidence that Wnt pathway activity may increase during the progression from colorectal adenoma to carcinoma and that this increase is potentially an important step towards the invasive stage. Here, we investigated whether epigenetic silencing of Wnt antagonists is the biological driver for this increased Wnt activity in human tissues and how these methylation changes correlate with MSI (Microsatelite Instability) and CIMP (CpG Island Methylator Phenotype) statuses as well as known mutations in genes driving colorectal neoplasia.

**Methods:**

We conducted a systematic analysis by pyrosequencing, to determine the promoter methylation of CpG islands associated with 17 Wnt signaling component genes. Methylation levels were correlated with MSI and CIMP statuses and known mutations within the *APC*, *BRAF* and *KRAS* genes in 264 matched samples representing the progression from normal to pre-invasive adenoma to colorectal carcinoma.

**Results:**

We discovered widespread hypermethylation of the Wnt antagonists *SFRP1, SFRP2, SFRP5, DKK2*, *WIF1* and *SOX17* in the transition from normal to adenoma with only the Wnt antagonists *SFRP1*, *SFRP2*, *DKK2* and *WIF1* showing further significant increase in methylation from adenoma to carcinoma. We show this to be accompanied by loss of expression of these Wnt antagonists, and by an increase in nuclear Wnt pathway activity. Mixed effects models revealed that mutations in *APC*, *BRAF* and *KRAS* occur at the transition from normal to adenoma stages whilst the hypermethylation of the Wnt antagonists continued to accumulate during the transitions from adenoma to carcinoma stages.

**Conclusion:**

Our study provides strong evidence for a correlation between progressive hypermethylation and silencing of several Wnt antagonists with stepping-up in Wnt pathway activity beyond the APC loss associated tumour-initiating Wnt signalling levels.

**Electronic supplementary material:**

The online version of this article (doi:10.1186/1471-2407-14-891) contains supplementary material, which is available to authorized users.

## Background

Colorectal cancer (CRC) is the second most common cause of cancer-related death in the UK accounting for approximately 10% of all cancer deaths [1]. Known genetic and epigenetic aberrations drive the formation of a benign adenoma, and its progression to full-blown colorectal carcinoma [2–4]. In particular, >90% of CRC exhibit mutations in the Adenomatous polyposis coli (APC) gene and in other Wnt signaling components that result in hyperactivation of the Wnt pathway, and these mutations are the earliest known genetic alterations, indicating that they represent the initiating event in the path to CRC [2, 5, 6]. APC is a crucial negative regulator of the Wnt pathway: as a component of the cytoplasmic Axin degradasome complex, APC promotes the proteasomal degradation of the Wnt effector β-catenin; if this complex is dysfunctional as a consequence of mutational inactivation of *APC*, β-catenin accumulates to high levels and translocates into the nucleus where it operates a transcriptional switch [7]. One of its direct transcriptional targets is *c-MYC*, whose product is pivotal in driving malignancy in both mice and humans [8, 9].

The role of Wnt signaling in initiating CRC is therefore well documented. However, it is less clear whether hyperactive Wnt signaling is also required for the progression from adenoma to carcinoma. Recent evidence suggests that this may be the case, based on xenograft models in mice and on the observation that β-catenin accumulates to high levels in CRC samples [10]. Similarly, our own data revealed that the levels of nuclear β-catenin tend to be elevated in early adenomas, but show a further surge in levels in carcinomas, indicating that the Wnt signaling levels increase during cancer progression [11]. Furthermore, epigenetic inactivation of extracellular Wnt signaling antagonists has also been observed in colorectal carcinomas, which could boost Wnt signaling to levels above those caused by the initial mutational inactivation of *APC* [12]. All these are indications that the level of Wnt signaling increases from the adenoma to the carcinoma stage, implying that the sustained (or increased) activity of β-catenin could be critical throughout CRC progression.

Epigenetic silencing by DNA hypermethylation of associated CpG islands is a common mechanism by which genes are inactivated during cancer development. In CRC, epigenetic silencing has been observed not only for negative regulators of Wnt signaling upstream in the pathway, such as the extracellular Wnt inhibitors *SFRP1*, *SFRP2*, *SFRP3*, *SFRP4*, *SFRP5*, *WIF1*, *DKK1* and *DKK3* [12–19] and DACT3 [20] but also for negative regulators acting further downstream in the pathway, including *APC* [21], *AXIN2* [22], *CDH1* [23] and *SOX17* [24]. However, none of these studies entailed a systematic and comprehensive characterization of the synchronous changes of DNA methylation patterns of Wnt antagonists and particularly how these changes affect Wnt signalling transcriptional output through the neoplastic progression from the pre-invasive adenoma stage to the invasive carcinoma stage. In addition, no data is available on: (i) the association of the methylation changes of Wnt antagonists with microsatalite instability (MSI)/CpG island methylator phenotype (CIMP) statuses nor (ii) its relationship to known mutations in genes involved in the early progression of colorectal neoplasia.

We thus set out to analyze systematically the CpG methylation patterns at gene promoters of 17 Wnt signaling components (Additional file 1) and correlate these patterns with expression levels of nuclear β-catenin and two well-established Wnt target genes (*AXIN2* and *c-MYC*). In addition, we examined the correlation of the methylation patterns of these Wnt genes with MSI/CIMP statuses, the presence of known mutations within *APC*, *BRAF* and *KRAS*, in a large set of matching normal, hyperplastic or adenomatous polyps, primary and metastatic adenocarcinoma tissue samples. Finally, we asked if the identified patterns of methylation of these Wnt genes impact patients’ survival.

## Methods

### Clinical sample collection

Two independent sample sets were collected from colectomy surgical specimens (the clinicopathological characteristics are summarised in Additional file 2). The first set of samples (CRC1, n = 86) was obtained from 48 patients with invasive colorectal primary carcinoma with or without evidence of metastatic cancer deposits. The CRC1 sample set comprised normal colonic mucosa (n = 20), primary (n = 51) and liver metastatic (n = 15) adenocarcinomas. The second set (CRC2) comprised 172 samples from a set of 49 patients presenting with synchronous adenoma and invasive carcinoma. Normal tissue samples (n = 73) were collected at 5 cm and 20 cm (where available) away from the carcinoma and were defined as high-risk normal mucosa (HRN), samples from hyperplastic polyps (n = 13), adenomatous polyps (n = 39) and primary adenocarcinoma (n = 47) were also collected. For comparison, we collected normal mucosa from patients undergoing colectomy for diverticular disease (n = 6) who had no previous or present history of CRC. These samples were defined as low-risk normal mucosa (LRN). The histological features and cellularity of all tissue samples were assessed by microscopic examination of tissues sampled immediately adjacent to the site of sampling fresh tissues by a histopathologist with interest in CRC (AEKI). Samples were collected within the Histopathology Department and the Tissue Bank facility within Addenbrooke’s Hospital (Cambridge, UK) and a subset of CRC1 cases (n = 37) was obtained from Ohio State University (OSU) where colonic normal, primary and metastatic adenocarcinoma tissue samples were microdissected. Ethical approval for all the work conducted was obtained from both OSU institutional review board and Cambridgshire local research ethics committee (LREC ref. 04/Q0108/125 and 06/Q0108/307). Written informed consent was obtained from the patient for the publication of this report and any accompanying images.

### DNA extraction and bisulfite modification

High molecular weight DNA was isolated using a proteinase K/phenol extraction method. Sodium bisulfite conversion of DNA was performed using the EZ DNA Methylation-Gold Kit (ZymoResearch, Cambridge, UK), following the manufacturer’s protocol.

### Total RNA extraction and real-time PCR

Tissue samples were left in RNA*later*-ICE at −20°C for at least 24 hours prior to extraction. RNA was extracted using both chloroform and column based protocols as described in Additional file 2. Quality and quantity of the extracted RNA was verity before storage at −80°C. Full details of cDNA synthesis and Real-Time Quantitative PCR (qRT-PCR) are described in Additional file 3. We used the Pfaffl method to calculate the expression fold change [25].

### Pyrosequencing assays

Bisulfite-modified DNA was amplified by PCR in a 50 μl reaction volume, using the primers described in Additional file 4 and reagents supplied by Applied Biosystems. A 40 μl aliquot of each PCR product was used to perform the pyrosequencing reaction following the manufacturer’s protocol and as previously described [26]. Negative controls recommended by the manufacturer were used, as well as positive controls that included (i) DNA in vitro methylated using SssI CpG Methyltransferase (New England Biolabs, Hitchin, UK) following the manufacturer’s instructions, (ii) hypomethylated DNA, generated through PCR and a (iii) mixture of equal volumes of the above methylated and unmethylated controls. The methylation quantification was analysed by Pyro Q-CpG Software (Biotage, Uppsala, Sweden).

### MSI, *APC*, *BRAF*, *KRAS*mutations and CIMP assessment

MSI, *BRAF* and CIMP status of the tumours had been previously reported [14]. Mutational analysis of *APC* [27] and *KRAS* [28] were as previously reported.

### Statistical analysis

A modified version of the *R* package ALL was used to generate image plots of the methylation data within the *R* statistical environment. We used the package *KmL* [29] within the *R* statistical environment [30] to identify the clusters of the trajectories of methylation changes during colorectal neoplastic progression.

Survival analysis was performed using the st functions in Stata 11 [31]. A Cox regression was used to examine the association between survival and average DNA methylation, age, sex, pTMN stage, CIMP and MSI status, and calculate the hazard ratio and the risk of death associated with each variable. The average percentage methylation and age were used as continuous variables and sex, pTMN stage, CIMP and MSI statuses as categorical variables in the Cox regression. The risk of death was first examined by univariable Cox regression and then by multivariable Cox regression to adjust the hazard ratio of one variable in the presence of other variables in the multivariable model. To determine the best predictors of survival a multivariable Cox regression model was constructed based only on the continuous variables plus CIMP and MSI statuses using the stepwise selection method with a *p*(entry) = 0.049 and *p*(removal) = 0.05. Log-rank tests were performed and Kaplan-Meier curves constructed based on the significant variables in the multivariable Cox regression model and used to show the survival patterns of patients depending on the status of these variables. The cut-off used to define high and low methylation for these two variables was based on the literature and previous experience of this type of data. For details of the mixed effects models analysis see (Additional file 5). Where applicable, a Bonferroni adjustment was applied to the p-values from the survival analysis in order to correct for multiple testing.

## Results and discussion

### Increased CpG methylation of multiple Wnt antagonists during colorectal neoplastic progression

**Figure 1:**
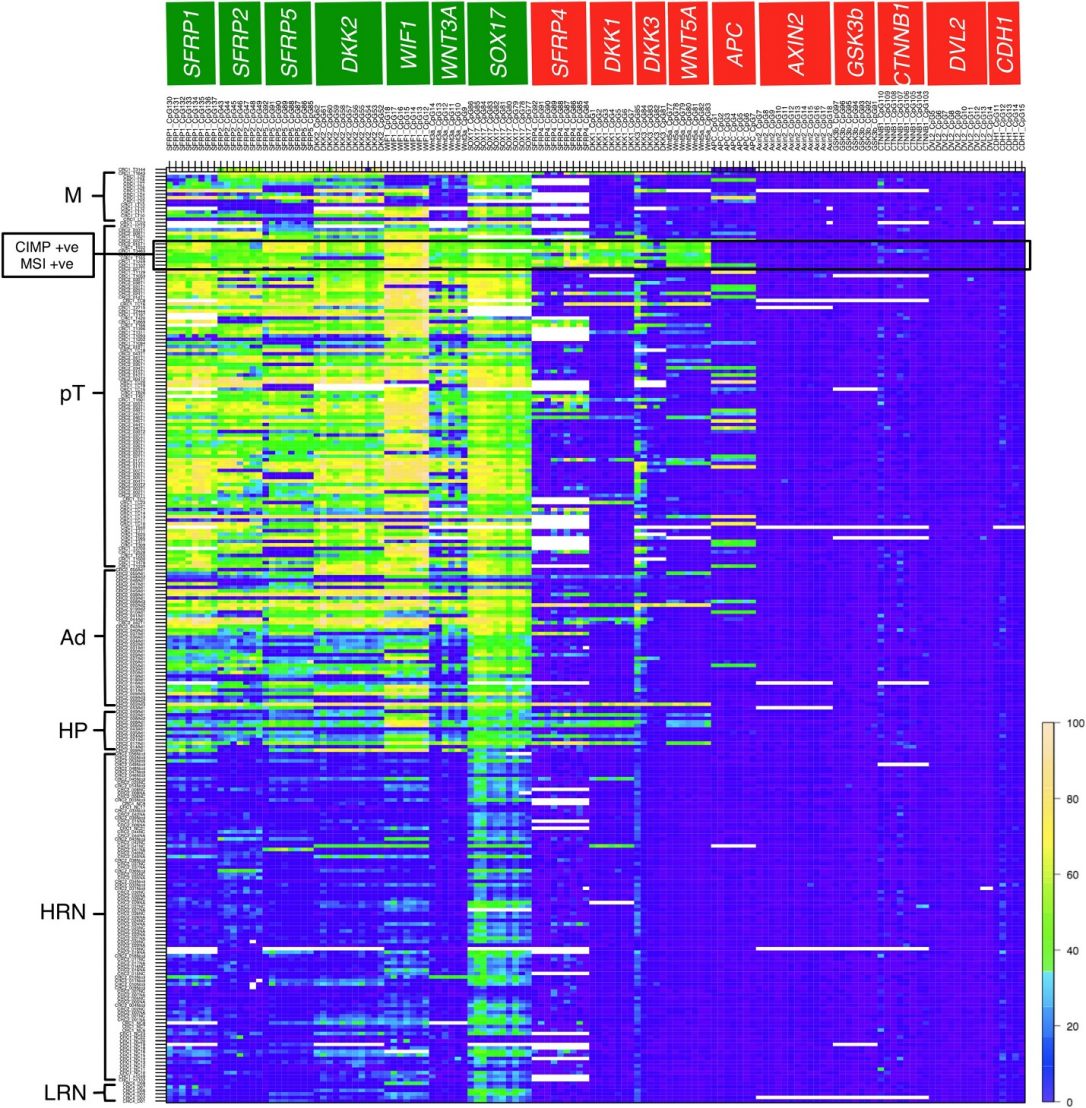
**Image plot showing CpG methylation data from 264 DNA samples as assessed by pyrosequencing.** Samples are grouped by pathological category from normal (bottom) to carcinoma (top). Rows represent individual samples. **LRN**: low-risk normal mucosa from patients with no history of CRC; **HRN**: high-risk normal mucosa from patients with CRC; **HP**: hyperplastic polyps; **Ad**: adenomas; **pT**: primary colorectal adenocarcinomas and **M**: metastatic adenocarcinomas to the liver. Columns show the methylation data for each of the CpG dinucleotides analyzed grouped by gene. A scale shown on the right side of the figure represents the colour spectrum reflecting the percentage of CpG methylation as detected by pyrosequencing. White spaces within the plot indicate missing values due to failure of samples to meet bisulfite conversion or pyrosequencing controls or due to lack of DNA. The subset of CIMP and MSI positive primary colorectal carcinomas is highlighted on the plot.

To gain systematic information regarding the epigenetic silencing of Wnt signalling during CRC progression, we selected a panel of 17 genes encoding Wnt signalling components for pyrosequencing (Additional file 1). We examined the DNA methylation of 134 CpG dinucleotides within promoter-CpG islands associated with these genes (full details of individual CpGs are listed in Additional file 4) in 264 matched normal, adenomatous or hyperplastic polyps and adenocarcinoma tissue samples obtained from 126 patients. As the data represent the different stages and time points along the progression of colorectal neoplasia, we analysed our data by using a k-means clustering algorithm that clusters the trajectories of the matched pyrosequencing data points from the different tissue samples in individual patients based on the Calinski and Harabasz criterion [32]. This criterion revealed two main clusters with significantly different CpG patterns (*P* < 0.05, Fisher’s exact test) (Additional file 6), dependent on whether the genes encode Wnt antagonists or agonists (Figure 1).

The first of these clusters contains seven genes (*SFRP1*, *SFRP2*, *SFRP5*, *DKK2*, *WIF1*, *WNT3A* and *SOX17*) whose CpG methylation increased significantly from normal to adenoma (*P* < 0.001, Wilcoxon signed rank test or paired t-test depending on data distribution and false discovery rate adjusted for multiple testing (Additional file 7)): of these, six encode Wnt antagonists (*SFRP1, SFRP2, SFRP5, DKK2, WIF1* and *SOX17*) with only the Wnt antagonists *SFRP1*, *SFRP2*, *WIF1* and *DKK2* showing further significant increase in methylation from adenoma to carcinoma (*P* < 0.05, Wilcoxon signed rank test or paired t-test depending on data distribution (Additional file 7)). This indicates a strong tendency for Wnt signalling antagonists to become hypermethylated during CRC progression, suggesting that the Wnt signalling levels may increase during the advancement of cancer. Interestingly, mixed effects models analysis of known mutations in three genes (*APC*, *BRAF* and *KRAS)* known to play an important role in colorectal neoplasia showed that most mutations occur at the normal to adenoma transition unlike hypermethylation of Wnt antagonists which continues to accumulate during the adenoma to carcinoma transition (Additional file 8).

Slightly at odds with other members of the first cluster is the presence of *WNT3A* (encoding a Wnt ligand that triggers ‘canonical’ or β-catenin-dependent signalling), [33] which shows the same tendency towards promoter hypermethylation albeit not significantly at the adenoma to the carcinoma stage (*P* = 0.0678, Wilcoxon signed rank test (Additional file 7)). This increase in methylation is somewhat unexpected as it suggests that this canonical Wnt ligand decreases during progression, although we have not shown this explicitly. We note that several other Wnt ligands such as WNT2, WNT10A and WNT6 are expressed at high levels in CRC samples, [34–37] which could substitute for the potentially decreasing Wnt3a in the activation of β-catenin.

The second cluster contains 10 genes (*SFRP4*, *DKK1*, *DKK3*, *WNT5A*, *APC*, *AXIN2*, *GSK3b*, *CTNNB1*, *DVL2*, *CDH1*) whose methylation is less frequent, and at lower levels. However, five of the genes in this cluster exhibit a moderate level of progressive CpG methylation, and thus form a distinct sub-group. This sub-group includes four genes that encode further Wnt antagonists (*SFRP4, DKK1, DKK3, APC*). The fifth gene encodes WNT5A, a ligand that triggers β-catenin-independent (‘non-canonical’) signalling, which can be accompanied by an attenuation of β-catenin-dependent Wnt signalling [38]. The remaining five genes show no detectable promoter methylation, and thus form a second sub-group. This sub-group includes the two genes in our panel that encode positive Wnt signalling components, namely DVL2 and β-catenin. It also contains *AXIN2*, a gene universally activated by β-catenin during Wnt signalling, [39] which is as expected since this gene is strongly expressed during the progression of CRC [11] (see also below).

Interestingly, *APC* was amongst the subset of genes with a considerable tendency for hypermethylation in carcinomas (Figure 1). Hypermethylation of *APC* was present in carcinomas independently of whether or not the tumours already bear *APC* mutations (Additional file 9). Given that the great majority of *APC* mutations in CRC cause APC truncations that retain partial function (e.g. the binding to β-catenin), [40] this suggests that the observed hypermethylation of *APC* could cause epigenetic silencing and reduced expression of the mutant truncated APC. This accounts for a further reduction of *APC* function, beyond the level that caused initiation of tumorigenesis. In other words, epigenetic silencing of *APC* could be equivalent to epigenetic silencing of extracellular Wnt inhibitors, boosting the levels of Wnt signalling activity during CRC progression.

Our evidence supports the hypothesis that the selective and progressive hypermethylation of Wnt antagonists increases Wnt signalling during the progression of colorectal cancer, beyond the initial Wnt hyperactivation caused by the initiating mutations – typically *APC*. An important implication is that Wnt signalling needs to be at least sustained, if not boosted, in order for adenomas to progress to colorectal carcinomas. This reinforces a previous conclusion that Wnt signalling is critical not only for the initiation of CRC, but also for its progression [10]. The presence of Polycomb marks in regulatory regions of genes that are *de novo* methylated in cancer has been proposed to be the mechanism by which certain genes become preferentially hypermethylated in cancer [41–44]. The Wnt antagonists *SFRP1, SFRP2, SFRP4, SFRP5, DKK1, DKK2, SOX17* and *WIF1* have all been reported to be Polycomb target genes in human embryonic stem cells, embryonic fibroblasts, lymphoblasts and murine embryonic stem cells, [45, 46] raising the possibility that Polycomb-induced epigenetic silencing may be the underlying mechanism for the selective hypermethylation of these Wnt antagonists. However, *CTNNB1* and *AXIN2* have also been reported to be Polycomb target genes, [41, 45, 47] but showed no detectable hypermethylation during colorectal neoplastic progression in our sample set, suggesting that factors in addition to the Polycomb determine whether or not the promoter of a Wnt gene becomes epigenetically silenced.

We have shown previously that CIMP correlates with the pattern of global CpG methylation and MSI status in CRC [14]. We have also reported that a small subset of the carcinomas, but none of the normal, hyperplastic polyp nor adenoma samples used in this study, are CIMP or MSI positive, [14] so we asked whether some of the observed hypermethylation correlated with the CIMP and/or MSI status of the corresponding carcinomas. Indeed, several loci showed correlation with CIMP positive state (Additional file 10) but only three the loci (*SFRP4, DKK1* and *WNT5A*) showed significant correlation with both CIMP and MSI positive status (Figure 1; Additional file 10), suggesting that hypermethylation at these three loci may have been exacerbated by the MSI status of these carcinomas and that it may share a common mechanism leading to hypermethylation of the CIMP genes. Importantly though, the three loci also show a tendency for moderate hypermethylation amongst the remaining CIMP- and MSI-negative tumours (Figure 1). This reinforces the notion that these three genes belong to the sub-group of genes with a moderate tendency for hypermethylation during CRC progression.

### Correlation of Wnt antagonist hypermethylation with loss of gene expression

**Figure 2:**
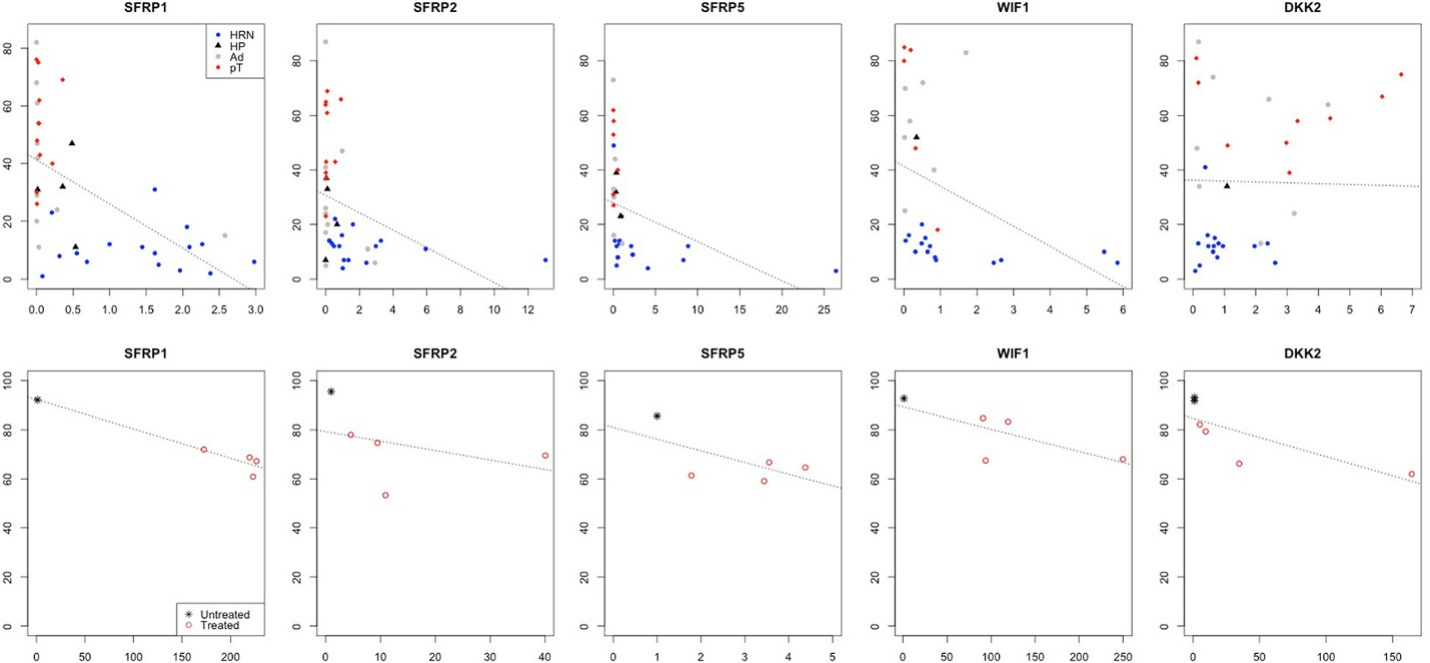
**Shown in the top row are the plots of the correlation between the expression fold-change along the** ***x*** **-axis and the corresponding average levels of methylation percentage along the** ***y*** **-axis for** ***SFRP1, SFRP2, SFRP5, WIF1*** **and** ***DKK2*** **in a subset of tissue samples that included normal (n = 16, HRN), hyperplastic polyps (n = 3, HP), adenomas (n = 10, Ad) and adenocarcinomas (n = 12, pT).** The bottom row shows the corresponding correlations in the CRC cell line HCT116 following 5′-azadeoxycytidine treatment as well as untreated controls. The dotted lines across the plots show the fitted linear model for the data from the corresponding gene. Note that the scale of the *x*-axis varies between the plots depending on the range of the expression fold-change for each of the genes.

To establish whether the observed DNA hypermethylation is functionally relevant, we examined the levels of gene expression of five of the Wnt antagonists (*SFRP1*, *SFRP2*, *SFRP5*, *DKK2* and *WIF1*) with progressive hypermethylation during neoplastic development of CRC. To do this, we used quantitative RT-PCR on total RNA extracted from a subset of matched normal, hyperplastic polyps/adenomas and adenocarcinoma tissue samples and determine the expression fold-change of these five genes. We observed a significant negative correlation between the expression fold-change and the DNA methylation levels for *SFRP1* (*r* = −0.584, *P* < 0.001 Pearson correlation test), *SFRP2* (*r* = −0.340, *P* = 0.032 Pearson correlation test), *SFRP5* (*r* = −0.375, *P* = 0.038 Pearson correlation test) and *WIF1* (*r* = −0.384, *P* = 0.048 Pearson correlation test) (Figure 2). This parallels previous results, which revealed an overall decrease in *SFRP1, SFRP2* and *SFRP5* expression correlating with promoter hypermethylation of these genes [12]. Only *DKK2* (which is expressed at relatively low levels in the normal colorectal mucosa and whose methylation levels are low) showed no significant correlation between methylation and expression levels (*r* = −0.16, *P* = 0.36, Pearson correlation test). Either, our analysis was not sensitive enough to detect such a correlation, or the hypermethylation of *DKK2* is simply a bystander effect, and functionally irrelevant.

To support the observed correlations between DNA methylation and expression levels in the tissue samples, we examined the effect of 5′-aza-2′-deoxycytidine (5-azaDC) treatment on the levels of DNA methylation associated with the same set of five genes (*SFRP1, SFRP2, SFRP5, DKK2* and *WIF1*) in the colorectal cancer cell line HCT-116 (bearing an activating mutation of β-catenin). Untreated cells showed high levels of CpG methylation for each of the five genes, correlating with low levels or absent mRNA expression (Figure 2). However, 5-azaDC treatment not only decreased the levels of methylation, but also increased the corresponding levels of mRNA expression (Figure 2). Thus, the levels of Wnt antagonist expression depended in each case on de-methylation of their CpG islands. This provides strong evidence that the majority of the observed hypermethylation in tumours (Figure 1) is functionally relevant, reducing the expression of the linked genes.

### Hypermethylation of Wnt antagonists correlates with nuclear accumulation of β-catenin

We have previously shown that, in the same set of matched tumour samples examined here for hypermethylation, the levels of nuclear β-catenin increase step-wise from normal tissues to hyperplastic polyps and adenomas to adenocarcinomas [11].

**Figure 3:**
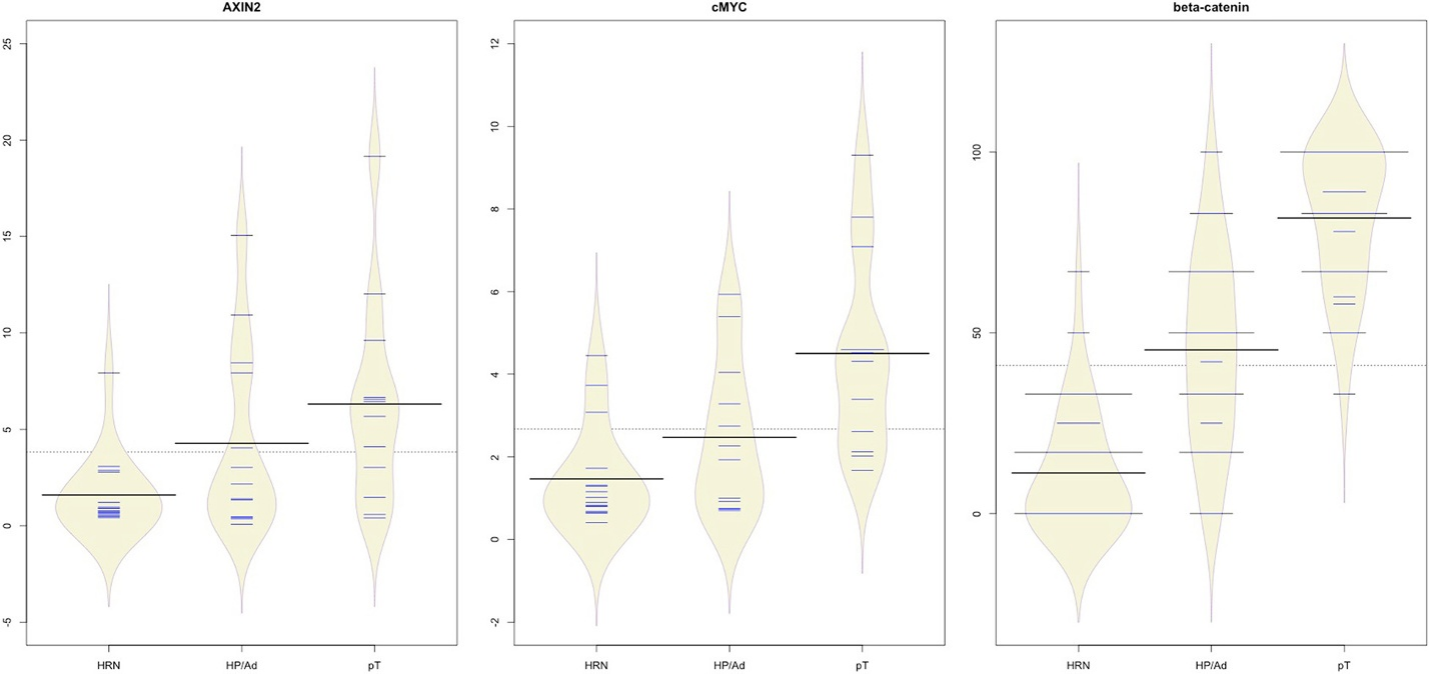
**Bean-plots representing on the** ***y*** **-axis, the relative expression data for the two Wnt downstream targets (** ***AXIN2, cMYC*** **) and the levels of immunohistochemical expression of β-catenin (as described previously [**[11]**]) and along the** ***x*** **-axis the progression pattern by type of tissue (high-risk normal (HRN) mucosa, hyperplastic polyps (HP)/adenomatous tissue (Ad) and primary (pT) adenocarcinoma tissues).** Individual observations are shown as small horizontal lines in a one-dimensional scatter plot. The estimated density of the distributions (visible as a diamond-shaped outline) together with the average for each sample subset (solid horizontal line) and the overall average (solid horizontal line across all samples) are shown. Overall there is progressive increase in the levels of expression of these genes relative to normal tissue. Expression of *AXIN2* and *cMYC* was investigated in 13 cases, which included: normal (n = 16), hyperplastic polyp/adenoma (n = 13) and carcinoma (n = 12) samples.

To confirm the functional relevance of the observed increases in the levels of nuclear β-catenin, we examined the expression levels of two well-established Wnt target genes, *AXIN2* and *c-MYC.* There was a significant increase in their levels of expression during colorectal neoplastic progression (*P* = 0.008 and *P* < 0.001 respectively, Wilcoxon signed rank test) (Figure 3), and for *c-MYC* this significantly and strongly correlated with the levels of nuclear β-catenin (*r* = 0.751, *P* < 0.001, Pearson correlation test). The expression levels of these two genes also correlated significantly with the methylation levels of *SFRP1* (*r* = 0.456, *P* = 0.003 and *r* = 0.494, *P* = 0.001 respectively, Pearson correlation test) and *SFRP2* (*r* = 0.448, *P* = 0.003 and *r* = 0.491, *P* = 0.001 respectively, Pearson correlation test)*.* These results suggest that these SFRPs are the two functionally most relevant Wnt antagonists underlying the mechanism of progressive accumulation of nuclear β-catenin and increased Wnt target gene expression during CRC progression. Their epigenetic silencing could be one of the key factors driving the neoplastic progression in CRC.

### Correlation between hypermethylation of Wnt antagonists and patient survival

To evaluate the association between methylation of the Wnt components and patient survival, we used the average DNA methylation of each gene and analysed them in this study as dichotamous variables using log-rank tests, and as continuous variables in univariable and multivariable Cox regression models. Only the methylation values relative to the adenocarcinoma samples were used. We had survival data available for only 70 patients with a median follow-up time of 59.3 months (range 2–122.3 months) during which 36 patients (51.43%) died. In univariable Cox regression, *DKK1* and *SFRP4* methylation levels had similar hazard ratios of slightly greater than 1, though neither result was significant after adjustment for multiple testing (HR = 1.026 and 1.006 and adjusted *P* = 0.280 and 1.000 respectively), this could be due to the small number of patients included in the analysis. Despite these results, when included in multivariable Cox regression, increase in *DKK1* methylation showed a significant association with poor prognosis (HR = 1.094, *P* = 0.002) whilst increase in *SFRP4* methylation showed a significant association with improved prognosis (HR = 0.942, *P* = 0.017). The directional change of the hazard ratio for *SFRP4* between the univariable and multivariable Cox regression models is attributed to the adjustment for *DKK1* and *WNT5A* in the multivariable model. After adjusting for the hazard of death associated with *DKK1* and *WNT5A*, increases in *SFRP4* methylation appear to be protective rather than hazardous, this unexpected finding could be due to the small sample size analysed and further studies are required to validate these findings. *WNT5A* was included in the multivariable Cox regression model as a covariate of interest but was not found to be significantly associated with survival.

**Figure 4:**
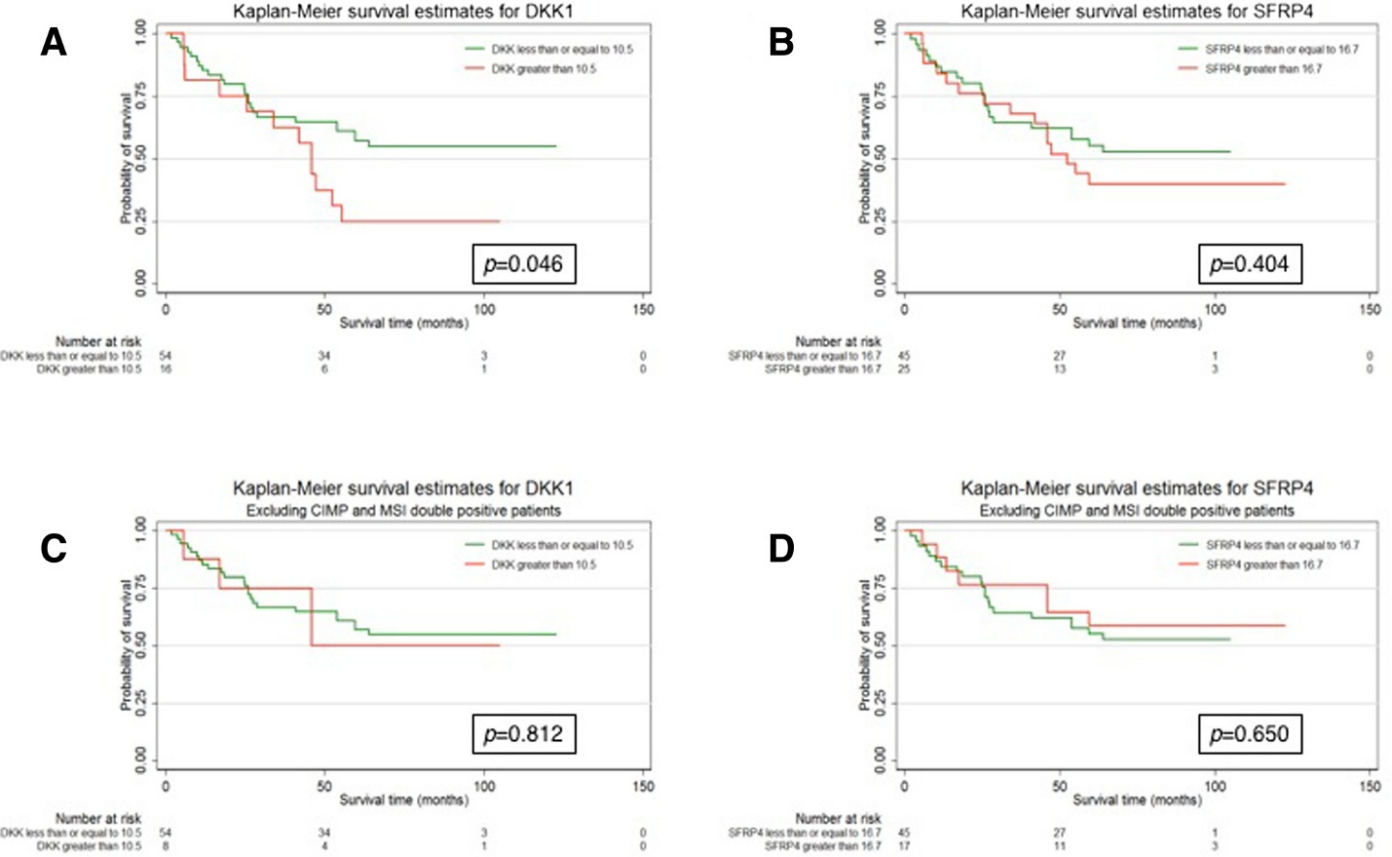
**Kaplan-Meier plots were constructed with methylation level cut-off values of 10.5 for** ***DKK1*** **and 16.7 for** ***SFPR4*** **respectively and log-rank tests were also performed using these cut-offs.** There was a significant difference in the survival pattern of those patients above and below the methylation level cut-off for *DKK1* but not for *SFPR4* (unadjusted log-rank p = 0.046 and 0.404 respectively) as shown in **A** and **B**. With the MSI- and CIMP-positive carcinomas are excluded from this analysis, it remains the case that the hypermethylation of *DKK1* and *SFRP4* was significantly associated with poor survival for *DKK1* and improved survival for *SFRP4* as shown in **C** and **D**. The cut-off values for *SFRP4 =* 16.7 and *DKK1 =* 10.5 were defined as the average adenocarcinoma group methylation level for these genes.

To represent the data for *DKK1* and *SFPR4* graphically, Kaplan-Meier plots were constructed with methylation level cut-off values of 10.5 for *DKK1* and 16.7 for *SFPR4* respectively (Figure 4A and B), and log-rank tests were also performed using these cut-offs. There was a significant difference in the survival pattern of those patients above and below the methylation level cut-off for *DKK1* but not for *SFPR4* (unadjusted log-rank *P* = 0.046 and 0.404 respectively).

Recall that both genes belong to the sub-group of moderately methylated genes, and that both are prone to hypermethylation in MSI- and CIMP-positive carcinomas (Figure 1). Therefore we repeated the survival analysis after excluding the MSI- and CIMP-positive carcinomas and this showed that the hypermethylation of *DKK1* and *SFRP4* was no longer significantly associated with survival *(*(Figure 4C and D), (unadjusted log-rank *P* = 0.812 and 0.650 respectively)). This suggests that the changes in survival patterns associated with hypermethylation of *DKK1* and *SFRP4* are caused by their association with MSI and CIMP status.

## Conclusions

Analysing the promoters of Wnt signalling antagonists in a large matched sample set of various stages of CRC showed that the frequency and levels of hypermethylation increased with neoplastic progression, in a progressive multistep pattern from normal epithelium to adenoma to adenocarcinoma. Therefore, DNA hypermethylation of the Wnt antagonists *SFRP1, SFRP2, SFRP5, DKK2, WIF1* and *SOX17* could provide useful biomarkers for early detection of CRC in screening studies involving DNA methylation, either in stool or plasma samples. Furthermore, two of the Wnt antagonists that are prone to methylation (*DKK1* and *SFRP4*) appear to have prognostic significance, and so it may prove informative to assess their methylation status upon diagnosis of CRC.

## Electronic supplementary material

Additional file 1: **Is a table with the summary of the Wnt signalling components included in our study.** Each gene is included in one of two categories: active antagonist, and non-antagonists that are further subdivided into negative regulators, agonists or WNT coding proteins based on their known roles in Wnt signalling from the literature. (DOCX 114 KB)

Additional file 2: **Is a table containing the clinicopathological characteristics of patients.** (DOCX 173 KB)

Additional file 3: **Is supplementary information of the methods used in the synthesis of complementary DNA (cDNA) by reverse transcription, quantitative PCR (qPCR) and cell treatment with 5-Aza-2’-Deoxycitidine.** (DOCX 109 KB)

Additional file 4: **Is a table with the sequences of all Pyrosequencing primers and PCR conditions used.** (DOCX 25 KB)

Additional file 5: **Is the analysis supplementary document.** (DOCX 58 KB)

Additional file 6: **Is a group of three figures representing the cluster analysis using** ***KmL*** **package (k-means for longitudinal data), (** ***A*** **) Calinski&Harabasz criterion highest score was two.** ***(B)*** Mean trajectories of each cluster, A in red and B in green (0-LRN, 1-HRN, 2-hyperplastic polyps, 3-adenomatous polyps, 4-primary carcinoma, 5-metastatic adenocarcinoma). ***(C)*** Methylation percentages for each CpG island clustered according to Calinski&Harabasz criterion (A in red, B in green). (JPEG 3 MB)

Additional file 7: **Is a table with the results of the Wilcoxon signed rank test or paired t-test depending on data distribution and false discovery rate adjusted for multiple testing.** (DOCX 83 KB)

Additional file 8: **Includes two figures of the estimates from the linear mixed effect model for methylation (A) and logistic mixed effect model for mutations (B) through the neoplastic progression.** All mutations are present by the adenoma stage while methylation gains continue to increase up to the carcinoma stage showing a considerable reduction at the metastasis stage. (JPEG 1 MB)

Additional file 9: **Is a table listing the APC mutations and LOH status of the samples used in this study.** (DOCX 140 KB)

Additional file 10: **Is a table of the results of the point-biserial correlation test** ***r***_***pb***_ **value and** ***p*** **value of the correlation between DNA methylation of each gene and MSI/MSI in primary tumours.** (DOCX 70 KB)
