# Supplementary material for: Boosting Wnt activity during colorectal cancer progression through selective hypermethylation of Wnt signaling antagonists
Source: BMC Cancer. 2014 Nov 29;14:891. doi: 10.1186/1471-2407-14-891 (PMC4265460; doi:10.1186/1471-2407-14-891)
Supplement: Supplementary file 1 — Additional file 1: Is a table with the summary of the Wnt signalling components included in our study. Each gene is included in one of two categories: active antagonist, and non-antagonists that are further subdivided into negative regulators, agonists or WNT coding proteins based on their known roles in Wnt signalling from the literature. (DOCX 114 KB) [file 12885_2014_5079_MOESM1_ESM.docx]

**Additional data file 1**

Summary of the Wnt signalling components included in our study. Each gene is included in one of two categories: active antagonist, and non‑antagonists that are further subdivided into negative regulators, agonists or WNT coding proteins based on their known roles in Wnt signalling from the literature.

| **Gene/ Gene Family** | **Classification** | **Effect on Wnt activity** |
| --- | --- | --- |
| *SFRPs* (*SFRP1*, 2, 4 & 5)* | Active antagonists | SFRP proteins inhibit the WNT-Frizzled receptor by binding competitively to the WNT proteins through the cysteine rich-domain. Alterations in the DNA sequence of the *SFRP* family members are rare and these genes are predominantly regulated by DNA methylation. In CRC, inactivation of these proteins in a background of downstream mutations (APC or beta-catenin) might seem ineffective. However, it has been shown that re-expression of these genes lead to further down-regulation of the pathway (4, 5). |
| *DKKs* (*DKK1*, 2 & 3)^†^ | Active antagonists | The *DKK* family members inhibit Wnt signalling pathway by binding to the LRP5/6 co-receptor inducing its internalization, degradation and prevent its interaction with the WNT-Frizzled complex (6). |
| *SOX17* | Active antagonist | SOX17 antagonizes beta-catenin/TCF-driven transcription by competing with TCF for beta-catenin binding. SOX17 represses CRC cell proliferation (7, 8). |
| *WIF1* | Active antagonist | WIF1 binds directly to WNT proteins inhibiting their interaction with the Frizzled receptors. However, WIF1 does not have the cysteine rich-domain characteristic of the SFRPs but it has a highly conserved unique WIF domain that has been proposed to be responsible for the Wnt binding ability (9, 10). |

| *APC* | Negative regulator | APC is a member of the beta-catenin destruction complex. Down-regulation of APC leads to beta‑catenin cytoplasmatic and nuclear accumulation. Based on the protein structure there are three SAMP repeats (serine-alanina-methionine-proline) that mediate the interaction between APC and Axin proteins. There are also two different sets of amino acid repeats (15aa and 20aa) that recognize beta-catenin and may function differently within the destruction complex. It is frequently mutated in CRC and it is considered as the initial genetic defect in the pathogenesis of familial adenomatous polyposis and ~90% of colorectal neoplasia (11, 12). |
| --- | --- | --- |
| *AXIN2* | Negative regulator | AXIN2 is a member of the Axin family and is part of the beta-catenin destruction complex. AXIN acts as a scaffolding protein for beta-catenin facilitating the phosphorylation of beta-catenin and APC by GSK3B. AXIN binds directly to APC, beta-catenin, GSK3B and DVL. Mutations in *AXIN2* are considered to be the third most common genetic defect in the Wnt signalling pathway (11, 12) |
| *GSK3B* | Negative regulator | GSK3B is also a member of the beta-catenin destruction complex essential for beta-catenin regulation. In the complex, GSK3B binds to Axin and through its active site is able to phosphorylate beta-catenin , marking it for proteosomal degradation. Inhibition of GSK3B is an important mechanism to prevent beta-catenin degradation (12). |
| *CDH1* | Negative regulator | CDH1 is a cell-cell adhesion molecule and plays an important role in the development and maintenance of epithelial tissues. In cancer, silencing of CDH1 leads to an increase in cell mobility implicating *CDH1* in tumour invasion. Therefore *CDH1* is regarded as an invasion suppressor gene. It binds to beta-catenin at cell junctions, competing with APC binding but inhibiting the translocation of beta-catenin to the nucleus (13). This way CDH1 negatively modulates Wnt signalling. |
| *CTNNB1* | Agonist | CTNNB1 is the main effector of the Wnt signalling pathway. A balanced amount of CTNNB1 is necessary for the maintenance of the epithelium and it is also involved in regulation of cell growth and adhesion. CTNNB1 can bind to several factors such as Tcf transcription factors, APC, AXIN or cell adhesion cadherins. In response to Wnt signalling, CTNNB1 accumulates in the cytoplasm and is translocated into the nucleus where it forms a complex with TCF/LEF1 proteins and activates the transcription of downstream targets involved in cell proliferation, differentiation and migration. Activating mutations in *CTNNB1* leads to abnormal activation of the Wnt pathway, these mutations are mutually exclusive with the inactivating *APC* mutations and are the second most common genetic defect of this pathway (11, 14). |
| *DVL2* | Agonist | DVL2 is a member of the Dishevelled family of proteins that mediate Wnt signalling downstream of the Frizzled receptor but upstream of beta-catenin. Although the mechanism of action is not fully understood, one hypothesis for the positive effect of Dvl proteins in Wnt activation is that, upon WNT-Frizzled binding, Dvl is translocated to the membrane, where it binds to the cytoplasmic portion of Frizzled recruiting AXIN (11, 12). This way AXIN is no longer available to be part of the destruction complex leading to accumulation of beta-catenin and the activation of the Wnt downstream targets transcription. |
| *WNT3A* | WNT coding proteins | *WNT3A* binds to the Frizzled receptor and activates downstream abnormal Wnt signally in cell line experiments. However, expression of *WNT3A* in the intestine has not been found and no mechanisms of gene regulation have been described. |
| *WNT5A* | WNT coding proteins | WNT5A plays a role in canonical and non-canonical Wnt pathways and has tumour promoting and tumour inhibiting effects depending on the cellular context. It is expressed in normal colorectal mucosa and frequently found hypermethylated in CRC cell lines and primary tumours, suggesting a role as tumour suppressor in CRC (15). |

1. Hirata H, Hinoda Y, Ueno K, Majid S, Saini S, Dahiya R (2010) Role of secreted frizzled-related protein 3 in human renal cell carcinoma. *Cancer Res* 70:1896–1905.

2. Ekstrom EJ, Sherwood V, Andersson T (2011) Methylation and loss of Secreted Frizzled-Related Protein 3 enhances melanoma cell migration and invasion. *PLoS ONE* 6:e18674.

3. van Dekken H, Wink JC, Vissers KJ, Franken PF, Ruud Schouten W, J Hop WC, Kuipers EJ, Fodde R, Janneke van der Woude C (2007) Wnt pathway-related gene expression during malignant progression in ulcerative colitis. *Acta Histochem* 109:266–272.

4. Polakis P (2007) The many ways of Wnt in cancer. *Curr Opin Genet Dev* 17:45–51.

5. Suzuki H, Watkins DN, Jair KW, Schuebel KE, Markowitz SD, Chen WD, Pretlow TP, Yang B, Akiyama Y, Van Engeland M *et al.* (2004) Epigenetic inactivation of SFRP genes allows constitutive WNT signaling in colorectal cancer. *Nat Genet* 36:417–422.

6. Schneikert J, Behrens J (2007) The canonical Wnt signalling pathway and its APC partner in colon cancer development. *Gut* 56:417–425.

7. Sinner D, Kordich JJ, Spence JR, Opoka R, Rankin S, Lin SC, Jonatan D, Zorn AM, Wells JM (2007) Sox17 and Sox4 differentially regulate beta-catenin/T-cell factor activity and proliferation of colon carcinoma cells. *Mol Cell Biol* 27:7802–7815.

8. Zhang W, Glockner SC, Guo M, Machida EO, Wang DH, Easwaran H, Van Neste L, Herman JG, Schuebel KE, Watkins DN *et al.* (2008) Epigenetic inactivation of the canonical Wnt antagonist SRY-box containing gene 17 in colorectal cancer. *Cancer Res* 68:2764–2772.

9. Taniguchi H, Yamamoto H, Hirata T, Miyamoto N, Oki M, Nosho K, Adachi Y, Endo T, Imai K, Shinomura Y (2005) Frequent epigenetic inactivation of Wnt inhibitory factor-1 in human gastrointestinal cancers. *Oncogene* 24:7946–7952.

10. Patthy L (2000) The WIF module. *Trends Biochem Sci* 25:12–13.

11. Polakis P (2000) Wnt signaling and cancer. *Genes Dev* 14:1837–1851.

12. Kimelman D, Xu W (2006) beta-catenin destruction complex: insights and questions from a structural perspective. *Oncogene* 25:7482–7491.

13. Kuphal F, Behrens J (2006) E-cadherin modulates Wnt-dependent transcription in colorectal cancer cells but does not alter Wnt-independent gene expression in fibroblasts. *Exp Cell Res* 312:457–467.

14. Ying Y, Tao Q (2009) Epigenetic disruption of the WNT/beta-catenin signaling pathway in human cancers. *Epigenetics* 4:307–312.

15. Ying J, Li H, Yu J, Ng KM, Poon FF, Wong SC, Chan AT, Sung JJ, Tao Q (2008) WNT5A exhibits tumor-suppressive activity through antagonizing the Wnt/beta-catenin signaling, and is frequently methylated in colorectal cancer. *Clin Cancer Res* 14:55–61.
