# Supplementary material for: Boosting Wnt activity during colorectal cancer progression through selective hypermethylation of Wnt signaling antagonists
Source: BMC Cancer. 2014 Nov 29;14:891. doi: 10.1186/1471-2407-14-891 (PMC4265460; doi:10.1186/1471-2407-14-891)
Supplement: Supplementary file 2 — Additional file 2: Is a table containing the clinicopathological characteristics of patients. (DOCX 173 KB) [file 12885_2014_5079_MOESM2_ESM.docx]

**Additional data file 2 – Clinicopathological Characteristics of Patients**

| **ID** | **CIMP Score** | **MSI Status** | **Age** | **T** | **N** | **M** | **Sex** | **Side** | **Study Number** | **Tissue** |
| --- | --- | --- | --- | --- | --- | --- | --- | --- | --- | --- |
| CRC4_ND1 | NA | NA | NA | NA | NA | NA | NA | NA | CRC1 | LRN |
| CRC4_ND2 | NA | NA | NA | NA | NA | NA | NA | NA | CRC1 | LRN |
| CRC4_ND5 | NA | NA | NA | NA | NA | NA | NA | NA | CRC1 | LRN |
| CRC4_ND6 | NA | NA | NA | NA | NA | NA | NA | NA | CRC1 | LRN |
| CRC4_ND7 | NA | NA | NA | NA | NA | NA | NA | NA | CRC1 | LRN |
| CRC4_ND8 | NA | NA | NA | NA | NA | NA | NA | NA | CRC1 | LRN |
| CRC1_n1102a | 0 | 0 | NA | NA | NA | pMx | NA | NA | CRC1 | HRN |
| CRC1_N1249A | 0 | 0 | NA | NA | NA | pMx | NA | NA | CRC1 | HRN |
| CRC1_NC10 | 0 | 0 | 82 | 3 | 2 | pM1 | NA | NA | CRC1 | HRN |
| CRC1_NC11 | 0 | 0 | 58 | 3 | 2 | pM1 | MALE | NA | CRC1 | HRN |
| CRC1_NC12 | 0 | 0 | 74 | 3 | 2 | pM1 | MALE | NA | CRC1 | HRN |
| CRC1_NC13 | 0 | 0 | 93 | 4 | 0 | pM1 | MALE | NA | CRC1 | HRN |
| CRC1_NC14 | 0 | 0 | 56 | 4 | 0 | pM1 | FEMALE | NA | CRC1 | HRN |
| CRC1_NC15 | 0 | 0 | 84 | 3 | 2 | pM1 | FEMALE | NA | CRC1 | HRN |
| CRC1_NC16 | 0 | 0 | 91 | 4 | 2 | pM1 | FEMALE | NA | CRC1 | HRN |
| CRC1_NC18 | 0 | 0 | 73 | NA | NA | pMx | FEMALE | NA | CRC1 | HRN |
| CRC1_NC19 | 0 | 0 | 78 | 3 | 1 | pM1 | MALE | NA | CRC1 | HRN |
| CRC1_NC20 | 0 | 0 | 59 | 3 | 1 | pM1 | MALE | NA | CRC1 | HRN |
| CRC1_NC21 | 0 | NA | 69 | 4 | 1 | pM1 | FEMALE | NA | CRC1 | HRN |
| CRC1_NC22 | 0 | 0 | 64 | 3 | 2 | pM1 | FEMALE | NA | CRC1 | HRN |
| CRC1_NC23 | 0 | 0 | 59 | 3 | 2 | pM1 | MALE | NA | CRC1 | HRN |
| CRC1_NC6 | 0 | 0 | 66 | 3 | 1 | pM1 | MALE | NA | CRC1 | HRN |
| CRC1_NC7 | 0 | 0 | 81 | 3 | 0 | pM0 | MALE | NA | CRC1 | HRN |
| CRC1_NC8 | 0 | 0 | 73 | 4 | 1 | pM1 | FEMALE | NA | CRC1 | HRN |
| CRC2_001_NA | 1 | 0 | 86 | 4 | 2 | pMx | MALE | R | CRC2 | HRN |
| CRC2_001_NC | 1 | 0 | 86 | 4 | 2 | pMx | MALE | R | CRC2 | HRN |
| CRC2_002_NA | 0 | 0 | 75 | 3 | 1 | pMx | MALE | L | CRC2 | HRN |
| CRC2_002_NC | 0 | 0 | 75 | 3 | 1 | pMx | MALE | L | CRC2 | HRN |
| CRC2_004_Ncol | 1 | 0 | 84 | 3 | 0 | pMx | MALE | R | CRC2 | HRN |
| CRC2_005_NA | 0 | 0 | 68 | 2 | 0 | pMx | MALE | L | CRC2 | HRN |
| CRC2_005_NC | 0 | 0 | 68 | 2 | 0 | pMx | MALE | L | CRC2 | HRN |
| CRC2_006_NA | 0 | NA | 79 | 4 | 2 | pMx | FEMALE | R | CRC2 | HRN |
| CRC2_007_NA | 0 | 0 | 32 | 3 | 1 | pMx | MALE | L | CRC2 | HRN |
| CRC2_007_NC | 0 | 0 | 32 | 3 | 1 | pMx | MALE | L | CRC2 | HRN |
| CRC2_009_Ncol | 0 | 0 | 66 | 2 | 0 | pMx | FEMALE | L | CRC2 | HRN |
| CRC2_011_Ncol | 0 | 0 | 93 | 4 | 0 | pM1 | FEMALE | R | CRC2 | HRN |
| CRC2_012_Ncol | 1 | 0 | 73 | 3 | 2 | pMx | MALE | L | CRC2 | HRN |
| CRC2_013_Ncol | 1 | 0 | 88 | 3 | 0 | pMx | FEMALE | R | CRC2 | HRN |
| CRC2_015_NA | 0 | NA | 48 | 2 | 0 | pMx | FEMALE | L | CRC2 | HRN |
| CRC2_015_NC | 0 | 0 | 48 | 2 | 0 | pMx | FEMALE | L | CRC2 | HRN |
| CRC2_016_NA | 0 | 0 | 57 | 3 | 0 | pMx | MALE | NA | CRC2 | HRN |
| CRC2_016_NC | 1 | 0 | 57 | 3 | 0 | pMx | MALE | NA | CRC2 | HRN |
| CRC2_017_NA | 0 | 0 | 79 | 3 | 1 | pMx | MALE | L | CRC2 | HRN |
| CRC2_017_NC | 0 | 0 | 79 | 3 | 1 | pMx | MALE | L | CRC2 | HRN |
| CRC2_018_Ncol | 0 | 0 | 69 | 3 | 2 | pMx | MALE | L | CRC2 | HRN |
| CRC2_019_NA | 0 | 0 | 92 | NA | NA | pMx | FEMALE | L | CRC2 | HRN |
| CRC2_019_NC | 0 | 0 | 92 | NA | NA | pMx | FEMALE | L | CRC2 | HRN |
| CRC2_020_NA | 0 | 0 | 81 | 3 | 2 | pMx | MALE | L | CRC2 | HRN |
| CRC2_020_NC | 0 | 0 | 81 | 3 | 2 | pMx | MALE | L | CRC2 | HRN |
| CRC2_021_NA | 0 | 0 | 74 | 3 | 1 | pMx | FEMALE | L | CRC2 | HRN |
| CRC2_021_NC | 0 | 0 | 74 | 3 | 1 | pMx | FEMALE | L | CRC2 | HRN |
| CRC2_022_NA | 1 | 0 | 74 | 3 | 1 | pMx | FEMALE | R | CRC2 | HRN |
| CRC2_022_NC | 0 | 0 | 74 | 3 | 1 | pMx | FEMALE | R | CRC2 | HRN |
| CRC2_023_NA | 0 | 0 | 79 | 2 | 0 | pMx | MALE | L | CRC2 | HRN |
| CRC2_023_NC | 1 | 0 | 79 | 2 | 0 | pMx | MALE | L | CRC2 | HRN |
| CRC2_024_NA | 0 | 0 | 74 | 2 | 0 | pMx | MALE | L | CRC2 | HRN |
| CRC2_024_NC | 0 | 0 | 74 | 2 | 0 | pMx | MALE | L | CRC2 | HRN |
| CRC2_026_NA | 0 | 0 | 80 | 2 | 0 | pMx | MALE | L | CRC2 | HRN |
| CRC2_026_NC | 0 | 0 | 80 | 2 | 0 | pMx | MALE | L | CRC2 | HRN |
| CRC2_027_NA | 0 | 0 | 81 | 3 | 1 | pMx | FEMALE | L | CRC2 | HRN |
| CRC2_027_NC | 0 | 0 | 81 | 3 | 1 | pMx | FEMALE | L | CRC2 | HRN |
| CRC2_029_NA | 1 | 0 | 74 | 3 | 0 | pMx | MALE | L | CRC2 | HRN |
| CRC2_029_NC | 0 | 0 | 74 | 3 | 0 | pMx | MALE | L | CRC2 | HRN |
| CRC2_030_NA | 0 | 0 | 52 | 3 | 2 | pMx | MALE | L | CRC2 | HRN |
| CRC2_030_NC | 0 | 0 | 52 | 3 | 2 | pMx | MALE | L | CRC2 | HRN |
| CRC2_031_Ncol | 0 | 0 | 70 | 3 | 1 | pMx | MALE | L | CRC2 | HRN |
| CRC2_032_Ncol | 0 | 0 | 83 | 3 | 0 | pMx | MALE | L | CRC2 | HRN |
| CRC2_033_Ncol | 1 | 0 | 84 | 3 | 0 | pMx | MALE | L | CRC2 | HRN |
| CRC2_034_Ncol | 0 | 0 | 82 | 3 | 0 | pMx | MALE | L | CRC2 | HRN |
| CRC2_035_NA | 1 | 0 | 57 | 4 | 1 | pMx | MALE | L | CRC2 | HRN |
| CRC2_035_NC | 1 | 0 | 57 | 4 | 1 | pMx | MALE | L | CRC2 | HRN |
| CRC2_036_Ncol | 3 | 0 | 86 | 3 | 1 | pMx | MALE | R | CRC2 | HRN |
| CRC2_037_NA | 0 | 0 | 32 | 2 | 0 | pMx | MALE | L | CRC2 | HRN |
| CRC2_037_NC | 0 | 0 | 32 | 2 | 0 | pMx | MALE | L | CRC2 | HRN |
| CRC2_038_Ncol | 0 | 0 | 76 | 3 | 1 | pMx | MALE | L | CRC2 | HRN |
| CRC2_039_Ncol | 0 | NA | 64 | 3 | 2 | pMx | MALE | L | CRC2 | HRN |
| CRC2_040_NA | 0 | 0 | 73 | 3 | 0 | pMx | MALE | R | CRC2 | HRN |
| CRC2_040_NC | 0 | 0 | 73 | 3 | 0 | pMx | MALE | R | CRC2 | HRN |
| CRC2_041_NA | 0 | 0 | 77 | 3 | 0 | pMx | MALE | L | CRC2 | HRN |
| CRC2_041_NC | 0 | 0 | 77 | 3 | 0 | pMx | MALE | L | CRC2 | HRN |
| CRC2_042_NA | 0 | 0 | 67 | NA | NA | pMx | MALE | T | CRC2 | HRN |
| CRC2_042_NC | 0 | 0 | 67 | NA | NA | pMx | MALE | T | CRC2 | HRN |
| CRC2_043_Ncol | 3 | 0 | 69 | 2 | 0 | pMx | MALE | L | CRC2 | HRN |
| CRC2_044_NA | 0 | 0 | 81 | 3 | 0 | pMx | MALE | L | CRC2 | HRN |
| CRC2_044_NC | 1 | 0 | 81 | 3 | 0 | pMx | MALE | L | CRC2 | HRN |
| CRC2_045_Ncol | 0 | 0 | NA | NA | NA | pMx | NA | NA | CRC2 | HRN |
| CRC2_046_Ncol | 0 | 0 | NA | NA | NA | pMx | NA | NA | CRC2 | HRN |
| CRC2_047_Ncol | 0 | 0 | NA | NA | NA | pMx | NA | NA | CRC2 | HRN |
| CRC2_048_Ncol | 0 | 0 | NA | NA | NA | pMx | NA | NA | CRC2 | HRN |
| CRC2_049_Ncol | 0 | 0 | NA | NA | NA | pMx | NA | NA | CRC2 | HRN |
| CRC2_053_Ncol | 0 | 0 | NA | NA | NA | pMx | NA | NA | CRC2 | HRN |
| CRC2_055_Ncol | 0 | 0 | NA | NA | NA | pMx | NA | NA | CRC2 | HRN |
| CRC2_056_Ncol | 0 | 0 | NA | NA | NA | pMx | NA | NA | CRC2 | HRN |
| CRC2_010_Ncol | 0 | 0 | 79 | 3 | 1 | pMx | FEMALE | R | CRC2 | HRN |
| CRC2_009_HP | 0 | 0 | 66 | 2 | 0 | pMx | FEMALE | L | CRC2 | HP |
| CRC2_014_HP | 1 | 0 | 83 | 3 | 1 | pMx | MALE | L | CRC2 | HP |
| CRC2_017_HP | 3 | 0 | 79 | 3 | 1 | pMx | MALE | L | CRC2 | HP |
| CRC2_021_HP | 2 | 0 | 74 | 3 | 1 | pMx | FEMALE | L | CRC2 | HP |
| CRC2_024_HP | 2 | 0 | 74 | 2 | 0 | pMx | MALE | L | CRC2 | HP |
| CRC2_035_HP | 2 | 0 | 57 | 4 | 1 | pMx | MALE | L | CRC2 | HP |
| CRC2_043_HP | 0 | 50 | 69 | 2 | 0 | pMx | MALE | L | CRC2 | HP |
| CRC2_002_Ad3 | 3 | 0 | 75 | 3 | 1 | pMx | MALE | L | CRC2 | Ad |
| CRC2_009_Ad2 | 1 | 0 | 66 | 2 | 0 | pMx | FEMALE | L | CRC2 | Ad |
| CRC2_009_Ad3 | 0 | 0 | 66 | 2 | 0 | pMx | FEMALE | L | CRC2 | Ad |
| CRC2_009_Ad5 | 2 | 0 | 66 | 2 | 0 | pMx | FEMALE | L | CRC2 | Ad |
| CRC2_010_Ad1 | 0 | NA | 79 | 3 | 1 | pMx | FEMALE | R | CRC2 | Ad |
| CRC2_011_Ad1 | 1 | 0 | 93 | 4 | 0 | pM1 | FEMALE | R | CRC2 | Ad |
| CRC2_012_Ad1 | 1 | 0 | 73 | 3 | 2 | pMx | MALE | L | CRC2 | Ad |
| CRC2_013_Ad1 | 2 | 0 | 88 | 3 | 0 | pMx | FEMALE | R | CRC2 | Ad |
| CRC2_016_Ad1 | 1 | 0 | 57 | 3 | 0 | pMx | MALE | NA | CRC2 | Ad |
| CRC2_018_Ad1 | 1 | 0 | 69 | 3 | 2 | pMx | MALE | L | CRC2 | Ad |
| CRC2_019_Ad1 | 1 | 0 | 92 | NA | NA | pMx | FEMALE | L | CRC2 | Ad |
| CRC2_020_Ad1 | 2 | 0 | 81 | 3 | 2 | pMx | MALE | L | CRC2 | Ad |
| CRC2_023_Ad1 | 2 | 0 | 79 | 2 | 0 | pMx | MALE | L | CRC2 | Ad |
| CRC2_025_Ad1 | 2 | 0 | 64 | 3 | 0 | pMx | MALE | L | CRC2 | Ad |
| CRC2_026_Ad1 | 2 | 0 | 80 | 2 | 0 | pMx | MALE | L | CRC2 | Ad |
| CRC2_027_Ad1 | 1 | 0 | 81 | 3 | 1 | pMx | FEMALE | L | CRC2 | Ad |
| CRC2_029_Ad1 | 2 | 0 | 74 | 3 | 0 | pMx | MALE | L | CRC2 | Ad |
| CRC2_030_Ad1 | 2 | 0 | 52 | 3 | 2 | pMx | MALE | L | CRC2 | Ad |
| CRC2_031_Ad1 | 1 | 0 | 70 | 3 | 1 | pMx | MALE | L | CRC2 | Ad |
| CRC2_032_Ad1 | 2 | 0 | 83 | 3 | 0 | pMx | MALE | L | CRC2 | Ad |
| CRC2_034_Ad1 | 2 | 0 | 82 | 3 | 0 | pMx | MALE | L | CRC2 | Ad |
| CRC2_036_Ad1 | 2 | 0 | 86 | 3 | 1 | pMx | MALE | R | CRC2 | Ad |
| CRC2_037_Ad1 | 2 | 0 | 32 | 2 | 0 | pMx | MALE | L | CRC2 | Ad |
| CRC2_040_Ad1 | 2 | 0 | 73 | 3 | 0 | pMx | MALE | R | CRC2 | Ad |
| CRC2_041_Ad1 | 1 | 50 | 77 | 3 | 0 | pMx | MALE | L | CRC2 | Ad |
| CRC2_042_Ad1 | 2 | 0 | 67 | NA | NA | pMx | MALE | T | CRC2 | Ad |
| CRC2_044_Ad1 | 1 | 0 | 81 | 3 | 0 | pMx | MALE | L | CRC2 | Ad |
| CRC2_045_Ad1 | 0 | 0 | NA | NA | NA | pMx | NA | NA | CRC2 | Ad |
| CRC2_046_Ad1 | 0 | 0 | NA | NA | NA | pMx | NA | NA | CRC2 | Ad |
| CRC2_047_Ad1 | 0 | 0 | NA | NA | NA | pMx | NA | NA | CRC2 | Ad |
| CRC2_048_Ad1 | 0 | 0 | NA | NA | NA | pMx | NA | NA | CRC2 | Ad |
| CRC2_048_Ad2 | 0 | 0 | NA | NA | NA | pMx | NA | NA | CRC2 | Ad |
| CRC2_049_Ad1 | 0 | 0 | NA | NA | NA | pMx | NA | NA | CRC2 | Ad |
| CRC2_053_Ad1 | 0 | 0 | NA | NA | NA | pMx | NA | NA | CRC2 | Ad |
| CRC2_055_Ad1 | 0 | 0 | NA | NA | NA | pMx | NA | NA | CRC2 | Ad |
| CRC2_056_Ad | 0 | 0 | NA | NA | NA | pMx | NA | NA | CRC2 | Ad |
| CRC1_T1053B | 3 | 50 | 54 | 3 | 0 | pM0 | MALE | NA | CRC1 | pT |
| CRC1_T1102A | 6 | 100 | 74 | 3 | 1 | pM0 | MALE | NA | CRC1 | pT |
| CRC1_T1129A | 3 | 50 | 80 | 3 | 0 | pM0 | MALE | NA | CRC1 | pT |
| CRC1_T1197A | 3 | 0 | 71 | 4 | 3 | pM0 | MALE | NA | CRC1 | pT |
| CRC1_T1239A | 1 | 0 | 88 | 2 | 0 | pM0 | MALE | NA | CRC1 | pT |
| CRC1_T1249A | 6 | 100 | 76 | 3 | 0 | pM0 | FEMALE | NA | CRC1 | pT |
| CRC1_T1311B | 3 | NA | 73 | 3 | 1 | pM1 | MALE | NA | CRC1 | pT |
| CRC1_T1479A | 1 | 0 | 75 | 3 | 2 | pM0 | MALE | NA | CRC1 | pT |
| CRC1_T1500B | 4 | 0 | 81 | 3 | 2 | pM1 | FEMALE | NA | CRC1 | pT |
| CRC1_T1501A | 3 | 50 | 62 | 3 | 1 | pM0 | FEMALE | NA | CRC1 | pT |
| CRC1_T1621B | 3 | NA | 71 | 3 | 0 | pM0 | FEMALE | NA | CRC1 | pT |
| CRC1_T165A | 6 | 100 | 77 | 3 | 0 | pM0 | FEMALE | NA | CRC1 | pT |
| CRC1_T168A | 2 | 0 | 68 | 3 | 1 | pM1 | MALE | NA | CRC1 | pT |
| CRC1_T220A | 0 | 0 | 64 | 3 | 1 | pM1 | MALE | NA | CRC1 | pT |
| CRC1_T2444A | 2 | 0 | 52 | 2 | 0 | pM0 | FEMALE | NA | CRC1 | pT |
| CRC1_T2528C | 2 | 0 | 52 | 3 | 2 | pM1 | FEMALE | NA | CRC1 | pT |
| CRC1_T2688A | 5 | 100 | 82 | 2 | 0 | pM0 | MALE | NA | CRC1 | pT |
| CRC1_T2700A | 2 | 0 | 64 | NA | NA | pMx | NA | NA | CRC1 | pT |
| CRC1_T2715B | 3 | 0 | 63 | 4 | 2 | pM1 | MALE | NA | CRC1 | pT |
| CRC1_T309A | 2 | 0 | 63 | 1 | 0 | pM0 | MALE | NA | CRC1 | pT |
| CRC1_T3463A | 6 | 100 | 66 | 3 | 0 | pM0 | FEMALE | NA | CRC1 | pT |
| CRC1_T377A | 2 | 0 | 59 | 3 | 1 | pM1 | FEMALE | NA | CRC1 | pT |
| CRC1_T402B | 1 | 0 | 43 | 3 | 0 | pM0 | FEMALE | NA | CRC1 | pT |
| CRC1_T451A | 2 | 50 | 71 | 4 | 2 | pM0 | FEMALE | NA | CRC1 | pT |
| CRC1_T565A | 2 | 0 | 63 | NA | 0 | pM0 | FEMALE | NA | CRC1 | pT |
| CRC1_T625A | 2 | 0 | 53 | 3 | 1 | pM0 | MALE | NA | CRC1 | pT |
| CRC1_T771A | 1 | 0 | 92 | 3 | 1 | pM0 | FEMALE | NA | CRC1 | pT |
| CRC1_T826A | 1 | 50 | 78 | 3 | 0 | pM0 | FEMALE | NA | CRC1 | pT |
| CRC1_T932B | 6 | 100 | 71 | 4 | 2 | pM0 | FEMALE | NA | CRC1 | pT |
| CRC1_T940A | 2 | 0 | 66 | 2 | 0 | pM0 | MALE | NA | CRC1 | pT |
| CRC1_TC10 | 2 | 0 | 82 | 3 | 2 | pM1 | NA | NA | CRC1 | pT |
| CRC1_TC11 | 0 | 0 | 58 | 3 | 2 | pM1 | MALE | NA | CRC1 | pT |
| CRC1_TC12 | 0 | 0 | 74 | 3 | 2 | pM1 | MALE | NA | CRC1 | pT |
| CRC1_TC13 | 2 | 0 | 93 | 4 | 0 | pM1 | MALE | NA | CRC1 | pT |
| CRC1_TC14 | 0 | 0 | 56 | 4 | 0 | pM1 | FEMALE | NA | CRC1 | pT |
| CRC1_TC15 | 2 | 50 | 84 | 3 | 2 | pM1 | FEMALE | NA | CRC1 | pT |
| CRC1_TC16 | 6 | 0 | 91 | 4 | 2 | pM1 | FEMALE | NA | CRC1 | pT |
| CRC1_TC17 | 2 | 0 | 71 | 3 | 2 | pM1 | FEMALE | NA | CRC1 | pT |
| CRC1_TC18 | 1 | 100 | 73 | NA | NA | pMx | FEMALE | NA | CRC1 | pT |
| CRC1_TC19 | 1 | 50 | 78 | 3 | 1 | pM1 | MALE | NA | CRC1 | pT |
| CRC1_TC20 | 1 | 50 | 59 | 3 | 1 | pM1 | MALE | NA | CRC1 | pT |
| CRC1_TC21 | 3 | NA | 69 | 4 | 1 | pM1 | FEMALE | NA | CRC1 | pT |
| CRC1_TC23 | 2 | 0 | 59 | 3 | 2 | pM1 | MALE | NA | CRC1 | pT |
| CRC1_TC7 | 2 | 0 | 81 | 3 | 0 | pM0 | MALE | NA | CRC1 | pT |
| CRC1_TC8 | 3 | 0 | 73 | 4 | 1 | pM1 | FEMALE | NA | CRC1 | pT |
| CRC2_001_T1 | 1 | 0 | 86 | 4 | 2 | pMx | MALE | R | CRC2 | pT |
| CRC2_002_T1 | 1 | 0 | 75 | 3 | 1 | pMx | MALE | L | CRC2 | pT |
| CRC2_003_T1 | 1 | 0 | 76 | 3 | 0 | pMx | FEMALE | L | CRC2 | pT |
| CRC2_004_T1 | 2 | 0 | 84 | 3 | 0 | pMx | MALE | R | CRC2 | pT |
| CRC2_004_T2 | 2 | 50 | 84 | 3 | 0 | pMx | MALE | R | CRC2 | pT |
| CRC2_010_T1 | 3 | 50 | 79 | 3 | 1 | pMx | FEMALE | R | CRC2 | pT |
| CRC2_011_T1 | 2 | 0 | 93 | 4 | 0 | pM1 | FEMALE | R | CRC2 | pT |
| CRC2_012_T1 | 0 | 0 | 73 | 3 | 2 | pMx | MALE | L | CRC2 | pT |
| CRC2_013_T1 | 2 | 0 | 88 | 3 | 0 | pMx | FEMALE | R | CRC2 | pT |
| CRC2_014_T1 | 2 | 0 | 83 | 3 | 1 | pMx | MALE | L | CRC2 | pT |
| CRC2_015_T1 | 2 | 50 | 48 | 2 | 0 | pMx | FEMALE | L | CRC2 | pT |
| CRC2_016_T1 | 6 | 100 | 57 | 3 | 0 | pMx | MALE | NA | CRC2 | pT |
| CRC2_017_T1 | 2 | 0 | 79 | 3 | 1 | pMx | MALE | L | CRC2 | pT |
| CRC2_018_T1 | 3 | 100 | 69 | 3 | 2 | pMx | MALE | L | CRC2 | pT |
| CRC2_020_T1 | 2 | 0 | 81 | 3 | 2 | pMx | MALE | L | CRC2 | pT |
| CRC2_021_T1 | 2 | 0 | 74 | 3 | 1 | pMx | FEMALE | L | CRC2 | pT |
| CRC2_022_T1 | 4 | 100 | 74 | 3 | 1 | pMx | FEMALE | R | CRC2 | pT |
| CRC2_023_T1 | 2 | NA | 79 | 2 | 0 | pMx | MALE | L | CRC2 | pT |
| CRC2_024_T1 | 5 | 0 | 74 | 2 | 0 | pMx | MALE | L | CRC2 | pT |
| CRC2_025_T1 | 1 | 0 | 64 | 3 | 0 | pMx | MALE | L | CRC2 | pT |
| CRC2_026_T1 | 2 | 0 | 80 | 2 | 0 | pMx | MALE | L | CRC2 | pT |
| CRC2_027_T1 | 3 | 50 | 81 | 3 | 1 | pMx | FEMALE | L | CRC2 | pT |
| CRC2_029_T1 | 3 | 0 | 74 | 3 | 0 | pMx | MALE | L | CRC2 | pT |
| CRC2_030_T1 | 2 | 0 | 52 | 3 | 2 | pMx | MALE | L | CRC2 | pT |
| CRC2_031_T1 | 3 | 0 | 70 | 3 | 1 | pMx | MALE | L | CRC2 | pT |
| CRC2_032_T1 | 1 | 0 | 83 | 3 | 0 | pMx | MALE | L | CRC2 | pT |
| CRC2_033_T1 | 2 | 0 | 84 | 3 | 0 | pMx | MALE | L | CRC2 | pT |
| CRC2_034_T1 | 2 | 50 | 82 | 3 | 0 | pMx | MALE | L | CRC2 | pT |
| CRC2_035_T1 | 1 | 50 | 57 | 4 | 1 | pMx | MALE | L | CRC2 | pT |
| CRC2_036_T1 | 2 | 50 | 86 | 3 | 1 | pMx | MALE | R | CRC2 | pT |
| CRC2_038_T1 | 4 | 0 | 76 | 3 | 1 | pMx | MALE | L | CRC2 | pT |
| CRC2_039_T1 | 1 | 0 | 64 | 3 | 2 | pMx | MALE | L | CRC2 | pT |
| CRC2_039_T2 | 0 | 0 | 64 | 3 | 2 | pMx | MALE | L | CRC2 | pT |
| CRC2_040_T1 | 3 | 0 | 73 | 3 | 0 | pMx | MALE | R | CRC2 | pT |
| CRC2_041_T1 | 2 | 50 | 77 | 3 | 0 | pMx | MALE | L | CRC2 | pT |
| CRC2_043_T1 | 1 | 50 | 69 | 2 | 0 | pMx | MALE | L | CRC2 | pT |
| CRC2_044_T1 | 1 | 0 | 81 | 3 | 0 | pMx | MALE | L | CRC2 | pT |
| CRC2_045_T1 | 0 | 0 | NA | NA | NA | pMx | NA | NA | CRC2 | pT |
| CRC2_046_T1 | 0 | 0 | NA | NA | NA | pMx | NA | NA | CRC2 | pT |
| CRC2_047_T1 | 0 | 0 | NA | NA | NA | pMx | NA | NA | CRC2 | pT |
| CRC2_049_T1 | 0 | 0 | NA | NA | NA | pMx | NA | NA | CRC2 | pT |
| CRC2_053_T1 | 0 | 0 | NA | NA | NA | pMx | NA | NA | CRC2 | pT |
| CRC2_055_T1 | 0 | 0 | NA | NA | NA | pMx | NA | NA | CRC2 | pT |
| CRC1_LT1 | 0 | 0 | 59 | NA | NA | pMx | MALE | NA | CRC1 | M |
| CRC1_LT10 | 1 | 0 | 74 | NA | NA | pMx | NA | NA | CRC1 | M |
| CRC1_LT11 | 1 | 0 | 39 | NA | NA | pMx | NA | NA | CRC1 | M |
| CRC1_LT12 | 2 | 0 | 67 | NA | NA | pMx | NA | NA | CRC1 | M |
| CRC1_LT13 | 0 | 0 | 60 | NA | NA | pMx | NA | NA | CRC1 | M |
| CRC1_LT2 | 0 | 0 | 66 | NA | NA | pMx | NA | NA | CRC1 | M |
| CRC1_LT3 | 2 | NA | 49 | NA | NA | pMx | NA | NA | CRC1 | M |
| CRC1_LT5 | 2 | 0 | 78 | NA | NA | pMx | NA | NA | CRC1 | M |
| CRC1_LT7 | 1 | 0 | 59 | NA | NA | pMx | NA | NA | CRC1 | M |
| CRC1_LT8 | 1 | 0 | 60 | NA | NA | pMx | NA | NA | CRC1 | M |
| CRC1_LT9 | 2 | 0 | 73 | NA | NA | pMx | NA | NA | CRC1 | M |
| CRC1_T1392A | 0 | 0 | NA | NA | NA | pMx | NA | NA | CRC1 | M |
| CRC1_T1843F | 3 | 0 | NA | NA | NA | pMx | NA | NA | CRC1 | M |
| CRC1_T3144B | 3 | 100 | NA | NA | NA | pMx | NA | NA | CRC1 | M |
