# Supplementary material for: Boosting Wnt activity during colorectal cancer progression through selective hypermethylation of Wnt signaling antagonists
Source: BMC Cancer. 2014 Nov 29;14:891. doi: 10.1186/1471-2407-14-891 (PMC4265460; doi:10.1186/1471-2407-14-891)
Supplement: Supplementary file 3 — Additional file 3: Is supplementary information of the methods used in the synthesis of complementary DNA (cDNA) by reverse transcription, quantitative PCR (qPCR) and cell treatment with 5-Aza-2’-Deoxycitidine. (DOCX 109 KB) [file 12885_2014_5079_MOESM3_ESM.docx]

**Additional data file 3 –** Supplementary Material & Methods

**1. Complementary DNA (cDNA) Synthesis by Reverse Transcription**

**a. Materials**

| SuperScript III First-Strand Synthesis System for RT-PCR  Random hexamers (50ng/µl)  dNTP mix (10mM)  10x RT Buffer  25mM MgCl_2_  100mM Dithiothreitol (DTT)  RNase Out  SuperScript™ III Reverse Transcriptase  RNase H | Invitrogen |
| --- | --- |
| Tetrad Peltier Thermal Cycler | MJ Research |

**b. Methods**

The quality of all RNA samples was assessed by agarose gel eletrophoresis in order to visualize the bands corresponding to 18S and 28S ribosomal RNA fractions. The purity of the RNA samples was assed using the ratio of the absorbance at 260 nm and 280 nm on the Nanodrop ND-1000. All RNA samples included in this study had a ratio of 2.0.cDNA was synthesised from 2 µg of DNA-free total RNA. Random hexamers (2 µl), dNTP mix (2 µl) and MilliQ H_2_O were added to the RNA, up to a final volume of 20 µl, before heating the RNA to 65°C for 5 minutes to relax any secondary structure and allow the primers to anneal. The reaction was then placed on ice and 10x RT buffer (4 µl), MgCl_2_ (8 µl), DTT (4 µl), RNase Out (1.5 µl) and SuperScript™ III enzyme (1 µl) were added to a final reaction volume of 40 µl. The reaction was then heated to 25°C for 5 minutes, then to 50°C for 50 minutes followed by 5 minutes at 80°C before addition of 1 µl of RNase H (for removal of remaining complementary RNA) and a final incubation at 37°C for 20 minutes. cDNA products were stored at -20°C.

**2. Real-Time Quantitative PCR (qPCR)**

**a. Material**

| iCycler iQ Multicolor Real-Time PCR | BioRad |
| --- | --- |
| iQ SYBR-Green Supermix | BioRad |
| iCycler 96-well plate | BioRad |
| iCycler iQ Optical Tape | BioRad |
| PCR primers (10µM) | Biomers/Sigma |
| Primer3 (v.0.4.0) | http://frodo.wi.mit.edu/primer3/ |

b. Methods

Quantitative PCR analysis was performed in an iCycler iQ Multicolor Real-Time PCR detection system using the optical system software v.3.1. iQ SYBR-Green Supermix was used for real-time quantification of PCR products.

Each reaction was carried out in duplicate in a 25µl final volume with 12.5µl of SYBR-Green supermix, 0.625µM of each primer and 1µl of cDNA. PCR amplifications were performed in 96-well plates optimized for the iCycler by the manufacturer. Each run included the samples to be tested (in duplicate), a negative control (H_2_O). The initial denaturation for each run was for 6 minutes at 95°C. This was followed by 45 amplification cycles, of 95°C for 15 seconds, and optimized annealing temperature for 1minute followed by melt curve data collection by progressive denaturation from 55°C-95°C with a ramping rate of 0.5°C per second. Primers and annealing temperatures are listed in the table below:

| **Gene** | **Primers** | **Tem (°C)** | **Size** |
| --- | --- | --- | --- |
| ***SFRP1*** | Fwd_5’- CTACTGGCCCGAGATGCTTA -3’  Rev_5’- GCTGGCACAGAGATGTTCAA-3’ | 62°C | 169bp |
| ***SFRP2*** | Fwd_5’-CATGCTTGAGTGCGACCGTTTCC-3’  Rev_5’-AAGCGTTTCCATTATGTCGTTGTC-3’ | 62°C | 154bp |
| ***SFRP5*** | Fwd_5’- CGCCTCCAGTGACCAAGAT-3’  Rev_5’- GATGCGCATTTTGACCACAAAG-3’ | 60°C | 104bp |
| ****DKK2*** | Fwd_5’- AGTACCCGCTGCAATAATGG-3’  Rev_5'-GAAATGACGAGCACAGCAAA-3' | 60°C | 243bp |
| ***WIF1*** | Fwd_5’- TCTGTTCAAAGCCTGTCTGC-3’  Rev_5’- CCTTTTATTGCAGTGTCTTCCA-3’ | 62°C | 110bp |
| ***AXIN2*** | Fwd_5’-CTGGCTCCAGAAGATCACAAAG-3’  Rev_5’-ATCTCCTCAAACACCGCTCCA-3’ | 60°C | 239bp |
| ***c-MYC*** | Fwd_5’-TACCCTCTCAACGACAGCAG-3’  Rev_5’-TCTTGACATTCTCCTCGGTG-3’ | 60°C | 478bp |
| ***B2M*** | Fwd_5’- ACCCCCACTGAAAAAGATGA -3’  Rev_5’- ATCTTCAAACCTCCATGATG -3’ | 60°C | 114bp |
| ***GAPDH*** | Fwd_5’- GCAAATTCCATGGCACCG -3’  Rev_5’- TCGCCCCACTTGATTTTGG -3’ | 60°C | 106bp |

**DKK2* primers: we only got one band of 243bp when we analysed the PCR product by agarose gel electrophoresis.

**c. Reference genes for relative quantification**

The reference genes used were, *GAPDH* and *B2M*.

**d. Primer efficiency and Data analysis**

A standard curve was generated for each target and reference gene studied using serial dilutions of a control template in order to evaluate the amplification efficiency of each primer set. All primers used showed efficiencies between 90 and 110%. Similar efficiencies are especially important for relative expression quantifications.

We used the Pfaffl method to calculate the expression fold change (1).

**3. Cell Culture & 5-Aza-2’-Deoxycitidine Treatment**

HCT116 colorectal cancer cell line was cultured in MyCoy’s 5A modified medium supplemented with 10% foetal bovine serum and 1% penicillin/streptomycin and allowed to grow at 37˚C on a humidified 5% CO_2_ incubator before being lysed at confluence. Cells were treated with 1.5µ M and 3 µM of 5-aza-2’-deoxycitidine (Sigma-Aldrich, St Louis, MO), final concentration. After 24 hours cells were washed with 1x PBS and the medium was replaced. Cells were cultured for another 48 hours. As a control, untreated treated cells were cultured in parallel. No toxicity effect was observed under the higher drug concentration, the cells remain alive and dividing through the 96 hours, however toxicity was not tested directly.

**References**

1. Pfaffl MW (2001) A new mathematical model for relative quantification in real-time RT-PCR. *Nucleic Acids Res* 29:e45.
