# Supplementary material for: Boosting Wnt activity during colorectal cancer progression through selective hypermethylation of Wnt signaling antagonists
Source: BMC Cancer. 2014 Nov 29;14:891. doi: 10.1186/1471-2407-14-891 (PMC4265460; doi:10.1186/1471-2407-14-891)
Supplement: Supplementary file 4 — Additional file 4: Is a table with the sequences of all Pyrosequencing primers and PCR conditions used. (DOCX 25 KB) [file 12885_2014_5079_MOESM4_ESM.docx]

**Additional data file 4**

**Pyrosequencing primers & PCR conditions**

Pyrosequencing assays to assess methylation of the Wnt signalling components: primers, genomic location, PCR conditions & size

| **Gene** | **Primers** | **Genomic Localization**  **(Human Mar. 2006 (NCBI36/hg18) Assembly.)** | **CpG analysed** | **PCR Conditions** | **Size** |
| --- | --- | --- | --- | --- | --- |
| ***SFRP1*** | Fwd_5’-GGTATTGATTTYGGAGGTTGTAGGG-3’  Rev_5’-Bio_ATCCATTCCCCTTCTTTTTCT-3’  Seq_5’-AGGTTGTAGGGTTGG-3’ | chr8:41,286,114-41,286,388 | CpG130, CpG131, CpG132, CpG133, CpG134, CpG135, CpG136, CpG137, | AmpliTaq Gold Hot-Start enzyme; 2mM MgCl_2_; 57ºC annealing temperature | 275bp |
| ***SFRP2*_1** | Fwd_AAGGGAGGAGTTAATGAAGGGTAAT-3’  Rev_5’-Bio_RCAACRACCAAACTTCTCTATTTATTA-3’  Seq_5’-TTTTTGGGTTTGGTTT | chr4:154,929,803-154,929,875 | CpG71, CpG72, CpG73, CpG74, CpG75, CpG76 | AmpliTaq Gold Hot-Start enzyme; 2mM MgCl_2_; 52.2ºC annealing temperature | 187bp |
| ***SFRP2*_2** | Fwd_5’-GATTTAGAGGGGGTAGAGGG-3’  Rev_5’-Bio_CCCCACCCTCCAAATTTAC-3’  Seq_5’-GYGGAGTYGGGGAAGG-3’ | chr4:154,929,488-154,929,587 | CpG43, CpG44, CpG45, CpG46, CpG47, CpG48, CpG49 | AmpliTaq Gold Hot-Start enzyme; 2mM MgCl_2_; 58ºC annealing temperature | 285bp |
| ***SFRP2*_4** | Fwd_5’-TTTGTTGGGTTGTTAGGTATGA-3’  Rev_5’-Bio_AAACACACCRACAAACTACTA-3’  Seq_5’-TGGAGTTYGGGATTTGT-3’ | chr4:154,930,213-154,930,252 | CpG104, CpG105, CpG106, CpG107, CpG108, CpG109, CpG110, CpG111 | Immolase Hot-Start enzyme; 2mM MgCl_2_; 60ºC annealing temperature | 210bp |
| ***SFRP4*** | Fwd_5’-GTTGYGGTTTAGAGGGGGTGA-5’  Rev_5’-Bio_TCCTACCACCCTCATCTTT-3’  Seq_5’-AGGGGGTGATGTTAT-3’ | chr7:37,922,716-37,923,107 | CpG84, CpG85, CpG86, CpG87, CpG88, CpG89, CpG90, CpG91, CpG92 | AmpliTaq Gold Hot-Start enzyme; 2mM MgCl_2_; 63ºC annealing temperature | 392bp |
| ***SFRP5*** | Fwd_5’-GGTGGGAGGYGTTAGGATTAGT-3’  Rev_5’-Bio_CCCCCRACCCTAACTCTACC-3’  Seq_5’-GTAGGTTGTTATTTATTTGG-3’ | chr10:99,521,658-99,521,765 | CpG85, CpG86, CpG87, CpG88, CpG89, CpG90, CpG91, CpG92 | AmpliTaq Gold Hot-Start enzyme; 2mM MgCl_2_; 64ºC annealing temperature | 108bp |
| ***DKK1*** | Fwd_5’-GGTTTTGTTGTTTTTTTTTTAAGG-3’  Rev_5’-Bio_CCCTACRATCCCAAAATCCTAACT-3’  Seq_5’-GTTTTTTTTTTAAGGGGT-3’ | chr10:53,743,969-53,744,091 | CpG1, CpG2, CpG3, CpG4, CpG5, CpG6, CpG7 | AmpliTaq Gold Hot-Start enzyme; 2mM MgCl_2_; 56ºC annealing temperature | 123bp |
| ***DKK2*** | Fwd_5’-AGAGGATTGGGGAGAGAGTA-3’  Rev_5’-Bio_AAACTTTACAAAACACAATACTCCTTTTCA-3’  Seq_5’-GAGAGTAGAGAGAGAGAA-3’ | chr4:108,176,673-108,176,935 | CpG52, CpG53, CpG54, CpG55, CpG56, CpG67, CpG58, CpG59, CpG 60, CpG61, CpG62 | AmpliTaq Gold Hot-Start enzyme; 2mM MgCl_2_; 55ºC annealing temperature | 263bp |
| ***DKK3***  **(assay1)** | Fwd_5’-GATTTTGTTGAGTTTAGTTTTTTTTG-3’  Rev_5’Bio_TCCACCTCAAACCTCTCT-3’  Seq_5’-TTTTGGTGGATGTGGG-3’ | chr11:11,987,134-11,987,263 | CpG85, CpG84, CpG83, CpG82, CpG81 | AmpliTaq Gold Hot-Start enzyme; 2mM MgCl_2_; 60ºC annealing temperature | 130bp |
| ***DKK3* (assay2)** | Fwd_5’-GGGGYGGAGAGGGAGTTT-3’  Rev_5’Bio_TCCACCTCAAACCTCTCT-3’  Seq_5’-GGGAGYGAGTAGATTTAG-3’ | chr11:11,987,134-11,987,495 | CpG84, CpG85, CpG86, CpG87, CpG88, CpG89, CpG90, CpG91, CpG92, CpG93, CpG94 | Immolase Hot-Start enzyme; 3mM MgCl_2_; 65ºC annealing temperature | 362bp |
| ***WIF1*** | Fwd_5’-ATAGTTTTGGTTGAGGGAGTTGTA-3’  Rev_5’-Bio_ACRCTCCTCCRAACCATACTACT-3’  Seq_5’-TTGGTTGAGGGAGTTG-3’ | chr12:63,801,222-63,801,344 | CpG12, CpG13, CpG14, CpG15, CpG16, CpG17, CpG18 | AmpliTaq Gold Hot-Start enzyme; 2mM MgCl_2_; 65ºC annealing temperature | 123bp |
| ***SOX17*** | Fwd_5’-Bio_GGGGATATGAAGGTGAAGGG-3’  Rev_5’-AACTCACCCAACATCTTACT-3’  Seq_5’-TTATACAAATCTAAATTCTACTA-3’ | chr8:55,533,369-55,533,565 | CpG77, CpG78, CpG79, CpG80, CpG81, CpG82, CpG83, CpG84, CpG85, CpG86 | AmpliTaq Gold Hot-Start enzyme; 2mM MgCl_2_; 62.9ºC annealing temperature | 197bp |
| ***WNT3A_*1** | Fwd_5’-TTGGGGTGYGTTAGATGGGTA-3’  Rev_5’-Bio_AACRCAAACAACTCTAATCTCAAC-3’  Seq_5’-TTTTTTATTTATTAATTTTT-3’ | chr1:226,260,795-226,260,845 | CpG9, CpG10, CpG11, CpG12, CpG13, CpG14 | AmpliTaq Gold Hot-Start enzyme; 2mM MgCl_2_; 65ºC annealing temperature | 322bp |
| ***WNT3A*_3** | Fwd_5’-YGYGGGGTTGGAGTAGAAGGG-3’  Rev_5’-Bio_CRCRCCAACTCCCAAAAC-3’  Seq_5’-GTTGTAGAGGAGTAAGAAGT-3’ | chr1:226,261,416-226,261,465 | CpG98, CpG99, CpG100, CpG101, CpG102, CpG103, CpG104 | AmpliTaq Gold Hot-Start enzyme; 2mM MgCl_2_; 65ºC annealing temperature | 273bp |
| ***WNT5A*** | Fwd_5’-AGTTGGGATGYGTTTAGGAATGGA-3’  Rev_5’-Bio_CCCRACCAAACCCTACCCTTACTA-3’  Seq_5’-GTTTAGGAATGGAGGG-3’ | chr3:55,496,254-55,496,326 | CpG77, CpG78, CpG79, CpG80, CpG81, CpG82, CpG83 | AmpliTaq Gold Hot-Start enzyme; 2mM MgCl_2_; 57ºC annealing temperature | 73bp |
| ***APC*** | Fwd_GGTYGGGAAGYGGAGAGAGAAGTAG-3’  Rev_5’-Bio_TAACTCCAACACCTACCCCATTT-3’  Seq_5’-YGGATTAGGGYGTTTTTTAT-3’ | chr5:112,101,334-112,101,537 | CpG1, CpG2, CpG3, CpG4, CpG5, CpG6, CpG7 | AmpliTaq Gold Hot-Start enzyme; 2mM MgCl_2_; 65ºC annealing temperature | 204bp |
| ***GSK3B*** | Fwd_5’-Bio_YGGAAAAAGTYGATTAGTTTGAGAA-3’  Rev_5’-TCCTCAACTCTTCAAACCTCTT-3’  Seq_5’-CTAATTAACCACTATTACCA-3’ | chr3:121,296,213-121,296,414 | CpG91, CpG92, CpG93, CpG94, CpG95, CpG96, CpG97 | AmpliTaq Gold Hot-Start enzyme; 2mM MgCl_2_; 56ºC annealing temperature | 202bp |
| ***AXIN2*** | Fwd_5’-GGGTTGTTATTGAGTTGTTAGGA-3’  Rev_5’-Bio_AAAATCAAAACTCRAACTAAAAATACCCT-3’  Seq_5’-GTTAGGTTYGYGGAGTTAGTGAT-3’ | chr17:60,988,121-60,988,405 | CpG7, CpG8, CpG9, CpG10, CpG11, CpG12, CpG13, CpG14, CpG15, CpG16, CpG17, CpG18 | AmpliTaq Gold Hot-Start enzyme; 2mM MgCl_2_; 59.3ºC annealing temperature | 285bp |
| ***DVL2*** | Fwd_5’-AGAAAATTTTAGTGTGGTTTAAAGTAGA-3’  Rev_5’-Bio_AACTCCCTACCTAATAAAAATCCCTATC-3’  Seq_5’-ATTTTGATTTATAGATTTGAAAAAG-3’ | chr17:7,078,012-7,078,234 | CpG5, CpG6, CpG7, CpG8, CpG9, CpG10, CpG11, CpG12, CpG13, CpG14 | AmpliTaq Gold Hot-Start enzyme; 2mM MgCl_2_; 55ºC annealing temperature | 223bp |
| ***CTNNB1*_1** | Fwd_5’-Bio_TTGGGGGTGTTGTGAGATTG-3’  Rev_5’-ACCCCCYCYACCTAAATTAA-3’  Seq_5’-CCTAATATCCTCCCCTATCC-3’ | chr3:41,215,617-41,215,656 | CpG47, CpG48, CpG49, CpG50, CpG51, CpG52 | Immolase Hot-Start enzyme; 2mM MgCl_2_; 57ºC annealing temperature | 400bp |
| ***CTNNB1*_3** | Fwd_5’-Bio_AYGGAGGAAGGTTTGAGGAGTAG-3’  Rev_5’-AAAAAACRCCCAAACACCTC-3’  Seq_5’-ACCTCAAAAAAACAAACTC-3’ | chr3:41,216,107-41,216,146 | CpG103, CpG104, CpG105, CpG106, CpG107, CpG108, CpG109, CpG110, | AmpliTaq Gold Hot-Start enzyme; 2mM MgCl_2_; 57ºC annealing temperature | 117bp |
| ***CDH1*** | Fwd_5’-GAGGGGTTYGYGTTGTTGATTGGTTG-3’  Rev_5’-Bio_ACTAACTTCCRCAAACTCACAAATACTT-3’  Seq_5’-GTAGGTGAATTTTTAGTTAATTAG-3’ | chr16:67,328,583-67,328,741 | CpG11, CpG12, CpG13, CpG14, CpG15 | AmpliTaq Gold Hot-Start enzyme; 2mM MgCl_2_; 55ºC annealing temperature | 159bp |
