# Supplementary material for: Boosting Wnt activity during colorectal cancer progression through selective hypermethylation of Wnt signaling antagonists
Source: BMC Cancer. 2014 Nov 29;14:891. doi: 10.1186/1471-2407-14-891 (PMC4265460; doi:10.1186/1471-2407-14-891)
Supplement: Supplementary file 5 — Additional file 5: Is the analysis supplementary document. (DOCX 58 KB) [file 12885_2014_5079_MOESM5_ESM.docx]

**A-Luisa Silva et al**

**Analysis supplementary document**

**Boosting Wnt activity during colorectal cancer progression through selective hypermethylation of Wnt signaling antagonists**

**- Mixed model analysis**

Each patient had multiple tissue samples ranging from low risk normal to metastatic. A mixed effect model analysis was used to account for the correlation between samples from the same individual. For binary outcomes (i.e. mutation status), a logistic mixed effect model was used. For continuous outcomes (i.e. methylation), a linear mixed effect model was used. In each case, an intercept only model was compared to a model which contained a fixed effect for tissue type. Tissue type was considered to be nominal, that is categorical without ordering. Models were compared using the change in the log-likelihood. A summary of the results is given in Table 1. More detailed results are given in the R output in the Appendix.

Table 1 Summary of mixed model analyses

| **Mutation** | **Model fitted** | **Tissue type significant?** | **Additional information** |
| --- | --- | --- | --- |
| APC | Logistic mixed effect model | Yes |  |
| BRAF | Logistic mixed effect model | Yes |  |
| KRAS | Logistic mixed effect model | Yes |  |
|  | | | |
| **Methylation** | **Model fitted** | **Tissue type significant?** | **Additional information** |
| SFRP1 | Linear mixed effect model | Yes |  |
| SFRP2 | Linear mixed effect model | Yes |  |
| SFRP4 | Linear mixed effect model | - | Did not meet model assumptions |
| SFRP5 | Linear mixed effect model | Yes |  |
| DKK1 | Linear mixed effect model | - | Did not meet model assumptions |
| DKK2 | Linear mixed effect model | Yes |  |
| DKK3 | Linear mixed effect model | - | Did not meet model assumptions |
| WIF1 | Linear mixed effect model | Yes |  |
| WNT3A | Linear mixed effect model | Yes |  |
| WNT5A | Linear mixed effect model | - | Did not meet model assumptions |
| APC | Linear mixed effect model | - | Did not meet model assumptions |
| AXIN | Linear mixed effect model | No |  |
| GSK3B | Linear mixed effect model | No |  |
| CTNNB1 | Linear mixed effect model | No |  |
| DVL2 | Linear mixed effect model | No (borderline) |  |
| CDH1 | Linear mixed effect model | Yes |  |
| SOX17 | Linear mixed effect model | Yes |  |

**Appendix**

R version 2.14.1 (2011-12-22)

> #####################

> #####Mixed model#####

> ######MUTATION#######

> #####################

>

> #Change variable names to make them more consistent for analysis

>

> dataset.final$APC.mut <- as.factor(dataset.final$APC_Mut_Prsent.Absent)

> dataset.final$BRAF.mut <- as.factor(dataset.final$BrafMutations)

> dataset.final$KRAS.mut <- as.factor(dataset.final$KRAS.mutation)

>

> #APC model

>

> model.mutation.apc.a <- glmer(APC.mut ~ 1 + (1|patientid),

+ data=dataset.final, na.action=na.omit, family=binomial, REML=F)

>

> model.mutation.apc.b <- glmer(APC.mut ~ as.factor(relevel(tis, "LRN")) + (1|patientid),

+ data=dataset.final, na.action=na.omit, family=binomial, REML=F)

>

> anova(model.mutation.apc.a, model.mutation.apc.b)

Data: dataset.final

Models:

model.mutation.apc.a: APC.mut ~ 1 + (1 | patientid)

model.mutation.apc.b: APC.mut ~ as.factor(relevel(tis, "LRN")) + (1 | patientid)

Df AIC BIC logLik Chisq Chi Df Pr(>Chisq)

model.mutation.apc.a 2 321.87 328.98 -158.94

model.mutation.apc.b 7 221.42 246.29 -103.71 110.45 5 < 2.2e-16 ***

---

Signif. codes: 0 ‘***’ 0.001 ‘**’ 0.01 ‘*’ 0.05 ‘.’ 0.1 ‘ ’ 1

> model.mutation.apc.b.reml <- glmer(APC.mut ~ as.factor(relevel(tis, "LRN")) + (1|patientid),

+ data=dataset.final, na.action=na.omit, family=binomial, REML=T)

>

> summary(model.mutation.apc.b.reml)

Generalized linear mixed model fit by the Laplace approximation

Formula: APC.mut ~ as.factor(relevel(tis, "LRN")) + (1 | patientid)

Data: dataset.final

AIC BIC logLik deviance

221.4 246.3 -103.7 207.4

Random effects:

Groups Name Variance Std.Dev.

patientid (Intercept) 0.5625 0.75

Number of obs: 258, groups: patientid, 121

Fixed effects:

Estimate Std. Error z value Pr(>|z|)

(Intercept) -1.960e+01 9.007e+03 -0.002 0.998

as.factor(relevel(tis, "LRN"))Ad 1.971e+01 9.007e+03 0.002 0.998

as.factor(relevel(tis, "LRN"))HP 1.781e+01 9.007e+03 0.002 0.998

as.factor(relevel(tis, "LRN"))HRN -2.864e-06 9.195e+03 0.000 1.000

as.factor(relevel(tis, "LRN"))M 1.807e+01 9.007e+03 0.002 0.998

as.factor(relevel(tis, "LRN"))pT 1.992e+01 9.007e+03 0.002 0.998

Correlation of Fixed Effects:

(Intr) a.((,"LRN"))A a.((,"LRN"))HP a.((,"LRN"))HR a.((,"LRN"))M

a.((,"LRN"))A -1.000

a.((,"LRN"))HP -1.000 1.000

a.((,"LRN"))HR -0.979 0.979 0.979

a.((,"LRN"))M -1.000 1.000 1.000 0.979

a.((,"LRN"))T -1.000 1.000 1.000 0.979 1.000 >

>

> #BRAF model

>

> model.mutation.braf.a <- glmer(BRAF.mut ~ 1 + (1|patientid),

+ data=dataset.final, na.action=na.omit, family=binomial, REML=F)

>

> model.mutation.braf.b <- glmer(BRAF.mut ~ as.factor(relevel(tis, "LRN")) + (1|patientid),

+ data=dataset.final, na.action=na.omit, family=binomial, REML=F)

>

> anova(model.mutation.braf.a, model.mutation.braf.b)

Data: dataset.final

Models:

model.mutation.braf.a: BRAF.mut ~ 1 + (1 | patientid)

model.mutation.braf.b: BRAF.mut ~ as.factor(relevel(tis, "LRN")) + (1 | patientid)

Df AIC BIC logLik Chisq Chi Df Pr(>Chisq)

model.mutation.braf.a 2 111.45 118.56 -53.724

model.mutation.braf.b 7 100.83 125.73 -43.417 20.613 5 0.0009584 ***

---

Signif. codes: 0 ‘***’ 0.001 ‘**’ 0.01 ‘*’ 0.05 ‘.’ 0.1 ‘ ’ 1

>

> model.mutation.braf.b.reml <- glmer(BRAF.mut ~ as.factor(relevel(tis, "LRN")) + (1|patientid),

+ data=dataset.final, na.action=na.omit, family=binomial, REML=T)

>

> summary(model.mutation.braf.b.reml)

Generalized linear mixed model fit by the Laplace approximation

Formula: BRAF.mut ~ as.factor(relevel(tis, "LRN")) + (1 | patientid)

Data: dataset.final

AIC BIC logLik deviance

100.8 125.7 -43.42 86.83

Random effects:

Groups Name Variance Std.Dev.

patientid (Intercept) 116.35 10.787

Number of obs: 259, groups: patientid, 121

Fixed effects:

Estimate Std. Error z value Pr(>|z|)

(Intercept) -2.563e+01 1.498e+05 0 1

as.factor(relevel(tis, "LRN"))Ad 1.475e+01 1.498e+05 0 1

as.factor(relevel(tis, "LRN"))HP 1.927e+01 1.498e+05 0 1

as.factor(relevel(tis, "LRN"))HRN -1.400e-03 1.498e+05 0 1

as.factor(relevel(tis, "LRN"))M 1.709e+01 1.498e+05 0 1

as.factor(relevel(tis, "LRN"))pT 1.707e+01 1.498e+05 0 1

Correlation of Fixed Effects:

(Intr) a.((,"LRN"))A a.((,"LRN"))HP a.((,"LRN"))HR a.((,"LRN"))M

a.((,"LRN"))A -1.000

a.((,"LRN"))HP -1.000 1.000

a.((,"LRN"))HR -1.000 1.000 1.000

a.((,"LRN"))M -1.000 1.000 1.000 1.000

a.((,"LRN"))T -1.000 1.000 1.000 1.000 1.000

>

> #KRAS model

>

> model.mutation.kras.a <- glmer(KRAS.mut ~ 1 + (1|patientid),

+ data=dataset.final, na.action=na.omit, family=binomial, REML=F)

>

> model.mutation.kras.b <- glmer(KRAS.mut ~ as.factor(relevel(tis, "LRN")) + (1|patientid),

+ data=dataset.final, na.action=na.omit, family=binomial, REML=F)

>

> anova(model.mutation.kras.a, model.mutation.kras.b)

Data: dataset.final

Models:

model.mutation.kras.a: KRAS.mut ~ 1 + (1 | patientid)

model.mutation.kras.b: KRAS.mut ~ as.factor(relevel(tis, "LRN")) + (1 | patientid)

Df AIC BIC logLik Chisq Chi Df Pr(>Chisq)

model.mutation.kras.a 2 252.85 259.92 -124.424

model.mutation.kras.b 7 180.04 204.80 -83.019 82.809 5 < 2.2e-16 ***

---

Signif. codes: 0 ‘***’ 0.001 ‘**’ 0.01 ‘*’ 0.05 ‘.’ 0.1 ‘ ’ 1

>

> model.mutation.kras.b.reml <- glmer(KRAS.mut ~ as.factor(relevel(tis, "LRN")) + (1|patientid),

+ data=dataset.final, na.action=na.omit, family=binomial, REML=T)

>

> summary(model.mutation.kras.b.reml)

Generalized linear mixed model fit by the Laplace approximation

Formula: KRAS.mut ~ as.factor(relevel(tis, "LRN")) + (1 | patientid)

Data: dataset.final

AIC BIC logLik deviance

180 204.8 -83.02 166

Random effects:

Groups Name Variance Std.Dev.

patientid (Intercept) 4.5622 2.1359

Number of obs: 254, groups: patientid, 121

Fixed effects:

Estimate Std. Error z value Pr(>|z|)

(Intercept) -2.025e+01 1.019e+04 -0.002 0.998

as.factor(relevel(tis, "LRN"))Ad 1.700e+01 1.019e+04 0.002 0.999

as.factor(relevel(tis, "LRN"))HP -1.108e-05 1.076e+04 0.000 1.000

as.factor(relevel(tis, "LRN"))HRN -2.488e-05 1.030e+04 0.000 1.000

as.factor(relevel(tis, "LRN"))M 1.756e+01 1.019e+04 0.002 0.999

as.factor(relevel(tis, "LRN"))pT 1.973e+01 1.019e+04 0.002 0.998

Correlation of Fixed Effects:

(Intr) a.((,"LRN"))A a.((,"LRN"))HP a.((,"LRN"))HR a.((,"LRN"))M

a.((,"LRN"))A -1.000

a.((,"LRN"))HP -0.948 0.948

a.((,"LRN"))HR -0.990 0.990 0.938

a.((,"LRN"))M -1.000 1.000 0.948 0.990

a.((,"LRN"))T -1.000 1.000 0.948 0.990 1.000

>

> #####################

> #####Mixed model#####

> #####METHYLATION#####

> #####################

>

> #Change variable names to make them more consistent for analysis

>

> dataset.final$sfrp1.meth <- dataset.final$SFRP1_chr8.41_286_114.41_286_388

> dataset.final$sfrp2.meth <- dataset.final$SFRP2_chr4.154_929_488.154_929_587

> dataset.final$sfrp4.meth <- dataset.final$SFRP4_chr7.37_922_716.37_923_107

> dataset.final$sfrp5.meth <- dataset.final$SFRP5_chr10.99_521_658.99_521_765

> dataset.final$dkk1.meth <- dataset.final$DKK1_chr10.53_743_969.53_744_091

> dataset.final$dkk2.meth <- dataset.final$DKK2_chr4.108_176_673.108_176_935

> dataset.final$dkk3.meth <- dataset.final$DKK3_chr11.11_987_134.11_987_263

> dataset.final$wif1.meth <- dataset.final$WIF1_chr12.63_801_222.63_801_344

> dataset.final$wnt3a.meth <- dataset.final$WNT3a_chr1.226_260_795.226_260_845

> dataset.final$wnt5a.meth <- dataset.final$WNT5a_chr3.55_496_254.55_496_326

> dataset.final$apc.meth <- dataset.final$APC_chr5.112_101_334.112_101_537

> dataset.final$axin2.meth <- dataset.final$AXIN2_chr17.60_988_121.60_988_405

> dataset.final$gsk3b.meth <- dataset.final$GSK3b_chr3.121_296_213.121_296_414

> dataset.final$ctnnb1.meth <- dataset.final$CTNNB1_chr3.41_216_107.41_216_146

> dataset.final$dvl2.meth <- dataset.final$DVL2_chr17.7_078_012.7_078_234

> dataset.final$cdh1.meth <- dataset.final$CDH1_chr16.67_328_583.67_328_741

> dataset.final$sox17.meth <- dataset.final$SOX17_chr8.55_533_369.55_533_565

>

> #SFRP1 model

>

> model.methylation.sfrp1.a <- lme(sfrp1.meth ~ 1, random=~1|patientid,

+ data=dataset.final, na.action=na.omit, method="ML")

>

> model.methylation.sfrp1.b <- lme(sfrp1.meth ~ as.factor(relevel(tis, "LRN")), random=~1|patientid,

+ data=dataset.final, na.action=na.omit, method="ML")

>

> anova(model.methylation.sfrp1.a, model.methylation.sfrp1.b)

Model df AIC BIC logLik Test L.Ratio

model.methylation.sfrp1.a 1 3 2293.008 2303.524 -1143.504

model.methylation.sfrp1.b 2 8 2074.631 2102.674 -1029.316 1 vs 2 228.3767

p-value

model.methylation.sfrp1.a

model.methylation.sfrp1.b <.0001

>

> model.methylation.sfrp1.b.reml <- lme(sfrp1.meth ~ as.factor(relevel(tis, "LRN")), random=~1|patientid,

+ data=dataset.final, na.action=na.omit, method="REML")

>

> summary(model.methylation.sfrp1.b.reml)

Linear mixed-effects model fit by REML

Data: dataset.final

AIC BIC logLik

2049.671 2077.516 -1016.835

Random effects:

Formula: ~1 | patientid

(Intercept) Residual

StdDev: 5.965103 15.06874

Fixed effects: sfrp1.meth ~ as.factor(relevel(tis, "LRN"))

Value Std.Error DF t-value p-value

(Intercept) 14.00000 6.616260 126 2.115999 0.0363

as.factor(relevel(tis, "LRN"))Ad 25.72099 7.138739 126 3.603016 0.0005

as.factor(relevel(tis, "LRN"))HP 14.59741 7.984792 126 1.828151 0.0699

as.factor(relevel(tis, "LRN"))HRN -2.71896 6.847076 126 -0.397097 0.6920

as.factor(relevel(tis, "LRN"))M 16.84289 8.090523 126 2.081805 0.0394

as.factor(relevel(tis, "LRN"))pT 42.28093 6.836822 126 6.184296 0.0000

Correlation:

(Intr) a.((,"LRN"))A a.((,"LRN"))HP

as.factor(relevel(tis, "LRN"))Ad -0.927

as.factor(relevel(tis, "LRN"))HP -0.829 0.775

as.factor(relevel(tis, "LRN"))HRN -0.966 0.906 0.811

as.factor(relevel(tis, "LRN"))M -0.818 0.758 0.678

as.factor(relevel(tis, "LRN"))pT -0.968 0.903 0.809

a.((,"LRN"))HR a.((,"LRN"))M

as.factor(relevel(tis, "LRN"))Ad

as.factor(relevel(tis, "LRN"))HP

as.factor(relevel(tis, "LRN"))HRN

as.factor(relevel(tis, "LRN"))M 0.792

as.factor(relevel(tis, "LRN"))pT 0.942 0.794

Standardized Within-Group Residuals:

Min Q1 Med Q3 Max

-2.884721389 -0.445154980 -0.005018251 0.467076799 3.296424158

Number of Observations: 246

Number of Groups: 115

>

> qqnorm(model.methylation.sfrp1.b.reml, abline=c(0,1))

>

> #SFRP2 model

>

> model.methylation.sfrp2.a <- lme(sfrp2.meth ~ 1, random=~1|patientid,

+ data=dataset.final, na.action=na.omit, method="ML")

>

> model.methylation.sfrp2.b <- lme(sfrp2.meth ~ as.factor(relevel(tis, "LRN")), random=~1|patientid,

+ data=dataset.final, na.action=na.omit, method="ML")

>

> anova(model.methylation.sfrp2.a, model.methylation.sfrp2.b)

Model df AIC BIC logLik Test L.Ratio

model.methylation.sfrp2.a 1 3 2407.543 2418.259 -1200.771

model.methylation.sfrp2.b 2 8 2218.435 2247.012 -1101.217 1 vs 2 199.1078

p-value

model.methylation.sfrp2.a

model.methylation.sfrp2.b <.0001

>

> model.methylation.sfrp2.b.reml <- lme(sfrp2.meth ~ as.factor(relevel(tis, "LRN")), random=~1|patientid,

+ data=dataset.final, na.action=na.omit, method="REML")

>

> summary(model.methylation.sfrp2.b.reml)

Linear mixed-effects model fit by REML

Data: dataset.final

AIC BIC logLik

2193.847 2222.24 -1088.924

Random effects:

Formula: ~1 | patientid

(Intercept) Residual

StdDev: 5.620078 15.20879

Fixed effects: sfrp2.meth ~ as.factor(relevel(tis, "LRN"))

Value Std.Error DF t-value p-value

(Intercept) 10.16667 6.619323 133 1.535907 0.1269

as.factor(relevel(tis, "LRN"))Ad 22.91866 7.128370 133 3.215133 0.0016

as.factor(relevel(tis, "LRN"))HP 12.28955 7.994743 133 1.537203 0.1266

as.factor(relevel(tis, "LRN"))HRN 1.65498 6.839521 133 0.241974 0.8092

as.factor(relevel(tis, "LRN"))M 24.77339 7.822828 133 3.166808 0.0019

as.factor(relevel(tis, "LRN"))pT 40.66545 6.819889 133 5.962774 0.0000

Correlation:

(Intr) a.((,"LRN"))A a.((,"LRN"))HP

as.factor(relevel(tis, "LRN"))Ad -0.929

as.factor(relevel(tis, "LRN"))HP -0.828 0.775

as.factor(relevel(tis, "LRN"))HRN -0.968 0.908 0.810

as.factor(relevel(tis, "LRN"))M -0.846 0.786 0.701

as.factor(relevel(tis, "LRN"))pT -0.971 0.907 0.810

a.((,"LRN"))HR a.((,"LRN"))M

as.factor(relevel(tis, "LRN"))Ad

as.factor(relevel(tis, "LRN"))HP

as.factor(relevel(tis, "LRN"))HRN

as.factor(relevel(tis, "LRN"))M 0.821

as.factor(relevel(tis, "LRN"))pT 0.945 0.823

Standardized Within-Group Residuals:

Min Q1 Med Q3 Max

-2.56259333 -0.46440894 -0.02472092 0.51047338 2.94261925

Number of Observations: 263

Number of Groups: 125

>

> qqnorm(model.methylation.sfrp2.b.reml, abline=c(0,1))

>

> #SFRP4 model

>

> model.methylation.sfrp4.a <- lme(sfrp4.meth ~ 1, random=~1|patientid,

+ data=dataset.final, na.action=na.omit, method="ML")

>

> model.methylation.sfrp4.b <- lme(sfrp4.meth ~ as.factor(relevel(tis, "LRN")), random=~1|patientid,

+ data=dataset.final, na.action=na.omit, method="ML")

>

> anova(model.methylation.sfrp4.a, model.methylation.sfrp4.b)

Model df AIC BIC logLik Test L.Ratio

model.methylation.sfrp4.a 1 3 1854.720 1864.887 -924.3599

model.methylation.sfrp4.b 2 8 1832.233 1859.345 -908.1162 1 vs 2 32.48724

p-value

model.methylation.sfrp4.a

model.methylation.sfrp4.b <.0001

>

> model.methylation.sfrp4.b.reml <- lme(sfrp4.meth ~ as.factor(relevel(tis, "LRN")), random=~1|patientid,

+ data=dataset.final, na.action=na.omit, method="REML")

>

> summary(model.methylation.sfrp4.b.reml)

Linear mixed-effects model fit by REML

Data: dataset.final

AIC BIC logLik

1806.889 1833.78 -895.4446

Random effects:

Formula: ~1 | patientid

(Intercept) Residual

StdDev: 5.881311 14.49766

Fixed effects: sfrp4.meth ~ as.factor(relevel(tis, "LRN"))

Value Std.Error DF t-value p-value

(Intercept) 5.000000 6.387120 113 0.7828254 0.4354

as.factor(relevel(tis, "LRN"))Ad 8.735837 6.892200 113 1.2674963 0.2076

as.factor(relevel(tis, "LRN"))HP 16.768230 7.709137 113 2.1751113 0.0317

as.factor(relevel(tis, "LRN"))HRN 0.050366 6.638492 113 0.0075870 0.9940

as.factor(relevel(tis, "LRN"))M 4.142857 8.704178 100 0.4759619 0.6351

as.factor(relevel(tis, "LRN"))pT 12.268626 6.633045 113 1.8496221 0.0670

Correlation:

(Intr) a.((,"LRN"))A a.((,"LRN"))HP

as.factor(relevel(tis, "LRN"))Ad -0.927

as.factor(relevel(tis, "LRN"))HP -0.829 0.775

as.factor(relevel(tis, "LRN"))HRN -0.962 0.904 0.809

as.factor(relevel(tis, "LRN"))M -0.734 0.680 0.608

as.factor(relevel(tis, "LRN"))pT -0.963 0.900 0.807

a.((,"LRN"))HR a.((,"LRN"))M

as.factor(relevel(tis, "LRN"))Ad

as.factor(relevel(tis, "LRN"))HP

as.factor(relevel(tis, "LRN"))HRN

as.factor(relevel(tis, "LRN"))M 0.706

as.factor(relevel(tis, "LRN"))pT 0.935 0.707

Standardized Within-Group Residuals:

Min Q1 Med Q3 Max

-1.7008614 -0.6328071 -0.1607203 0.1593243 4.2678823

Number of Observations: 219

Number of Groups: 102

>

> qqnorm(model.methylation.sfrp4.b.reml, abline=c(0,1))

> hist(residuals(model.methylation.sfrp4.b.reml, type="p"))

>

> #SFRP4 model - log transformed with small offset (offset due to zero values)

>

> model.methylation.sfrp4.ln.a <- lme(log(sfrp4.meth+0.001) ~ 1, random=~1|patientid,

+ data=dataset.final, na.action=na.omit, method="ML")

>

> model.methylation.sfrp4.ln.b <- lme(log(sfrp4.meth+0.001) ~ as.factor(relevel(tis, "LRN")), random=~1|patientid,

+ data=dataset.final, na.action=na.omit, method="ML")

>

> anova(model.methylation.sfrp4.ln.a, model.methylation.sfrp4.ln.b)

Model df AIC BIC logLik Test

model.methylation.sfrp4.ln.a 1 3 945.5053 955.6725 -469.7527

model.methylation.sfrp4.ln.b 2 8 942.7567 969.8693 -463.3784 1 vs 2

L.Ratio p-value

model.methylation.sfrp4.ln.a

model.methylation.sfrp4.ln.b 12.74858 0.0259

>

> model.methylation.sfrp4.ln.b.reml <- lme(log(sfrp4.meth+0.001) ~ as.factor(relevel(tis, "LRN")), random=~1|patientid,

+ data=dataset.final, na.action=na.omit, method="REML")

>

> summary(model.methylation.sfrp4.ln.b.reml)

Linear mixed-effects model fit by REML

Data: dataset.final

AIC BIC logLik

941.4384 968.3287 -462.7192

Random effects:

Formula: ~1 | patientid

(Intercept) Residual

StdDev: 2.216511 1.341217

Fixed effects: log(sfrp4.meth + 0.001) ~ as.factor(relevel(tis, "LRN"))

Value Std.Error DF t-value p-value

(Intercept) 1.5960361 1.057653 113 1.5090351 0.1341

as.factor(relevel(tis, "LRN"))Ad -0.2505377 1.115255 113 -0.2246461 0.8227

as.factor(relevel(tis, "LRN"))HP 0.4933844 1.168165 113 0.4223586 0.6736

as.factor(relevel(tis, "LRN"))HRN -0.7208452 1.098048 113 -0.6564786 0.5129

as.factor(relevel(tis, "LRN"))M -1.1278218 1.441339 100 -0.7824822 0.4358

as.factor(relevel(tis, "LRN"))pT -0.0404545 1.095657 113 -0.0369226 0.9706

Correlation:

(Intr) a.((,"LRN"))A a.((,"LRN"))HP

as.factor(relevel(tis, "LRN"))Ad -0.948

as.factor(relevel(tis, "LRN"))HP -0.905 0.904

as.factor(relevel(tis, "LRN"))HRN -0.963 0.965 0.921

as.factor(relevel(tis, "LRN"))M -0.734 0.696 0.664

as.factor(relevel(tis, "LRN"))pT -0.965 0.960 0.918

a.((,"LRN"))HR a.((,"LRN"))M

as.factor(relevel(tis, "LRN"))Ad

as.factor(relevel(tis, "LRN"))HP

as.factor(relevel(tis, "LRN"))HRN

as.factor(relevel(tis, "LRN"))M 0.707

as.factor(relevel(tis, "LRN"))pT 0.974 0.708

Standardized Within-Group Residuals:

Min Q1 Med Q3 Max

-4.93638840 -0.27387572 0.03914474 0.36508469 1.82221275

Number of Observations: 219

Number of Groups: 102

>

> qqnorm(model.methylation.sfrp4.ln.b.reml, abline=c(0,1))

> hist(residuals(model.methylation.sfrp4.ln.b.reml, type="p"))

>

> #SFRP5 model

>

> model.methylation.sfrp5.a <- lme(sfrp5.meth ~ 1, random=~1|patientid,

+ data=dataset.final, na.action=na.omit, method="ML")

>

> model.methylation.sfrp5.b <- lme(sfrp5.meth ~ as.factor(relevel(tis, "LRN")), random=~1|patientid,

+ data=dataset.final, na.action=na.omit, method="ML")

>

> anova(model.methylation.sfrp5.a, model.methylation.sfrp5.b)

Model df AIC BIC logLik Test L.Ratio

model.methylation.sfrp5.a 1 3 2386.133 2396.838 -1190.067

model.methylation.sfrp5.b 2 8 2256.085 2284.632 -1120.043 1 vs 2 140.0481

p-value

model.methylation.sfrp5.a

model.methylation.sfrp5.b <.0001

>

> model.methylation.sfrp5.b.reml <- lme(sfrp5.meth ~ as.factor(relevel(tis, "LRN")), random=~1|patientid,

+ data=dataset.final, na.action=na.omit, method="REML")

>

> summary(model.methylation.sfrp5.b.reml)

Linear mixed-effects model fit by REML

Data: dataset.final

AIC BIC logLik

2230.347 2258.708 -1107.173

Random effects:

Formula: ~1 | patientid

(Intercept) Residual

StdDev: 8.55649 15.78932

Fixed effects: sfrp5.meth ~ as.factor(relevel(tis, "LRN"))

Value Std.Error DF t-value p-value

(Intercept) 12.666667 7.331621 132 1.727676 0.0864

as.factor(relevel(tis, "LRN"))Ad 23.265370 7.883773 132 2.951045 0.0037

as.factor(relevel(tis, "LRN"))HP 17.801367 8.782066 132 2.027014 0.0447

as.factor(relevel(tis, "LRN"))HRN -1.551779 7.582697 132 -0.204647 0.8382

as.factor(relevel(tis, "LRN"))M 15.427323 8.723495 132 1.768480 0.0793

as.factor(relevel(tis, "LRN"))pT 30.858223 7.551105 132 4.086584 0.0001

Correlation:

(Intr) a.((,"LRN"))A a.((,"LRN"))HP

as.factor(relevel(tis, "LRN"))Ad -0.930

as.factor(relevel(tis, "LRN"))HP -0.835 0.788

as.factor(relevel(tis, "LRN"))HRN -0.967 0.916 0.823

as.factor(relevel(tis, "LRN"))M -0.840 0.783 0.703

as.factor(relevel(tis, "LRN"))pT -0.971 0.913 0.822

a.((,"LRN"))HR a.((,"LRN"))M

as.factor(relevel(tis, "LRN"))Ad

as.factor(relevel(tis, "LRN"))HP

as.factor(relevel(tis, "LRN"))HRN

as.factor(relevel(tis, "LRN"))M 0.817

as.factor(relevel(tis, "LRN"))pT 0.950 0.820

Standardized Within-Group Residuals:

Min Q1 Med Q3 Max

-2.296099e+00 -4.834926e-01 8.340423e-05 5.118382e-01 2.345320e+00

Number of Observations: 262

Number of Groups: 125

>

> qqnorm(model.methylation.sfrp5.b.reml, abline=c(0,1))

>

> #DKK1 model

>

> model.methylation.dkk1.a <- lme(dkk1.meth ~ 1, random=~1|patientid,

+ data=dataset.final, na.action=na.omit, method="ML")

>

> model.methylation.dkk1.b <- lme(dkk1.meth ~ as.factor(relevel(tis, "LRN")), random=~1|patientid,

+ data=dataset.final, na.action=na.omit, method="ML")

>

> anova(model.methylation.dkk1.a, model.methylation.dkk1.b)

Model df AIC BIC logLik Test L.Ratio

model.methylation.dkk1.a 1 3 2076.411 2087.105 -1035.206

model.methylation.dkk1.b 2 8 2067.723 2096.239 -1025.861 1 vs 2 18.68829

p-value

model.methylation.dkk1.a

model.methylation.dkk1.b 0.0022

>

> model.methylation.dkk1.b.reml <- lme(dkk1.meth ~ as.factor(relevel(tis, "LRN")), random=~1|patientid,

+ data=dataset.final, na.action=na.omit, method="REML")

>

> summary(model.methylation.dkk1.b.reml)

Linear mixed-effects model fit by REML

Data: dataset.final

AIC BIC logLik

2046.173 2074.503 -1015.086

Random effects:

Formula: ~1 | patientid

(Intercept) Residual

StdDev: 3.017693 12.11723

Fixed effects: dkk1.meth ~ as.factor(relevel(tis, "LRN"))

Value Std.Error DF t-value p-value

(Intercept) 6.500000 5.097938 132 1.2750254 0.2045

as.factor(relevel(tis, "LRN"))Ad 2.078276 5.490012 132 0.3785558 0.7056

as.factor(relevel(tis, "LRN"))HP 12.580962 6.169325 132 2.0392769 0.0434

as.factor(relevel(tis, "LRN"))HRN -1.036603 5.265707 132 -0.1968592 0.8442

as.factor(relevel(tis, "LRN"))M 1.030676 6.091200 132 0.1692074 0.8659

as.factor(relevel(tis, "LRN"))pT 4.394312 5.252206 132 0.8366603 0.4043

Correlation:

(Intr) a.((,"LRN"))A a.((,"LRN"))HP

as.factor(relevel(tis, "LRN"))Ad -0.929

as.factor(relevel(tis, "LRN"))HP -0.826 0.770

as.factor(relevel(tis, "LRN"))HRN -0.968 0.904 0.805

as.factor(relevel(tis, "LRN"))M -0.837 0.777 0.692

as.factor(relevel(tis, "LRN"))pT -0.971 0.904 0.805

a.((,"LRN"))HR a.((,"LRN"))M

as.factor(relevel(tis, "LRN"))Ad

as.factor(relevel(tis, "LRN"))HP

as.factor(relevel(tis, "LRN"))HRN

as.factor(relevel(tis, "LRN"))M 0.811

as.factor(relevel(tis, "LRN"))pT 0.943 0.813

Standardized Within-Group Residuals:

Min Q1 Med Q3 Max

-1.44467779 -0.47644893 -0.20474796 0.00924862 5.13691069

Number of Observations: 261

Number of Groups: 124

>

> qqnorm(model.methylation.dkk1.b.reml, abline=c(0,1))

> hist(residuals(model.methylation.dkk1.b.reml, type="p"))

>

> #DKK1 model - log transformed with small offset (offset due to zero values)

>

> model.methylation.dkk1.ln.a <- lme(log(dkk1.meth+0.001) ~ 1, random=~1|patientid,

+ data=dataset.final, na.action=na.omit, method="ML")

>

> model.methylation.dkk1.ln.b <- lme(log(dkk1.meth+0.001) ~ as.factor(relevel(tis, "LRN")), random=~1|patientid,

+ data=dataset.final, na.action=na.omit, method="ML")

>

> anova(model.methylation.dkk1.ln.a, model.methylation.dkk1.ln.b)

Model df AIC BIC logLik Test

model.methylation.dkk1.ln.a 1 3 745.0038 755.6974 -369.5019

model.methylation.dkk1.ln.b 2 8 752.4750 780.9912 -368.2375 1 vs 2

L.Ratio p-value

model.methylation.dkk1.ln.a

model.methylation.dkk1.ln.b 2.528785 0.7722

>

> model.methylation.dkk1.ln.a.reml <- lme(log(dkk1.meth+0.001) ~ 1, random=~1|patientid,

+ data=dataset.final, na.action=na.omit, method="REML")

>

> summary(model.methylation.dkk1.ln.a.reml)

Linear mixed-effects model fit by REML

Data: dataset.final

AIC BIC logLik

748.6248 759.3069 -371.3124

Random effects:

Formula: ~1 | patientid

(Intercept) Residual

StdDev: 0.2528244 0.9680387

Fixed effects: log(dkk1.meth + 0.001) ~ 1

Value Std.Error DF t-value p-value

(Intercept) 1.649316 0.06538526 137 25.22458 0

Standardized Within-Group Residuals:

Min Q1 Med Q3 Max

-8.34308837 -0.48878524 -0.07100764 0.16282840 2.60572741

Number of Observations: 261

Number of Groups: 124

>

> qqnorm(model.methylation.dkk1.ln.a.reml, abline=c(0,1))

> hist(residuals(model.methylation.dkk1.ln.a.reml, type="p"))

>

> #DKK2 model

>

> model.methylation.dkk2.a <- lme(dkk2.meth ~ 1, random=~1|patientid,

+ data=dataset.final, na.action=na.omit, method="ML")

>

> model.methylation.dkk2.b <- lme(dkk2.meth ~ as.factor(relevel(tis, "LRN")), random=~1|patientid,

+ data=dataset.final, na.action=na.omit, method="ML")

>

> anova(model.methylation.dkk2.a, model.methylation.dkk2.b)

Model df AIC BIC logLik Test L.Ratio

model.methylation.dkk2.a 1 3 2403.527 2414.186 -1198.764

model.methylation.dkk2.b 2 8 2237.310 2265.734 -1110.655 1 vs 2 176.2166

p-value

model.methylation.dkk2.a

model.methylation.dkk2.b <.0001

>

> model.methylation.dkk2.b.reml <- lme(dkk2.meth ~ as.factor(relevel(tis, "LRN")), random=~1|patientid,

+ data=dataset.final, na.action=na.omit, method="REML")

>

> summary(model.methylation.dkk2.b.reml)

Linear mixed-effects model fit by REML

Data: dataset.final

AIC BIC logLik

2211.203 2239.439 -1097.602

Random effects:

Formula: ~1 | patientid

(Intercept) Residual

StdDev: 5.895438 17.23651

Fixed effects: dkk2.meth ~ as.factor(relevel(tis, "LRN"))

Value Std.Error DF t-value p-value

(Intercept) 12.50000 7.436997 129 1.680786 0.0952

as.factor(relevel(tis, "LRN"))Ad 28.18615 8.009787 129 3.518963 0.0006

as.factor(relevel(tis, "LRN"))HP 15.86281 8.988935 129 1.764704 0.0800

as.factor(relevel(tis, "LRN"))HRN -0.06076 7.688359 129 -0.007903 0.9937

as.factor(relevel(tis, "LRN"))M 33.35576 8.883336 129 3.754869 0.0003

as.factor(relevel(tis, "LRN"))pT 39.75444 7.666992 129 5.185142 0.0000

Correlation:

(Intr) a.((,"LRN"))A a.((,"LRN"))HP

as.factor(relevel(tis, "LRN"))Ad -0.928

as.factor(relevel(tis, "LRN"))HP -0.827 0.773

as.factor(relevel(tis, "LRN"))HRN -0.967 0.906 0.808

as.factor(relevel(tis, "LRN"))M -0.837 0.778 0.693

as.factor(relevel(tis, "LRN"))pT -0.970 0.905 0.808

a.((,"LRN"))HR a.((,"LRN"))M

as.factor(relevel(tis, "LRN"))Ad

as.factor(relevel(tis, "LRN"))HP

as.factor(relevel(tis, "LRN"))HRN

as.factor(relevel(tis, "LRN"))M 0.811

as.factor(relevel(tis, "LRN"))pT 0.944 0.813

Standardized Within-Group Residuals:

Min Q1 Med Q3 Max

-2.61022162 -0.48569799 -0.04352912 0.44112383 2.69034103

Number of Observations: 258

Number of Groups: 124

>

> qqnorm(model.methylation.dkk2.b.reml, abline=c(0,1))

>

> #DKK3 model

>

> model.methylation.dkk3.a <- lme(dkk3.meth ~ 1, random=~1|patientid,

+ data=dataset.final, na.action=na.omit, method="ML")

>

> model.methylation.dkk3.b <- lme(dkk3.meth ~ as.factor(relevel(tis, "LRN")), random=~1|patientid,

+ data=dataset.final, na.action=na.omit, method="ML")

>

> anova(model.methylation.dkk3.a, model.methylation.dkk3.b)

Model df AIC BIC logLik Test L.Ratio

model.methylation.dkk3.a 1 3 1982.233 1992.868 -988.1163

model.methylation.dkk3.b 2 8 1946.950 1975.311 -965.4750 1 vs 2 45.28258

p-value

model.methylation.dkk3.a

model.methylation.dkk3.b <.0001

>

> model.methylation.dkk3.b.reml <- lme(dkk3.meth ~ as.factor(relevel(tis, "LRN")), random=~1|patientid,

+ data=dataset.final, na.action=na.omit, method="REML")

>

> summary(model.methylation.dkk3.b.reml)

Linear mixed-effects model fit by REML

Data: dataset.final

AIC BIC logLik

1927.116 1955.288 -955.558

Random effects:

Formula: ~1 | patientid

(Intercept) Residual

StdDev: 5.010465 9.606351

Fixed effects: dkk3.meth ~ as.factor(relevel(tis, "LRN"))

Value Std.Error DF t-value p-value

(Intercept) 8.666667 4.423173 130 1.9593779 0.0522

as.factor(relevel(tis, "LRN"))Ad 7.184516 4.757433 130 1.5101665 0.1334

as.factor(relevel(tis, "LRN"))HP 2.956219 5.305265 130 0.5572236 0.5783

as.factor(relevel(tis, "LRN"))HRN -0.423747 4.573481 130 -0.0926531 0.9263

as.factor(relevel(tis, "LRN"))M 8.554455 5.331515 130 1.6045072 0.1110

as.factor(relevel(tis, "LRN"))pT 9.270451 4.564097 130 2.0311688 0.0443

Correlation:

(Intr) a.((,"LRN"))A a.((,"LRN"))HP

as.factor(relevel(tis, "LRN"))Ad -0.930

as.factor(relevel(tis, "LRN"))HP -0.834 0.786

as.factor(relevel(tis, "LRN"))HRN -0.967 0.916 0.822

as.factor(relevel(tis, "LRN"))M -0.830 0.772 0.693

as.factor(relevel(tis, "LRN"))pT -0.969 0.911 0.819

a.((,"LRN"))HR a.((,"LRN"))M

as.factor(relevel(tis, "LRN"))Ad

as.factor(relevel(tis, "LRN"))HP

as.factor(relevel(tis, "LRN"))HRN

as.factor(relevel(tis, "LRN"))M 0.806

as.factor(relevel(tis, "LRN"))pT 0.948 0.806

Standardized Within-Group Residuals:

Min Q1 Med Q3 Max

-1.5160958 -0.5433556 -0.1022403 0.2166539 4.4009980

Number of Observations: 256

Number of Groups: 121

>

> qqnorm(model.methylation.dkk3.b.reml, abline=c(0,1))

> hist(residuals(model.methylation.dkk3.b.reml, type="p"))

>

> #DKK3 model - log transformed with small offset (offset due to zero values)

>

> model.methylation.dkk3.ln.a <- lme(log(dkk3.meth+0.001) ~ 1, random=~1|patientid,

+ data=dataset.final, na.action=na.omit, method="ML")

>

> model.methylation.dkk3.ln.b <- lme(log(dkk3.meth+0.001) ~ as.factor(relevel(tis, "LRN")), random=~1|patientid,

+ data=dataset.final, na.action=na.omit, method="ML")

>

> anova(model.methylation.dkk3.ln.a, model.methylation.dkk3.ln.b)

Model df AIC BIC logLik Test

model.methylation.dkk3.ln.a 1 3 478.7966 489.4321 -236.3983

model.methylation.dkk3.ln.b 2 8 431.0200 459.3814 -207.5100 1 vs 2

L.Ratio p-value

model.methylation.dkk3.ln.a

model.methylation.dkk3.ln.b 57.77658 <.0001

>

> model.methylation.dkk3.ln.b.reml <- lme(log(dkk3.meth+0.001) ~ as.factor(relevel(tis, "LRN")), random=~1|patientid,

+ data=dataset.final, na.action=na.omit, method="REML")

>

> summary(model.methylation.dkk3.ln.b.reml)

Linear mixed-effects model fit by REML

Data: dataset.final

AIC BIC logLik

446.7222 474.8939 -215.3611

Random effects:

Formula: ~1 | patientid

(Intercept) Residual

StdDev: 0.2437954 0.5029538

Fixed effects: log(dkk3.meth + 0.001) ~ as.factor(relevel(tis, "LRN"))

Value Std.Error DF t-value p-value

(Intercept) 2.1496406 0.2281807 130 9.420780 0.0000

as.factor(relevel(tis, "LRN"))Ad 0.3809513 0.2455323 130 1.551532 0.1232

as.factor(relevel(tis, "LRN"))HP 0.2146508 0.2742682 130 0.782631 0.4353

as.factor(relevel(tis, "LRN"))HRN -0.1013350 0.2359025 130 -0.429563 0.6682

as.factor(relevel(tis, "LRN"))M 0.2630832 0.2752083 130 0.955942 0.3409

as.factor(relevel(tis, "LRN"))pT 0.4938043 0.2354565 130 2.097221 0.0379

Correlation:

(Intr) a.((,"LRN"))A a.((,"LRN"))HP

as.factor(relevel(tis, "LRN"))Ad -0.929

as.factor(relevel(tis, "LRN"))HP -0.832 0.783

as.factor(relevel(tis, "LRN"))HRN -0.967 0.914 0.819

as.factor(relevel(tis, "LRN"))M -0.829 0.771 0.691

as.factor(relevel(tis, "LRN"))pT -0.969 0.910 0.816

a.((,"LRN"))HR a.((,"LRN"))M

as.factor(relevel(tis, "LRN"))Ad

as.factor(relevel(tis, "LRN"))HP

as.factor(relevel(tis, "LRN"))HRN

as.factor(relevel(tis, "LRN"))M 0.805

as.factor(relevel(tis, "LRN"))pT 0.947 0.805

Standardized Within-Group Residuals:

Min Q1 Med Q3 Max

-3.29610954 -0.61036288 -0.04894668 0.47261041 2.97840389

Number of Observations: 256

Number of Groups: 121

>

> qqnorm(model.methylation.dkk3.ln.b.reml, abline=c(0,1))

> hist(residuals(model.methylation.dkk3.ln.b.reml, type="p"))

>

> #WIF1 model

>

> model.methylation.wif1.a <- lme(wif1.meth ~ 1, random=~1|patientid,

+ data=dataset.final, na.action=na.omit, method="ML")

>

> model.methylation.wif1.b <- lme(wif1.meth ~ as.factor(relevel(tis, "LRN")), random=~1|patientid,

+ data=dataset.final, na.action=na.omit, method="ML")

>

> anova(model.methylation.wif1.a, model.methylation.wif1.b)

Model df AIC BIC logLik Test L.Ratio

model.methylation.wif1.a 1 3 2522.265 2532.959 -1258.133

model.methylation.wif1.b 2 8 2309.946 2338.463 -1146.973 1 vs 2 222.3188

p-value

model.methylation.wif1.a

model.methylation.wif1.b <.0001

>

> model.methylation.wif1.b.reml <- lme(wif1.meth ~ as.factor(relevel(tis, "LRN")), random=~1|patientid,

+ data=dataset.final, na.action=na.omit, method="REML")

>

> summary(model.methylation.wif1.b.reml)

Linear mixed-effects model fit by REML

Data: dataset.final

AIC BIC logLik

2282.796 2311.127 -1133.398

Random effects:

Formula: ~1 | patientid

(Intercept) Residual

StdDev: 6.441694 18.85381

Fixed effects: wif1.meth ~ as.factor(relevel(tis, "LRN"))

Value Std.Error DF t-value p-value

(Intercept) 19.50000 8.133894 132 2.397376 0.0179

as.factor(relevel(tis, "LRN"))Ad 28.58656 8.760093 132 3.263272 0.0014

as.factor(relevel(tis, "LRN"))HP 30.65998 9.831254 132 3.118623 0.0022

as.factor(relevel(tis, "LRN"))HRN -3.81292 8.406226 132 -0.453583 0.6509

as.factor(relevel(tis, "LRN"))M 17.30933 9.712836 132 1.782109 0.0770

as.factor(relevel(tis, "LRN"))pT 48.31066 8.380334 132 5.764765 0.0000

Correlation:

(Intr) a.((,"LRN"))A a.((,"LRN"))HP

as.factor(relevel(tis, "LRN"))Ad -0.929

as.factor(relevel(tis, "LRN"))HP -0.827 0.773

as.factor(relevel(tis, "LRN"))HRN -0.968 0.907 0.809

as.factor(relevel(tis, "LRN"))M -0.837 0.778 0.693

as.factor(relevel(tis, "LRN"))pT -0.971 0.906 0.808

a.((,"LRN"))HR a.((,"LRN"))M

as.factor(relevel(tis, "LRN"))Ad

as.factor(relevel(tis, "LRN"))HP

as.factor(relevel(tis, "LRN"))HRN

as.factor(relevel(tis, "LRN"))M 0.812

as.factor(relevel(tis, "LRN"))pT 0.944 0.814

Standardized Within-Group Residuals:

Min Q1 Med Q3 Max

-3.47692918 -0.41222370 0.03984099 0.60418709 2.33632597

Number of Observations: 261

Number of Groups: 124

>

> qqnorm(model.methylation.wif1.b.reml, abline=c(0,1))

>

> #WNT3a model

>

> model.methylation.wnt3a.a <- lme(wnt3a.meth ~ 1, random=~1|patientid,

+ data=dataset.final, na.action=na.omit, method="ML")

>

> model.methylation.wnt3a.b <- lme(wnt3a.meth ~ as.factor(relevel(tis, "LRN")), random=~1|patientid,

+ data=dataset.final, na.action=na.omit, method="ML")

>

> anova(model.methylation.wnt3a.a, model.methylation.wnt3a.b)

Model df AIC BIC logLik Test L.Ratio

model.methylation.wnt3a.a 1 3 2268.948 2279.583 -1131.474

model.methylation.wnt3a.b 2 8 2124.919 2153.280 -1054.460 1 vs 2 154.0287

p-value

model.methylation.wnt3a.a

model.methylation.wnt3a.b <.0001

>

> model.methylation.wnt3a.b.reml <- lme(wnt3a.meth ~ as.factor(relevel(tis, "LRN")), random=~1|patientid,

+ data=dataset.final, na.action=na.omit, method="REML")

>

> summary(model.methylation.wnt3a.b.reml)

Linear mixed-effects model fit by REML

Data: dataset.final

AIC BIC logLik

2100.945 2129.116 -1042.472

Random effects:

Formula: ~1 | patientid

(Intercept) Residual

StdDev: 5.466506 14.14715

Fixed effects: wnt3a.meth ~ as.factor(relevel(tis, "LRN"))

Value Std.Error DF t-value p-value

(Intercept) 7.500000 6.191721 130 1.211295 0.2280

as.factor(relevel(tis, "LRN"))Ad 17.660430 6.667568 130 2.648706 0.0091

as.factor(relevel(tis, "LRN"))HP 16.029814 7.474504 130 2.144599 0.0338

as.factor(relevel(tis, "LRN"))HRN -0.616202 6.400643 130 -0.096272 0.9235

as.factor(relevel(tis, "LRN"))M 7.145133 7.474815 130 0.955894 0.3409

as.factor(relevel(tis, "LRN"))pT 29.937988 6.387230 130 4.687163 0.0000

Correlation:

(Intr) a.((,"LRN"))A a.((,"LRN"))HP

as.factor(relevel(tis, "LRN"))Ad -0.929

as.factor(relevel(tis, "LRN"))HP -0.828 0.776

as.factor(relevel(tis, "LRN"))HRN -0.967 0.909 0.811

as.factor(relevel(tis, "LRN"))M -0.828 0.770 0.687

as.factor(relevel(tis, "LRN"))pT -0.969 0.906 0.810

a.((,"LRN"))HR a.((,"LRN"))M

as.factor(relevel(tis, "LRN"))Ad

as.factor(relevel(tis, "LRN"))HP

as.factor(relevel(tis, "LRN"))HRN

as.factor(relevel(tis, "LRN"))M 0.803

as.factor(relevel(tis, "LRN"))pT 0.944 0.805

Standardized Within-Group Residuals:

Min Q1 Med Q3 Max

-2.13566333 -0.52513345 -0.02693748 0.29104603 3.65049039

Number of Observations: 256

Number of Groups: 121

>

> qqnorm(model.methylation.wnt3a.b.reml, abline=c(0,1))

>

> #WNT5a model

>

> model.methylation.wnt5a.a <- lme(wnt5a.meth ~ 1, random=~1|patientid,

+ data=dataset.final, na.action=na.omit, method="ML")

>

> model.methylation.wnt5a.b <- lme(wnt5a.meth ~ as.factor(relevel(tis, "LRN")), random=~1|patientid,

+ data=dataset.final, na.action=na.omit, method="ML")

>

> anova(model.methylation.wnt5a.a, model.methylation.wnt5a.b)

Model df AIC BIC logLik Test L.Ratio

model.methylation.wnt5a.a 1 3 2057.250 2067.932 -1025.625

model.methylation.wnt5a.b 2 8 2037.101 2065.586 -1010.551 1 vs 2 30.14913

p-value

model.methylation.wnt5a.a

model.methylation.wnt5a.b <.0001

>

> model.methylation.wnt5a.b.reml <- lme(wnt5a.meth ~ as.factor(relevel(tis, "LRN")), random=~1|patientid,

+ data=dataset.final, na.action=na.omit, method="REML")

>

> summary(model.methylation.wnt5a.b.reml)

Linear mixed-effects model fit by REML

Data: dataset.final

AIC BIC logLik

2015.971 2044.269 -999.9853

Random effects:

Formula: ~1 | patientid

(Intercept) Residual

StdDev: 3.876617 11.34737

Fixed effects: wnt5a.meth ~ as.factor(relevel(tis, "LRN"))

Value Std.Error DF t-value p-value

(Intercept) 3.666667 4.895424 132 0.7489989 0.4552

as.factor(relevel(tis, "LRN"))Ad 5.197823 5.272284 132 0.9858768 0.3260

as.factor(relevel(tis, "LRN"))HP 10.165649 5.916977 132 1.7180478 0.0881

as.factor(relevel(tis, "LRN"))HRN -0.480297 5.057549 132 -0.0949664 0.9245

as.factor(relevel(tis, "LRN"))M 3.962420 5.912758 132 0.6701475 0.5039

as.factor(relevel(tis, "LRN"))pT 8.259070 5.045258 132 1.6369964 0.1040

Correlation:

(Intr) a.((,"LRN"))A a.((,"LRN"))HP

as.factor(relevel(tis, "LRN"))Ad -0.929

as.factor(relevel(tis, "LRN"))HP -0.827 0.773

as.factor(relevel(tis, "LRN"))HRN -0.968 0.907 0.809

as.factor(relevel(tis, "LRN"))M -0.828 0.769 0.685

as.factor(relevel(tis, "LRN"))pT -0.970 0.906 0.808

a.((,"LRN"))HR a.((,"LRN"))M

as.factor(relevel(tis, "LRN"))Ad

as.factor(relevel(tis, "LRN"))HP

as.factor(relevel(tis, "LRN"))HRN

as.factor(relevel(tis, "LRN"))M 0.803

as.factor(relevel(tis, "LRN"))pT 0.944 0.805

Standardized Within-Group Residuals:

Min Q1 Med Q3 Max

-1.36359087 -0.54654948 -0.19360684 0.08989697 4.98688759

Number of Observations: 260

Number of Groups: 123

>

> qqnorm(model.methylation.wnt5a.b.reml, abline=c(0,1))

> hist(residuals(model.methylation.wnt5a.b.reml, type="p"))

>

> #WNT5a model - log transformed with small offset (offset due to zero values)

>

> model.methylation.wnt5a.ln.a <- lme(log(wnt5a.meth+0.001) ~ 1, random=~1|patientid,

+ data=dataset.final, na.action=na.omit, method="ML")

>

> model.methylation.wnt5a.ln.b <- lme(log(wnt5a.meth+0.001) ~ as.factor(relevel(tis, "LRN")), random=~1|patientid,

+ data=dataset.final, na.action=na.omit, method="ML")

>

> anova(model.methylation.wnt5a.ln.a, model.methylation.wnt5a.ln.b)

Model df AIC BIC logLik Test

model.methylation.wnt5a.ln.a 1 3 745.4531 756.1351 -369.7265

model.methylation.wnt5a.ln.b 2 8 706.6435 735.1289 -345.3217 1 vs 2

L.Ratio p-value

model.methylation.wnt5a.ln.a

model.methylation.wnt5a.ln.b 48.80964 <.0001

>

> model.methylation.wnt5a.ln.b.reml <- lme(log(wnt5a.meth+0.001) ~ as.factor(relevel(tis, "LRN")), random=~1|patientid,

+ data=dataset.final, na.action=na.omit, method="REML")

>

> summary(model.methylation.wnt5a.ln.b.reml)

Linear mixed-effects model fit by REML

Data: dataset.final

AIC BIC logLik

716.1772 744.4758 -350.0886

Random effects:

Formula: ~1 | patientid

(Intercept) Residual

StdDev: 0.4346119 0.8346874

Fixed effects: log(wnt5a.meth + 0.001) ~ as.factor(relevel(tis, "LRN"))

Value Std.Error DF t-value p-value

(Intercept) 1.2906781 0.3841854 132 3.359519 0.0010

as.factor(relevel(tis, "LRN"))Ad 0.2485397 0.4132122 132 0.601482 0.5486

as.factor(relevel(tis, "LRN"))HP 0.8724974 0.4608119 132 1.893392 0.0605

as.factor(relevel(tis, "LRN"))HRN -0.2641059 0.3972314 132 -0.664867 0.5073

as.factor(relevel(tis, "LRN"))M 0.3424898 0.4627490 132 0.740120 0.4605

as.factor(relevel(tis, "LRN"))pT 0.5848861 0.3959284 132 1.477252 0.1420

Correlation:

(Intr) a.((,"LRN"))A a.((,"LRN"))HP

as.factor(relevel(tis, "LRN"))Ad -0.930

as.factor(relevel(tis, "LRN"))HP -0.834 0.786

as.factor(relevel(tis, "LRN"))HRN -0.967 0.916 0.822

as.factor(relevel(tis, "LRN"))M -0.830 0.773 0.693

as.factor(relevel(tis, "LRN"))pT -0.970 0.912 0.820

a.((,"LRN"))HR a.((,"LRN"))M

as.factor(relevel(tis, "LRN"))Ad

as.factor(relevel(tis, "LRN"))HP

as.factor(relevel(tis, "LRN"))HRN

as.factor(relevel(tis, "LRN"))M 0.807

as.factor(relevel(tis, "LRN"))pT 0.949 0.809

Standardized Within-Group Residuals:

Min Q1 Med Q3 Max

-7.58576250 -0.50351559 -0.08255095 0.35214514 2.50523225

Number of Observations: 260

Number of Groups: 123

>

> qqnorm(model.methylation.wnt5a.ln.b.reml, abline=c(0,1))

> hist(residuals(model.methylation.wnt5a.ln.b.reml, type="p"))

>

> #APC model

>

> model.methylation.apc.a <- lme(apc.meth ~ 1, random=~1|patientid,

+ data=dataset.final, na.action=na.omit, method="ML")

>

> model.methylation.apc.b <- lme(apc.meth ~ as.factor(relevel(tis, "LRN")), random=~1|patientid,

+ data=dataset.final, na.action=na.omit, method="ML")

>

> anova(model.methylation.apc.a, model.methylation.apc.b)

Model df AIC BIC logLik Test L.Ratio

model.methylation.apc.a 1 3 2202.006 2212.722 -1098.003

model.methylation.apc.b 2 8 2172.888 2201.465 -1078.444 1 vs 2 39.11777

p-value

model.methylation.apc.a

model.methylation.apc.b <.0001

>

> model.methylation.apc.b.reml <- lme(apc.meth ~ as.factor(relevel(tis, "LRN")), random=~1|patientid,

+ data=dataset.final, na.action=na.omit, method="REML")

>

> summary(model.methylation.apc.b.reml)

Linear mixed-effects model fit by REML

Data: dataset.final

AIC BIC logLik

2149.317 2177.71 -1066.659

Random effects:

Formula: ~1 | patientid

(Intercept) Residual

StdDev: 6.613495 13.46763

Fixed effects: apc.meth ~ as.factor(relevel(tis, "LRN"))

Value Std.Error DF t-value p-value

(Intercept) 5.333333 6.125295 132 0.8707064 0.3855

as.factor(relevel(tis, "LRN"))Ad 1.508566 6.590424 132 0.2289028 0.8193

as.factor(relevel(tis, "LRN"))HP -3.653715 7.359648 132 -0.4964524 0.6204

as.factor(relevel(tis, "LRN"))HRN -2.485154 6.334146 132 -0.3923424 0.6954

as.factor(relevel(tis, "LRN"))M 9.262622 7.225984 132 1.2818493 0.2021

as.factor(relevel(tis, "LRN"))pT 10.014271 6.308854 132 1.5873359 0.1148

Correlation:

(Intr) a.((,"LRN"))A a.((,"LRN"))HP

as.factor(relevel(tis, "LRN"))Ad -0.929

as.factor(relevel(tis, "LRN"))HP -0.832 0.783

as.factor(relevel(tis, "LRN"))HRN -0.967 0.914 0.819

as.factor(relevel(tis, "LRN"))M -0.848 0.789 0.707

as.factor(relevel(tis, "LRN"))pT -0.971 0.911 0.818

a.((,"LRN"))HR a.((,"LRN"))M

as.factor(relevel(tis, "LRN"))Ad

as.factor(relevel(tis, "LRN"))HP

as.factor(relevel(tis, "LRN"))HRN

as.factor(relevel(tis, "LRN"))M 0.823

as.factor(relevel(tis, "LRN"))pT 0.948 0.826

Standardized Within-Group Residuals:

Min Q1 Med Q3 Max

-1.18809586 -0.62647894 -0.07976727 0.15605972 4.37578858

Number of Observations: 263

Number of Groups: 126

>

> qqnorm(model.methylation.apc.b.reml, abline=c(0,1))

> hist(residuals(model.methylation.apc.b.reml, type="p"))

>

> #APC model - log transformed with small offset (offset due to zero values)

>

> model.methylation.apc.ln.a <- lme(log(apc.meth+0.001) ~ 1, random=~1|patientid,

+ data=dataset.final, na.action=na.omit, method="ML")

>

> model.methylation.apc.ln.b <- lme(log(apc.meth+0.001) ~ as.factor(relevel(tis, "LRN")), random=~1|patientid,

+ data=dataset.final, na.action=na.omit, method="ML")

>

> anova(model.methylation.apc.ln.a, model.methylation.apc.ln.b)

Model df AIC BIC logLik Test L.Ratio

model.methylation.apc.ln.a 1 3 823.8823 834.5988 -408.9412

model.methylation.apc.ln.b 2 8 812.1781 840.7554 -398.0891 1 vs 2 21.70419

p-value

model.methylation.apc.ln.a

model.methylation.apc.ln.b 6e-04

>

> model.methylation.apc.ln.b.reml <- lme(log(apc.meth+0.001) ~ as.factor(relevel(tis, "LRN")), random=~1|patientid,

+ data=dataset.final, na.action=na.omit, method="REML")

>

> summary(model.methylation.apc.ln.b.reml)

Linear mixed-effects model fit by REML

Data: dataset.final

AIC BIC logLik

819.7199 848.1125 -401.8599

Random effects:

Formula: ~1 | patientid

(Intercept) Residual

StdDev: 0.2379191 1.087415

Fixed effects: log(apc.meth + 0.001) ~ as.factor(relevel(tis, "LRN"))

Value Std.Error DF t-value p-value

(Intercept) 1.5525032 0.4544370 132 3.416322 0.0008

as.factor(relevel(tis, "LRN"))Ad -0.1242124 0.4893382 132 -0.253837 0.8000

as.factor(relevel(tis, "LRN"))HP -0.4111903 0.5499730 132 -0.747655 0.4560

as.factor(relevel(tis, "LRN"))HRN -0.4879339 0.4693191 132 -1.039663 0.3004

as.factor(relevel(tis, "LRN"))M 0.5036188 0.5376000 132 0.936791 0.3506

as.factor(relevel(tis, "LRN"))pT 0.1678019 0.4680439 132 0.358518 0.7205

Correlation:

(Intr) a.((,"LRN"))A a.((,"LRN"))HP

as.factor(relevel(tis, "LRN"))Ad -0.929

as.factor(relevel(tis, "LRN"))HP -0.826 0.770

as.factor(relevel(tis, "LRN"))HRN -0.968 0.903 0.804

as.factor(relevel(tis, "LRN"))M -0.845 0.785 0.699

as.factor(relevel(tis, "LRN"))pT -0.971 0.904 0.805

a.((,"LRN"))HR a.((,"LRN"))M

as.factor(relevel(tis, "LRN"))Ad

as.factor(relevel(tis, "LRN"))HP

as.factor(relevel(tis, "LRN"))HRN

as.factor(relevel(tis, "LRN"))M 0.819

as.factor(relevel(tis, "LRN"))pT 0.942 0.821

Standardized Within-Group Residuals:

Min Q1 Med Q3 Max

-7.65078411 -0.54530553 -0.04576096 0.31529141 2.38445377

Number of Observations: 263

Number of Groups: 126

>

> qqnorm(model.methylation.apc.ln.b.reml, abline=c(0,1))

> hist(residuals(model.methylation.apc.ln.b.reml, type="p"))

>

> resid <- residuals(model.methylation.apc.ln.b.reml, type="p")

> plot(resid)

>

> a <- dataset.final$apc.meth[which(complete.cases(dataset.final$apc.meth))]

> plot(a, resid)

>

> #AXIN2 model

>

> model.methylation.axin2.a <- lme(axin2.meth ~ 1, random=~1|patientid,

+ data=dataset.final, na.action=na.omit, method="ML")

>

> model.methylation.axin2.b <- lme(axin2.meth ~ as.factor(relevel(tis, "LRN")), random=~1|patientid,

+ data=dataset.final, na.action=na.omit, method="ML")

>

> anova(model.methylation.axin2.a, model.methylation.axin2.b)

Model df AIC BIC logLik Test L.Ratio

model.methylation.axin2.a 1 3 907.3389 917.9627 -450.6695

model.methylation.axin2.b 2 8 911.8440 940.1741 -447.9220 1 vs 2 5.494936

p-value

model.methylation.axin2.a

model.methylation.axin2.b 0.3585

>

> model.methylation.axin2.a.reml <- lme(axin2.meth ~ 1, random=~1|patientid,

+ data=dataset.final, na.action=na.omit, method="REML")

>

> summary(model.methylation.axin2.a.reml)

Linear mixed-effects model fit by REML

Data: dataset.final

AIC BIC logLik

910.3435 920.9555 -452.1718

Random effects:

Formula: ~1 | patientid

(Intercept) Residual

StdDev: 0.0001418105 1.419575

Fixed effects: axin2.meth ~ 1

Value Std.Error DF t-value p-value

(Intercept) 3.376471 0.08889721 133 37.98174 0

Standardized Within-Group Residuals:

Min Q1 Med Q3 Max

-2.3785086 -0.9696359 -0.2651996 0.4392368 4.6658549

Number of Observations: 255

Number of Groups: 122

>

> qqnorm(model.methylation.axin2.a.reml, abline=c(0,1))

>

> #GSK3b model

>

> model.methylation.gsk3b.a <- lme(gsk3b.meth ~ 1, random=~1|patientid,

+ data=dataset.final, na.action=na.omit, method="ML")

>

> model.methylation.gsk3b.b <- lme(gsk3b.meth ~ as.factor(relevel(tis, "LRN")), random=~1|patientid,

+ data=dataset.final, na.action=na.omit, method="ML")

>

> anova(model.methylation.gsk3b.a, model.methylation.gsk3b.b)

Model df AIC BIC logLik Test L.Ratio

model.methylation.gsk3b.a 1 3 794.1182 804.7302 -394.0591

model.methylation.gsk3b.b 2 8 799.1448 827.4435 -391.5724 1 vs 2 4.973387

p-value

model.methylation.gsk3b.a

model.methylation.gsk3b.b 0.4191

>

> model.methylation.gsk3b.a.reml <- lme(gsk3b.meth ~ 1, random=~1|patientid,

+ data=dataset.final, na.action=na.omit, method="REML")

>

> summary(model.methylation.gsk3b.a.reml)

Linear mixed-effects model fit by REML

Data: dataset.final

AIC BIC logLik

797.5508 808.1509 -395.7754

Random effects:

Formula: ~1 | patientid

(Intercept) Residual

StdDev: 7.136143e-05 1.143905

Fixed effects: gsk3b.meth ~ 1

Value Std.Error DF t-value p-value

(Intercept) 2.165354 0.07177497 134 30.16865 0

Standardized Within-Group Residuals:

Min Q1 Med Q3 Max

-1.8929497 -1.0187511 -0.1445525 0.7296461 3.3522419

Number of Observations: 254

Number of Groups: 120

>

> qqnorm(model.methylation.gsk3b.a.reml, abline=c(0,1))

>

> #CTNNB1 model

>

> model.methylation.ctnnb1.a <- lme(ctnnb1.meth ~ 1, random=~1|patientid,

+ data=dataset.final, na.action=na.omit, method="ML")

>

> model.methylation.ctnnb1.b <- lme(ctnnb1.meth ~ as.factor(relevel(tis, "LRN")), random=~1|patientid,

+ data=dataset.final, na.action=na.omit, method="ML")

>

> anova(model.methylation.ctnnb1.a, model.methylation.ctnnb1.b)

Model df AIC BIC logLik Test L.Ratio

model.methylation.ctnnb1.a 1 3 1176.562 1187.162 -585.2809

model.methylation.ctnnb1.b 2 8 1182.949 1211.216 -583.4744 1 vs 2 3.613054

p-value

model.methylation.ctnnb1.a

model.methylation.ctnnb1.b 0.6064

>

> model.methylation.ctnnb1.a.reml <- lme(ctnnb1.meth ~ 1, random=~1|patientid,

+ data=dataset.final, na.action=na.omit, method="REML")

>

> summary(model.methylation.ctnnb1.a.reml)

Linear mixed-effects model fit by REML

Data: dataset.final

AIC BIC logLik

1178.466 1189.055 -586.2332

Random effects:

Formula: ~1 | patientid

(Intercept) Residual

StdDev: 0.0004257816 2.450776

Fixed effects: ctnnb1.meth ~ 1

Value Std.Error DF t-value p-value

(Intercept) 4.853755 0.154079 132 31.50173 0

Standardized Within-Group Residuals:

Min Q1 Med Q3 Max

-1.98049680 -0.34836105 0.05967291 0.46770689 6.58821603

Number of Observations: 253

Number of Groups: 121

>

> qqnorm(model.methylation.ctnnb1.a.reml, abline=c(0,1))

>

> #DVL2 model

>

> model.methylation.dvl2.a <- lme(dvl2.meth ~ 1, random=~1|patientid,

+ data=dataset.final, na.action=na.omit, method="ML")

>

> model.methylation.dvl2.b <- lme(dvl2.meth ~ as.factor(relevel(tis, "LRN")), random=~1|patientid,

+ data=dataset.final, na.action=na.omit, method="ML")

>

> anova(model.methylation.dvl2.a, model.methylation.dvl2.b)

Model df AIC BIC logLik Test L.Ratio

model.methylation.dvl2.a 1 3 746.5776 757.2941 -370.2888

model.methylation.dvl2.b 2 8 745.6111 774.1883 -364.8056 1 vs 2 10.96649

p-value

model.methylation.dvl2.a

model.methylation.dvl2.b 0.052

>

> model.methylation.dvl2.a.reml <- lme(dvl2.meth ~ 1, random=~1|patientid,

+ data=dataset.final, na.action=na.omit, method="REML")

>

> summary(model.methylation.dvl2.a.reml)

Linear mixed-effects model fit by REML

Data: dataset.final

AIC BIC logLik

750.314 761.019 -372.157

Random effects:

Formula: ~1 | patientid

(Intercept) Residual

StdDev: 0.1039282 0.9855466

Fixed effects: dvl2.meth ~ 1

Value Std.Error DF t-value p-value

(Intercept) 2.171334 0.06173982 138 35.16911 0

Standardized Within-Group Residuals:

Min Q1 Med Q3 Max

-2.1849717 -0.1954962 -0.1682348 0.8136735 5.8484793

Number of Observations: 263

Number of Groups: 125

>

> qqnorm(model.methylation.dvl2.a.reml, abline=c(0,1))

>

> #CHD1 model

>

> model.methylation.cdh1.a <- lme(cdh1.meth ~ 1, random=~1|patientid,

+ data=dataset.final, na.action=na.omit, method="ML")

>

> model.methylation.cdh1.b <- lme(cdh1.meth ~ as.factor(relevel(tis, "LRN")), random=~1|patientid,

+ data=dataset.final, na.action=na.omit, method="ML")

>

> anova(model.methylation.cdh1.a, model.methylation.cdh1.b)

Model df AIC BIC logLik Test L.Ratio

model.methylation.cdh1.a 1 3 1141.307 1152.001 -567.6536

model.methylation.cdh1.b 2 8 1122.204 1150.720 -553.1020 1 vs 2 29.10321

p-value

model.methylation.cdh1.a

model.methylation.cdh1.b <.0001

>

> model.methylation.cdh1.b.reml <- lme(cdh1.meth ~ as.factor(relevel(tis, "LRN")), random=~1|patientid,

+ data=dataset.final, na.action=na.omit, method="REML")

>

> summary(model.methylation.cdh1.b.reml)

Linear mixed-effects model fit by REML

Data: dataset.final

AIC BIC logLik

1122.428 1150.758 -553.2139

Random effects:

Formula: ~1 | patientid

(Intercept) Residual

StdDev: 0.2596545 2.021414

Fixed effects: cdh1.meth ~ as.factor(relevel(tis, "LRN"))

Value Std.Error DF t-value p-value

(Intercept) 9.000000 0.8320193 132 10.817057 0.0000

as.factor(relevel(tis, "LRN"))Ad -2.178482 0.8955770 132 -2.432490 0.0163

as.factor(relevel(tis, "LRN"))HP -1.373012 1.0064973 132 -1.364149 0.1748

as.factor(relevel(tis, "LRN"))HRN -1.243492 0.8586499 132 -1.448194 0.1499

as.factor(relevel(tis, "LRN"))M -1.639380 0.9944295 132 -1.648564 0.1016

as.factor(relevel(tis, "LRN"))pT -2.657792 0.8573965 132 -3.099840 0.0024

Correlation:

(Intr) a.((,"LRN"))A a.((,"LRN"))HP

as.factor(relevel(tis, "LRN"))Ad -0.929

as.factor(relevel(tis, "LRN"))HP -0.827 0.769

as.factor(relevel(tis, "LRN"))HRN -0.969 0.902 0.802

as.factor(relevel(tis, "LRN"))M -0.837 0.777 0.692

as.factor(relevel(tis, "LRN"))pT -0.970 0.902 0.803

a.((,"LRN"))HR a.((,"LRN"))M

as.factor(relevel(tis, "LRN"))Ad

as.factor(relevel(tis, "LRN"))HP

as.factor(relevel(tis, "LRN"))HRN

as.factor(relevel(tis, "LRN"))M 0.811

as.factor(relevel(tis, "LRN"))pT 0.941 0.812

Standardized Within-Group Residuals:

Min Q1 Med Q3 Max

-3.3464727 -0.6412823 0.0000000 0.6419211 4.4866106

Number of Observations: 261

Number of Groups: 124

>

> qqnorm(model.methylation.cdh1.b.reml, abline=c(0,1))

>

> #SOX17 model

>

> model.methylation.sox17.a <- lme(sox17.meth ~ 1, random=~1|patientid,

+ data=dataset.final, na.action=na.omit, method="ML")

>

> model.methylation.sox17.b <- lme(sox17.meth ~ as.factor(relevel(tis, "LRN")), random=~1|patientid,

+ data=dataset.final, na.action=na.omit, method="ML")

>

> anova(model.methylation.sox17.a, model.methylation.sox17.b)

Model df AIC BIC logLik Test L.Ratio

model.methylation.sox17.a 1 3 2166.572 2177.112 -1080.2859

model.methylation.sox17.b 2 8 1985.140 2013.247 -984.5699 1 vs 2 191.432

p-value

model.methylation.sox17.a

model.methylation.sox17.b <.0001

>

> model.methylation.sox17.b.reml <- lme(sox17.meth ~ as.factor(relevel(tis, "LRN")), random=~1|patientid,

+ data=dataset.final, na.action=na.omit, method="REML")

>

> summary(model.methylation.sox17.b.reml)

Linear mixed-effects model fit by REML

Data: dataset.final

AIC BIC logLik

1962.712 1990.623 -973.3559

Random effects:

Formula: ~1 | patientid

(Intercept) Residual

StdDev: 4.433788 12.27754

Fixed effects: sox17.meth ~ as.factor(relevel(tis, "LRN"))

Value Std.Error DF t-value p-value

(Intercept) 27.333333 5.329110 127 5.129061 0.0000

as.factor(relevel(tis, "LRN"))Ad 22.587202 5.750272 127 3.928023 0.0001

as.factor(relevel(tis, "LRN"))HP 10.218616 6.438218 127 1.587181 0.1150

as.factor(relevel(tis, "LRN"))HRN -3.194757 5.511521 127 -0.579651 0.5632

as.factor(relevel(tis, "LRN"))M 13.673897 6.615109 127 2.067071 0.0408

as.factor(relevel(tis, "LRN"))pT 27.724474 5.502879 127 5.038176 0.0000

Correlation:

(Intr) a.((,"LRN"))A a.((,"LRN"))HP

as.factor(relevel(tis, "LRN"))Ad -0.927

as.factor(relevel(tis, "LRN"))HP -0.828 0.773

as.factor(relevel(tis, "LRN"))HRN -0.967 0.905 0.809

as.factor(relevel(tis, "LRN"))M -0.806 0.747 0.667

as.factor(relevel(tis, "LRN"))pT -0.968 0.903 0.808

a.((,"LRN"))HR a.((,"LRN"))M

as.factor(relevel(tis, "LRN"))Ad

as.factor(relevel(tis, "LRN"))HP

as.factor(relevel(tis, "LRN"))HRN

as.factor(relevel(tis, "LRN"))M 0.781

as.factor(relevel(tis, "LRN"))pT 0.942 0.782

Standardized Within-Group Residuals:

Min Q1 Med Q3 Max

-2.95832916 -0.57392154 0.03309594 0.66919084 2.15620974

Number of Observations: 248

Number of Groups: 116

>

> qqnorm(model.methylation.sox17.b.reml, abline=c(0,1))

>
