# Supplementary figures and images for: Boosting Wnt activity during colorectal cancer progression through selective hypermethylation of Wnt signaling antagonists
Source: BMC Cancer. 2014 Nov 29;14:891. doi: 10.1186/1471-2407-14-891 (PMC4265460; doi:10.1186/1471-2407-14-891)

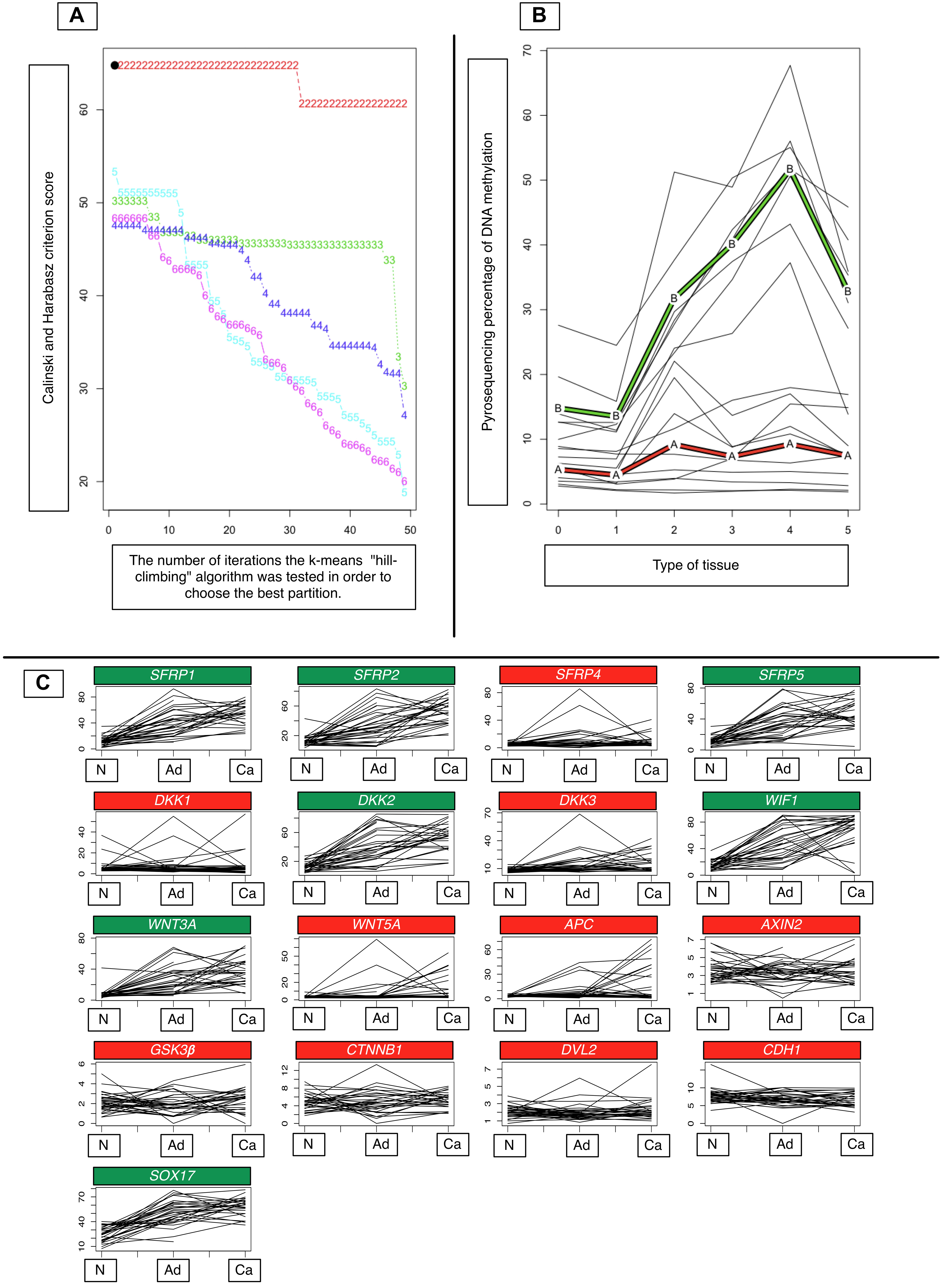

Supplement: Supplementary file 6 — Additional file 6: Is a group of three figures representing the cluster analysis using KmL package (k-means for longitudinal data), ( A ) Calinski&Harabasz criterion highest score was two. (B) Mean trajectories of each cluster, A in red and B in green (0-LRN, 1-HRN, 2-hyperplastic polyps, 3-adenomatous polyps, 4-primary carcinoma, 5-metastatic adenocarcinoma). (C) Methylation percentages for each CpG island clustered according to Calinski&Harabasz criterion (A in red, B in green). (JPEG 3 MB) [file 12885_2014_5079_MOESM6_ESM.jpeg]

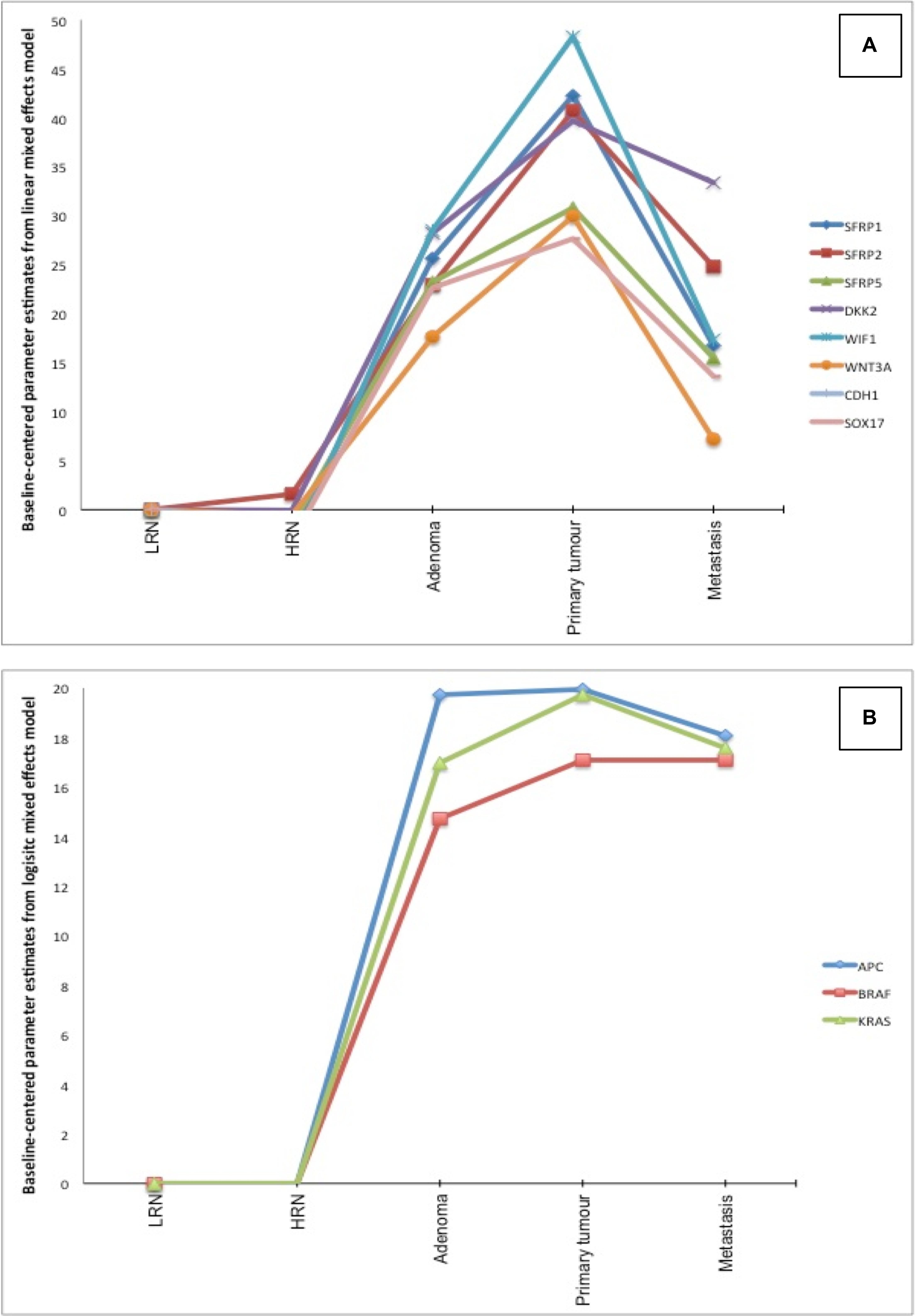

Supplement: Supplementary file 8 — Additional file 8: Includes two figures of the estimates from the linear mixed effect model for methylation (A) and logistic mixed effect model for mutations (B) through the neoplastic progression. All mutations are present by the adenoma stage while methylation gains continue to increase up to the carcinoma stage showing a considerable reduction at the metastasis stage. (JPEG 1 MB) [file 12885_2014_5079_MOESM8_ESM.jpeg]
