# Supplementary material for: Boosting Wnt activity during colorectal cancer progression through selective hypermethylation of Wnt signaling antagonists
Source: BMC Cancer. 2014 Nov 29;14:891. doi: 10.1186/1471-2407-14-891 (PMC4265460; doi:10.1186/1471-2407-14-891)
Supplement: Supplementary file 7 — Additional file 7: Is a table with the results of the Wilcoxon signed rank test or paired t-test depending on data distribution and false discovery rate adjusted for multiple testing. (DOCX 83 KB) [file 12885_2014_5079_MOESM7_ESM.docx]

**Additional data file 7**

Wilcoxon signed rank test or paired t-test

|  | tissue | tissue | p value | fdr-adjusted pvalues |
| --- | --- | --- | --- | --- |
| SFRP1 | HRN | Adenoma | 0.000 | 0.000 |
| SFRP5 | HRN | Adenoma | 0.000 | 0.000 |
| SOX17 | HRN | Adenoma | 0.000 | 0.000 |
| WIF1 | HRN | Adenoma | 0.000 | 0.000 |
| DKK2 | HRN | Adenoma | 0.000 | 0.000 |
| WNT3A | HRN | Adenoma | 0.000 | 0.000 |
| SFRP2 | HRN | Adenoma | 0.000 | 0.000 |
| DKK3 | HRN | Adenoma | 0.000 | 0.001 |
| SFRP2 | Adenoma | Carcinoma | 0.001 | 0.003 |
| WIF1 | Adenoma | Carcinoma | 0.003 | 0.009 |
| DKK2 | Adenoma | Carcinoma | 0.004 | 0.012 |
| SFRP1 | Adenoma | Carcinoma | 0.004 | 0.012 |
| APC | HRN | Adenoma | 0.015 | 0.040 |
| SFRP4 | HRN | Adenoma | 0.025 | 0.060 |
| WNT3A | Adenoma | Carcinoma | 0.030 | 0.068 |
| CTNNB1 | HRN | Adenoma | 0.054 | 0.108 |
| GSK3B | Adenoma | Carcinoma | 0.052 | 0.108 |
| SFRP5 | Adenoma | Carcinoma | 0.065 | 0.124 |
| WNT5A | Adenoma | Carcinoma | 0.106 | 0.189 |
| WNT5A | HRN | Adenoma | 0.121 | 0.206 |
| SOX17 | Adenoma | Carcinoma | 0.129 | 0.209 |
| DVL2 | Adenoma | Carcinoma | 0.136 | 0.211 |
| DVL2 | HRN | Adenoma | 0.245 | 0.363 |
| CDH1 | HRN | Adenoma | 0.304 | 0.431 |
| GSK3B | HRN | Adenoma | 0.458 | 0.537 |
| AXIN2 | Adenoma | Carcinoma | 0.443 | 0.537 |
| CDH1 | Adenoma | Carcinoma | 0.449 | 0.537 |
| CTNNB1 | Adenoma | Carcinoma | 0.452 | 0.537 |
| DKK3 | Adenoma | Carcinoma | 0.413 | 0.537 |
| APC | Adenoma | Carcinoma | 0.500 | 0.566 |
| AXIN2 | HRN | Adenoma | 0.761 | 0.834 |
| DKK1 | HRN | Adenoma | 0.958 | 0.980 |
| DKK1 | Adenoma | Carcinoma | 0.953 | 0.980 |
| SFRP4 | Adenoma | Carcinoma | 0.980 | 0.980 |
