# Supplementary material for: Boosting Wnt activity during colorectal cancer progression through selective hypermethylation of Wnt signaling antagonists
Source: BMC Cancer. 2014 Nov 29;14:891. doi: 10.1186/1471-2407-14-891 (PMC4265460; doi:10.1186/1471-2407-14-891)
Supplement: Supplementary file 9 — Additional file 9: Is a table listing the APC mutations and LOH status of the samples used in this study. (DOCX 140 KB) [file 12885_2014_5079_MOESM9_ESM.docx]

**Additional data file 9 –** APC mutations and LOH status of the samples included in this study

| original_uniq_id | APC_Mut_Prsent/Absent | APC_Mut_type | APC_LOH | APC_chr5:112_101_334-112_101_537. average Level of methylation | tissue |
| --- | --- | --- | --- | --- | --- |
| CRC1_LT1 | Absent | NA | non informative | 3 | M |
| CRC1_LT10 | Absent | NA | non informative | 5 | M |
| CRC1_LT11 | Absent | NA | non informative | 4 | M |
| CRC1_LT12 | Absent | NA | non informative | 3 | M |
| CRC1_LT13 | Absent | NA | no LOH | 4 | M |
| CRC1_LT2 | Absent | NA | LOH | 23 | M |
| CRC1_LT3 | Present | p.E1309* | non informative | 79 | M |
| CRC1_LT4 | Absent | NA | no LOH | 18 | M |
| CRC1_LT5 | Present | p.Q1228*; p.R1450* | no LOH | 5 | M |
| CRC1_LT6 | Absent | NA | no LOH | 8 | M |
| CRC1_LT7 | Present | p.Q1294* | no LOH | 4 | M |
| CRC1_LT8 | Absent | NA | non informative | 47 | M |
| CRC1_LT9 | Absent | NA | non informative | 3 | M |
| CRC1_n1102 | Absent | NA | NA | 3 | HRN |
| CRC1_n1249 | Absent | NA | NA | 5 | HRN |
| CRC1_NC10 | Absent | NA | NA | 2 | HRN |
| CRC1_NC11 | Absent | NA | NA | 2 | HRN |
| CRC1_NC12 | Absent | NA | NA | 3 | HRN |
| CRC1_NC13 | Absent | NA | NA | 3 | HRN |
| CRC1_NC14 | Absent | NA | NA | 3 | HRN |
| CRC1_NC15 | Absent | NA | NA | 3 | HRN |
| CRC1_NC16 | Absent | NA | NA | 3 | HRN |
| CRC1_NC17 | Absent | NA | NA | 2 | HRN |
| CRC1_NC18 | Absent | NA | NA | 2 | HRN |
| CRC1_NC19 | Absent | NA | NA | 2 | HRN |
| CRC1_NC20 | Absent | NA | NA | 2 | HRN |
| CRC1_NC21 | Absent | NA | NA | 4 | HRN |
| CRC1_NC22 | Absent | NA | NA | 2 | HRN |
| CRC1_NC23 | Absent | NA | NA | 3 | HRN |
| CRC1_NC6 | Absent | NA | NA | 2 | HRN |
| CRC1_NC7 | Absent | NA | NA | 5 | HRN |
| CRC1_NC8 | Absent | NA | NA | 4 | HRN |
| CRC1_NC9 | Absent | NA | NA | 2 | HRN |
| CRC1_T1053 | Present | p.Q1378* | NA | 2 | Ca |
| CRC1_T1094 | NA | NA | NA | 28 | Ca |
| CRC1_T1102 | NA | NA | NA | 39 | Ca |
| CRC1_T1129 | Present | p.K1449fs*34 | NA | 82 | Ca |
| CRC1_T1197 | Present | p.I1307K; p.E1309fs*6 | NA | 4 | Ca |
| CRC1_T1202 | NA | NA | NA | 2 | Ca |
| CRC1_T1239 | Present | p.L1488fs*19 | NA | 46 | Ca |
| CRC1_T1249 | NA | NA | NA | 3 | Ca |
| CRC1_T1293 | NA | NA | NA | 2 | Ca |
| CRC1_T1311 | NA | NA | NA | 5 | Ca |
| CRC1_T1396 | Present | p.K1370* | NA | 2 | Ca |
| CRC1_T1479 | NA | NA | NA | 3 | Ca |
| CRC1_T1500 | Present | p.P1439fs*34 | NA | 1 | Ca |
| CRC1_T1501 | Present | p.V1352fs*2; p.E1353F | NA | 2 | Ca |
| CRC1_T1621 | Present | p.N1455fs*18 | NA | 5 | Ca |
| CRC1_T165 | Absent | NA | NA | 8 | Ca |
| CRC1_T166 | Present | p.R1450* | NA | 1 | Ca |
| CRC1_T1843 | Absent | NA | NA | 16 | M |
| CRC1_T1869 | Present | p.S1355fs*60 | NA | 2 | Ca |
| CRC1_T220 | Present | p.D1394fs*2 | NA | 4 | Ca |
| CRC1_T2444 | Absent | NA | NA | 3 | Ca |
| CRC1_T2528 | Present | p.H1490fs*12 | NA | 19 | Ca |
| CRC1_T2688 | Absent | NA | NA | 2 | Ca |
| CRC1_T2700 | Absent | NA | NA | 1 | Ca |
| CRC1_T2715 | Absent | NA | NA | 2 | Ca |
| CRC1_T309 | Present | p.T1430fs*44 | NA | 39 | Ca |
| CRC1_T3144 | Absent | NA | NA | 3 | M |
| CRC1_T3463 | Absent | NA | NA | 5 | Ca |
| CRC1_T377 | Present | p.E1317Q; p.Q1549* | NA | 43 | Ca |
| CRC1_T420 | Present | p.A1358fs*6 | NA | 3 | Ca |
| CRC1_T451 | Absent | NA | NA | 2 | Ca |
| CRC1_T565 | Present | p.L1489fs*18 | NA | 1 | Ca |
| CRC1_T625 | Present | p.S1364fs*15 | NA | 3 | Ca |
| CRC1_T771 | Present | p.S1356* | NA | 4 | Ca |
| CRC1_T826 | Absent | NA | NA | 16 | Ca |
| CRC1_T932 | Present | p.P1439fs*34 | NA | 1 | Ca |
| CRC1_T940 | Present | p.E1317Q | NA | 2 | Ca |
| CRC1_TC10 | Present | p.E1306fs*10 | no LOH | 70 | Ca |
| CRC1_TC11 | Absent | NA | no LOH | 6 | Ca |
| CRC1_TC12 | Absent | NA | non informative | 15 | Ca |
| CRC1_TC13 | Present | p.R1450* | non informative | 82 | Ca |
| CRC1_TC14 | Absent | NA | no LOH | 2 | Ca |
| CRC1_TC15 | Present | p.E1451* | non informative | 25 | Ca |
| CRC1_TC16 | Absent | NA | no LOH | 6 | Ca |
| CRC1_TC17 | Present | p.T1556fs*3 | no LOH | 2 | Ca |
| CRC1_TC18 | Present | p.R1450* | non informative | 2 | Ca |
| CRC1_TC19 | Present | p.Q1228*; p.R1450* | no LOH | 10 | Ca |
| CRC1_TC20 | Absent | NA | non informative | 89 | Ca |
| CRC1_TC21 | Present | p.E1554fs*5 | no LOH | 3 | Ca |
| CRC1_TC22 | Present | p.S1400* | non informative | 4 | Ca |
| CRC1_TC23 | Absent | NA | no LOH | 2 | Ca |
| CRC1_TC7 | NA | NA | NA | 2 | Ca |
| CRC1_TC8 | Present | p.Q1477fs*30 | non informative | 15 | Ca |
| CRC2_001NA | Absent | NA | NA | 2 | HRN |
| CRC2_001NC | Absent | NA | NA | 3 | HRN |
| CRC2_001T1 | Absent | NA | no LOH | 2 | Ca |
| CRC2_002Ad2 | Absent | NA | no LOH | 2 | Ad |
| CRC2_002Ad3 | Absent | NA | no LOH | 1 | Ad |
| CRC2_002NA | Absent | NA | NA | 2 | HRN |
| CRC2_002NC | Absent | NA | NA | 3 | HRN |
| CRC2_002T1 | Absent | NA | LOH | 15 | Ca |
| CRC2_003Ncol | Absent | NA | NA | 6 | HRN |
| CRC2_003T1 | Absent | NA | no LOH | 3 | Ca |
| CRC2_003T2 | Absent | NA | no LOH | 2 | Ca |
| CRC2_004Ncol | Absent | NA | NA | 3 | HRN |
| CRC2_004T1 | Absent | NA | no LOH | 2 | Ca |
| CRC2_004T2 | Absent | NA | no LOH | 3 | Ca |
| CRC2_005NA | Absent | NA | NA | 3 | HRN |
| CRC2_005NC | Absent | NA | NA | 3 | HRN |
| CRC2_005T1 | Absent | NA | LOH | 2 | Ca |
| CRC2_006Ad1 | Absent | NA | non informative | 3 | HP |
| CRC2_006NA | Absent | NA | NA | 4 | HRN |
| CRC2_006NC | Absent | NA | NA | 4 | HRN |
| CRC2_006T1 | Present | p.D1498 fs*19 | non informative | 2 | Ca |
| CRC2_007NA | Absent | NA | NA | 6 | HRN |
| CRC2_007NC | Absent | NA | NA | 1 | HRN |
| CRC2_007T1 | Present | p.E1309fs*4 | non informative | 3 | Ca |
| CRC2_008Ad1 | Absent | NA | non informative | 3 | HP |
| CRC2_008Ad2 | Absent | NA | non informative | 3 | HP |
| CRC2_008Ad3 | Present | p.Q1294fs*11 | non informative | 35 | Ad |
| CRC2_008NA | Absent | NA | NA | 4 | HRN |
| CRC2_008NC | Absent | NA | NA | 4 | HRN |
| CRC2_008T1 | Absent | NA | non informative | 27 | Ca |
| CRC2_009Ad1 | Present | p.E1306* | non informative | 4 | HP |
| CRC2_009Ad2 | Absent | NA | non informative | 2 | Ad |
| CRC2_009Ad3 | Absent | NA | non informative | 2 | Ad |
| CRC2_009Ad5 | Present | p.E1306* | non informative | 3 | Ad |
| CRC2_009Ncol | Absent | NA | NA | 2 | HRN |
| CRC2_010Ad1 | Present | p.S1426 fs*45 | non informative | 44 | Ad |
| CRC2_010Ncol | Absent | NA | NA | 4 | HRN |
| CRC2_010T1 | Absent | NA | non informative | 49 | Ca |
| CRC2_011Ad1 | Present | p.E1309fs*4 | non informative | 5 | Ad |
| CRC2_011Ncol | Absent | NA | NA | 4 | HRN |
| CRC2_011T1 | Present | p.R1450* | non informative | 73 | Ca |
| CRC2_013Ad1 | Present | p.R1450* | non informative | 7 | Ad |
| CRC2_013Ncol | Absent | NA | NA | 3 | HRN |
| CRC2_013T1 | Present | p.R1450* | non informative | 1 | Ca |
| CRC2_014Ad1 | Absent | NA | non informative | 2 | HP |
| CRC2_014Ncol | Absent | NA | NA | 6 | HRN |
| CRC2_014T1 | Present | p.S1321fs*10 | non informative | 45 | Ca |
| CRC2_015NA | Absent | NA | NA | 2 | HRN |
| CRC2_015NC | Absent | NA | NA | 3 | HRN |
| CRC2_015T1 | Absent | NA | non informative | 16 | Ca |
| CRC2_016Ad1 | Absent | NA | no LOH | 5 | Ad |
| CRC2_016NA | Absent | NA | NA | 3 | HRN |
| CRC2_016NC | Absent | NA | NA | 1 | HRN |
| CRC2_016T1 | Absent | NA | no LOH | 2 | Ca |
| CRC2_017Ad1 | Absent | NA | non informative | 4 | HP |
| CRC2_017NA | Absent | NA | NA | 3 | HRN |
| CRC2_017NC | Absent | NA | NA | 3 | HRN |
| CRC2_017T1 | Absent | NA | non informative | 27 | Ca |
| CRC2_018Ad1 | Absent | NA | no LOH | 4 | Ad |
| CRC2_018Ncol | Absent | NA | NA | 3 | HRN |
| CRC2_018T1 | Absent | NA | no LOH | 2 | Ca |
| CRC2_019Ad1 | Absent | NA | no LOH | 2 | Ad |
| CRC2_019Ad2 | Present | p.M1383fs*3 | LOH | 3 | Ad |
| CRC2_019NA | Absent | NA | NA | 1 | HRN |
| CRC2_019NC | Absent | NA | NA | 3 | HRN |
| CRC2_020Ad1 | Absent | NA | no LOH | 1 | Ad |
| CRC2_020NA | Absent | NA | NA | 3 | HRN |
| CRC2_020NC | Absent | NA | NA | 3 | HRN |
| CRC2_021Ad1 | Absent | NA | non informative | 3 | HP |
| CRC2_021NA | Absent | NA | NA | 3 | HRN |
| CRC2_021T1 | Absent | NA | non informative | 41 | Ca |
| CRC2_022Ad1 | Absent | NA | non informative | 3 | HP |
| CRC2_022NA | Absent | NA | NA | 4 | HRN |
| CRC2_022T1 | Absent | NA | non informative | 52 | Ca |
| CRC2_023Ad1 | Present | p.Y1376fs*1 | non informative | 11 | Ad |
| CRC2_023NA | Absent | NA | NA | 3 | HRN |
| CRC2_023NC | Absent | NA | NA | 3 | HRN |
| CRC2_023T1 | Present | p.P1369fs*5 | non informative | 2 | Ca |
| CRC2_024Ad1 | Absent | NA | no LOH | 3 | HP |
| CRC2_024NA | Absent | NA | NA | 4 | HRN |
| CRC2_024NC | Absent | NA | NA | 3 | HRN |
| CRC2_024T1 | Absent | NA | no LOH | 4 | Ca |
| CRC2_025Ad1 | Present | p.E1322* | no LOH | 39 | Ad |
| CRC2_025NC | Absent | NA | NA | 3 | HRN |
| CRC2_025T1 | Present | p.Q1378* | no LOH | 2 | Ca |
| CRC2_026Ad1 | Present | p.V1414fs*1 | no LOH | 15 | Ad |
| CRC2_026NA | Absent | NA | NA | 4 | HRN |
| CRC2_026NC | Absent | NA | NA | 3 | HRN |
| CRC2_026T1 | Present | p.Q1291* | no LOH | 5 | Ca |
| CRC2_027Ad1 | Present | p.Q1429* | no LOH | 4 | Ad |
| CRC2_027NA | Absent | NA | NA | 3 | HRN |
| CRC2_027NC | Absent | NA | NA | 4 | HRN |
| CRC2_027T1 | Present | p.Q1291* | LOH | 1 | Ca |
| CRC2_029Ad1 | Present | p.T1556fs*3 | non informative | 4 | Ad |
| CRC2_029NA | Absent | NA | NA | 4 | HRN |
| CRC2_029NC | Absent | NA | NA | 3 | HRN |
| CRC2_029T1 | NA | NA | non informative | 37 | Ca |
| CRC2_030Ad1 |  | p.P1319fs*2 | non informative | 9 | Ad |
| CRC2_030NA | Absent | NA | NA | 3 | HRN |
| CRC2_030NC | Absent | NA | NA | 3 | HRN |
| CRC2_030T1 | Absent | NA | non informative | 4 | Ca |
| CRC2_031Ad1 | Present | p.R1399fs*11 | non informative | 4 | Ad |
| CRC2_031Ncol | Absent | NA | NA | 3 | HRN |
| CRC2_031T1 | Present | p.Q1367* | non informative | 42 | Ca |
| CRC2_032Ad1 | Absent | NA | no LOH | 2 | Ad |
| CRC2_032Ncol | Absent | NA | NA | 4 | HRN |
| CRC2_032T1 | Present | p.R1450fs*23 | no LOH | 4 | Ca |
| CRC2_033Ad1 | Absent | NA | no LOH | 4 | Ad |
| CRC2_033Ncol | Absent | NA | NA | 3 | HRN |
| CRC2_033T1 | Absent | NA | no LOH | 11 | Ca |
| CRC2_034Ad1 | Absent | NA | no LOH | 6 | Ad |
| CRC2_034Ncol | Absent | NA | NA | 3 | HRN |
| CRC2_034T1 | Present | p.E1577fs*73 | no LOH | 42 | Ca |
| CRC2_035Ad1 | Absent | NA | non informative | 14 | HP |
| CRC2_035NA | Absent | NA | NA | 4 | HRN |
| CRC2_035NC | Absent | NA | NA | 4 | HRN |
| CRC2_035T1 | Present | p.N1229fs*11 | non informative | 14 | Ca |
| CRC2_036Ad1 | Absent | NA | no LOH | 2 | Ad |
| CRC2_036Ncol | Absent | NA | NA | 4 | HRN |
| CRC2_036T1 | Present | p.T1556fs*3 | no LOH | 3 | Ca |
| CRC2_037Ad1 | Present | p.R1450* | no LOH | 5 | Ad |
| CRC2_037NA | Absent | NA | NA | 3 | HRN |
| CRC2_037NC | Absent | NA | NA | 2 | HRN |
| CRC2_037T1 | Absent | NA | no LOH | 3 | Ca |
| CRC2_038Ad1 | Present | p.V1472fs*35 | non informative | 2 | Ad |
| CRC2_038Ncol | Absent | NA | NA | 2 | HRN |
| CRC2_038T1 | Present | p.S1411fs*4 | non informative | 1 | Ca |
| CRC2_039Ncol | Absent | NA | NA | 3 | HRN |
| CRC2_039T1 | Present | p.S1400* | non informative | 0 | Ca |
| CRC2_039T2 | Present | p.P1424fs*49 | non informative | 19 | Ca |
| CRC2_040Ad1 | Absent | NA | no LOH | 4 | Ad |
| CRC2_040NA | Absent | NA | NA | 2 | HRN |
| CRC2_040NC | Absent | NA | NA | 4 | HRN |
| CRC2_040T1 | Present | p.T1556fs*3 | no LOH | 61 | Ca |
| CRC2_041Ad1 | Present | p.R1227fs*13 | no LOH | 3 | Ad |
| CRC2_041NA | Absent | NA | NA | 3 | HRN |
| CRC2_041NC | Absent | NA | NA | NA | HRN |
| CRC2_041T1 | NA | NA | NA | 2 | Ca |
| CRC2_042Ad1 | Absent | NA | no LOH | 3 | Ad |
| CRC2_042NA | Absent | NA | NA | 3 | HRN |
| CRC2_042NC | Absent | NA | NA | 3 | HRN |
| CRC2_042T1 | Present | p.K1308* | LOH | 3 | Ca |
| CRC2_043Ad1 | Absent | NA | non informative | 2 | HP |
| CRC2_043Ncol | Absent | NA | NA | 3 | HRN |
| CRC2_043T1 | Absent | NA | non informative | 11 | Ca |
| CRC2_044Ad1 | Present | p.L1488fs*19 | non informative | 9 | Ad |
| CRC2_044NA | Absent | NA | NA | 2 | HRN |
| CRC2_044NC | Absent | NA | NA | 1 | HRN |
| CRC2_044T1 | Present | p.L1277fs*11 | non informative | 3 | Ca |
| CRC2_045Ad1 | Present | p.E1322* | no LOH | 2 | Ad |
| CRC2_045Ncol | Absent | NA | NA | 5 | HRN |
| CRC2_045T1 | Present | p.R1450* | no LOH | 66 | Ca |
| CRC2_046Ad1 | Absent | NA | no LOH | 5 | Ad |
| CRC2_046Ncol | Absent | NA | NA | 6 | HRN |
| CRC2_046T1 | Absent | NA | no LOH | 2 | Ca |
| CRC2_047Ad1 |  | p.E1540* | non informative | 2 | Ad |
| CRC2_047Ncol | Absent | NA | NA | 4 | HRN |
| CRC2_047T1 | Present | p.T1556fs*3 | non informative | 29 | Ca |
| CRC2_048Ad1 | Absent | NA | non informative | 2 | Ad |
| CRC2_048Ad2 | Absent | NA | non informative | 3 | Ad |
| CRC2_048Ncol | Absent | NA | NA | 5 | HRN |
| CRC2_049Ad1 | Present | p.S1415fs*4 | no LOH | 5 | HP |
| CRC2_049Ncol | Absent | NA | NA | 2 | HRN |
| CRC2_049T1 | Present | p.R1399fs*10 | LOH | 52 | Ca |
| CRC2_053Ad1 | Absent | NA | no LOH | 2 | HP |
| CRC2_053Ncol | Absent | NA | NA | 1 | HRN |
| CRC2_053T1 | Present | p.S1421fs*1 | no LOH | 2 | Ca |
| CRC2_055Ad1 | Absent | NA | no LOH | 2 | Ad |
| CRC2_055Ncol | Absent | NA | NA | 4 | HRN |
| CRC2_055T1 | Present | p.Q1244* | LOH | 3 | Ca |
| CRC2_056Ad1 | Present | p.Q1328* | non informative | 8 | Ad |
| CRC2_056Ncol | Absent | NA | NA | 2 | HRN |
| CRC4_D01 | NA | NA | NA | 4 | LRN |
| CRC4_D02 | NA | NA | NA | 11 | LRN |
| CRC4_D05 | NA | NA | NA | 3 | LRN |
| CRC4_D06 | NA | NA | NA | 3 | LRN |
| CRC4_D07 | NA | NA | NA | 7 | LRN |
| CRC4_D08 | NA | NA | NA | 4 | LRN |
