# Supplementary material for: Boosting Wnt activity during colorectal cancer progression through selective hypermethylation of Wnt signaling antagonists
Source: BMC Cancer. 2014 Nov 29;14:891. doi: 10.1186/1471-2407-14-891 (PMC4265460; doi:10.1186/1471-2407-14-891)
Supplement: Supplementary file 10 — Additional file 10: Is a table of the results of the point-biserial correlation test r pb value and p value of the correlation between DNA methylation of each gene and MSI/MSI in primary tumours. (DOCX 70 KB) [file 12885_2014_5079_MOESM10_ESM.docx]

| **Additional data file 10**  Point-biserial correlation test *r_pb_* value and *p*value of the correlation between DNA methylation of each gene and MSI/MSI in primary tumours. Significant correlations are highlighted in bold. | | | | | | | | | | | | | | | |
| --- | --- | --- | --- | --- | --- | --- | --- | --- | --- | --- | --- | --- | --- | --- | --- |
|  | | Microsatellite Status | | | | | |  | | CIMP Status | | | |  | |
|  | rpb-value | | p-value | | fdr-adjusted p-values | |  | | rpb-value | | p-value | | fdr-adjusted p-values | |  |
| SOX17 | | -0.109 | | 0.388 | | 0.624 | |  | | 0.077 | | 0.483 | | 0.632 | |
| SFRP1 | | -0.069 | | 0.581 | | 0.706 | |  | | 0.081 | | 0.457 | | 0.632 | |
| SFRP2 | | 0.007 | | 0.954 | | 0.954 | |  | | 0.086 | | 0.410 | | 0.632 | |
| SFRP4 | | **0.511** | | **0.000** | | **0.000** | |  | | 0.273 | | 0.019 | | 0.054 | |
| SFRP5 | | 0.160 | | 0.180 | | 0.510 | |  | | **0.274** | | **0.008** | | **0.034** | |
| DKK1 | | **0.506** | | **0.000** | | **0.000** | |  | | **0.496** | | **0.000** | | **0.000** | |
| DKK2 | | -1.236 | | 0.308 | | 0.624 | |  | | 0.015 | | 0.889 | | 0.945 | |
| DKK3 | | 0.209 | | 0.092 | | 0.391 | |  | | 0.274 | | 0.010 | | 0.034 | |
| WIF1 | | 0.083 | | 0.493 | | 0.698 | |  | | 0.166 | | 0.112 | | 0.272 | |
| WNT3A | | 0.117 | | 0.337 | | 0.624 | |  | | 0.006 | | 0.287 | | 0.542 | |
| WNT5A | | **0.474** | | **0.000** | | **0.000** | |  | | **0.474** | | 0.000 | | 0.000 | |
| APC | | -0.032 | | 0.793 | | 0.843 | |  | | **0.967** | | 0.004 | | 0.023 | |
| AXIN2 | | 0.076 | | 0.538 | | 0.704 | |  | | -0.097 | | 0.361 | | 0.614 | |
| GSK3B | | 0.167 | | 0.176 | | 0.510 | |  | | -0.049 | | 0.649 | | 0.736 | |
| CDH1 | | 0.134 | | 0.267 | | 0.624 | |  | | -0.056 | | 0.596 | | 0.724 | |
| CTNNB1 | | 0.103 | | 0.404 | | 0.624 | |  | | -0.124 | | 0.246 | | 0.523 | |
| DVL2 | | 0.057 | | 0.635 | | 0.720 | |  | | 0.006 | | 0.952 | | 0.952 | |
